# Arene extrusion as an approach to reductive elimination at boron: implication of carbene-ligated haloborylene as a transient reactive intermediate†

**DOI:** 10.1039/d4sc05524a

**Published:** 2024-10-03

**Authors:** Chonghe Zhang, Robert J. Gilliard, Christopher C. Cummins

**Affiliations:** a Department of Chemistry, Massachusetts Institute of Technology Cambridge Massachusetts 02139 USA ccummins@mit.edu gilliard@mit.edu

## Abstract

Herein, we report boron-centered arene extrusion reactions to afford putative cyclic(alkyl)(amino) carbene (CAAC)-ligated chloroborylene and bromoborylene intermediates. The borylene precursors, chloro-boranorbornadiene (ClB(C_6_Me_6_), 2^Cl^) and bromo-boranorbornadiene (BrB(C_6_Me_6_), 2^Br^) were synthesized through the reaction of the corresponding 1-halo-2,3,4,5-tetramethylborole dimer (XBC_4_Me_4_)_2_ (X = Cl, 1^Cl^; X = Br, 1^Br^) with 2-butyne. Treatment of 2^Cl^ with CAACs resulted in the release of di-coordinate chloro-borylene (CAAC)BCl from hexamethylbenzene (C_6_Me_6_) at room temperature. In contrast, the reaction of 2^Br^ with CAAC led to the formation of a boronium species [(CAAC)BC_6_Me_6_]^+^Br^−^ (7) at room temperature. Heating 7 in toluene promoted the release of di-coordinate bromo-borylene (CAAC)BBr as a transient species. Surprisingly, heating 7 in dichloromethane resulted in the C–H activation of hexamethylbenzene. The conversion of a CAAC-stabilized bromo-borepin to a borylene, a boron-centered retro Büchner reaction, was also investigated.

## Abstract 2

Herein, we report boron-centered reductive elimination reactions to afford cyclic(alkyl)(amino) carbene (CAAC)-ligated chloroborylene and bromoborylene.

## Graphical abstract

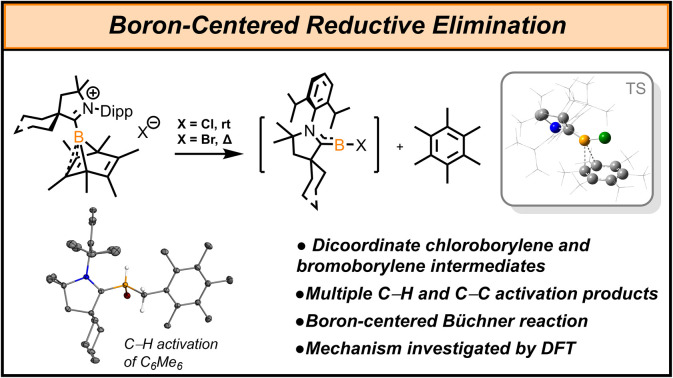

## 1 Introduction

**Fig. 1:**
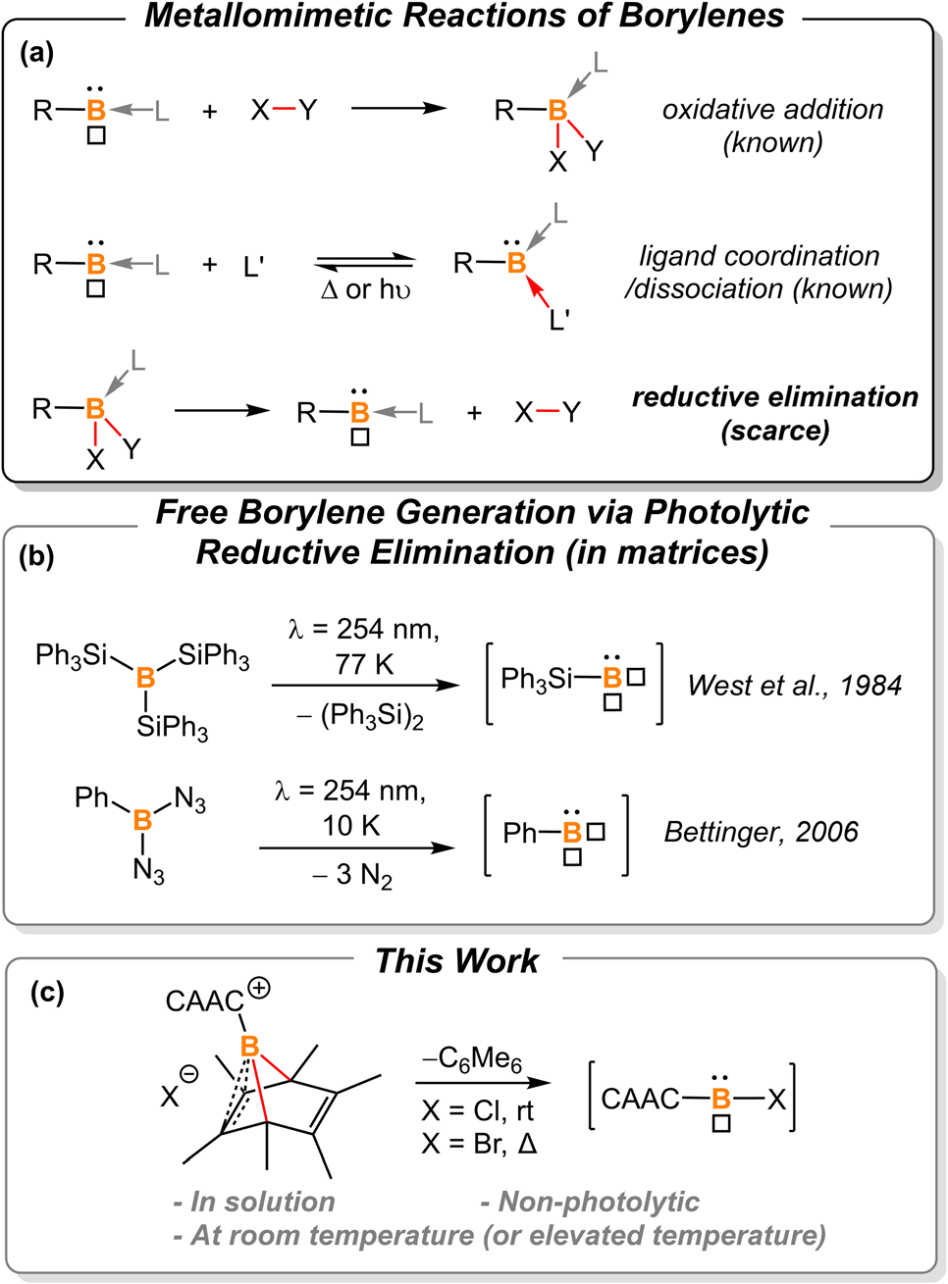
(a) Summarized reactions of borylenes, “□” represents the empty p-orbital of boron. (b) Previously reported photolytic reductive elimination to afford free borylenes; (c) borylene release from hexamethylboranorbornadiene.

Borylenes are boron analogs of carbenes. Free borylenes (with the chemical formula R–B) are monocoordinate species that possess only four valence electrons, representing a class of hypovalent main-group species.^1^ While free monocoordinate borylenes only exist as highly reactive intermediates, Lewis-base stabilized borylenes are easier to handle, with some even being isolable at room temperature.^2^ Dicoordinate borylenes are conceptually expected to possess both a non-bonding electron pair and an empty p orbital, thus constituting a key class of metallomimetic boron species.^3^ Owing to the low-coordinate nature of borylenes, these B(i) species readily undergo oxidative addition reactions to form stable B(iii)-centered molecules (Fig. 1a, top).^2*b*,*g*,2*j*,4^ The coordination of a ligand is also known to stabilize reactive borylene species. This ligand coordination/dissociation process mimics reaction steps that are common for transition metals (Fig. 1a, middle).^2*g*,*j*,5^ However, it is rationalized that B(iii) species are less likely to undergo reductive elimination to afford corresponding borylenes due to the low electronegativity, small radius, and inherent electron-deficient nature of the boron atom (Fig. 1a, bottom). Indeed, to the best of our knowledge, there are only two examples of reductive elimination occurring at a single boron atom.

In 1984, West *et al.* reported that photolysis of B(SiPh_3_)_3_ in a matrix resulted in the formation of triphenylsilylborylene (Ph_3_SiB) by elimination of equimolar Ph_3_Si–SiPh_3_ (Fig. 1b).^1*k*^ In 2006, Bettinger described that the photolysis of bisazidophenylborane (PhB(N_3_)_2_) isolated in cryogenic matrices results in phenylborylene (PhB).^1*j*^ These two examples demonstrate that reductive elimination can take place *via* photolysis of energetic B(iii) compounds in matrices. However, a fundamental question still remains elusive: can boron behave like transition metals that undergo thermal reductive elimination?

In the last decade, the Cummins group developed a series of dibenzo-7-phosphanorbornadiene compounds, RPA (A = C_14_H_10_ or anthracene).^6^ Depending on the nature of the substituent, some of these compounds eliminate one equivalent of anthracene and release a reactive phosphinidene species into the solution or gas phase for further reactions or spectroscopic characterization. Such phosphorus-centered arene extrusion reactions are regarded as a particular type of reductive elimination, as the center atom becomes a corresponding subvalent species upon the extrusion of the arene molecule.^6*a*,*b*,7^ We postulate that aromatization could provide the extra driving force for boron-centered reductive elimination.

In 2013, Braunschweig *et al.* investigated the potential of the liberation of NHC-stabilized phenylborylene from (IMe)(Ph)B(C_6_Ph_6_).^8^ However, ring expansion to form NHC-borepin Lewis adducts was found to be the preferred reaction channel and no elimination of an NHC-stabilized borylene was detected. They attributed the observed behavior to molecular strain and steric factors. Therefore, we sought to find a platform with less molecular strain and steric factors for releasing the borylene fragment, and thus hexamethyl-boranorbornadiene was the targeted system (Fig. 1c). Herein, we demonstrate the first example of non-photolytic reductive elimination taking place at a single boron atom to afford putative cyclic(alkyl)(amino) carbene (CAAC)-stabilized haloborylenes.

## 2 Results and discussion

### 2.1 Synthesis of boranorbornadiene

**Scheme 1:**
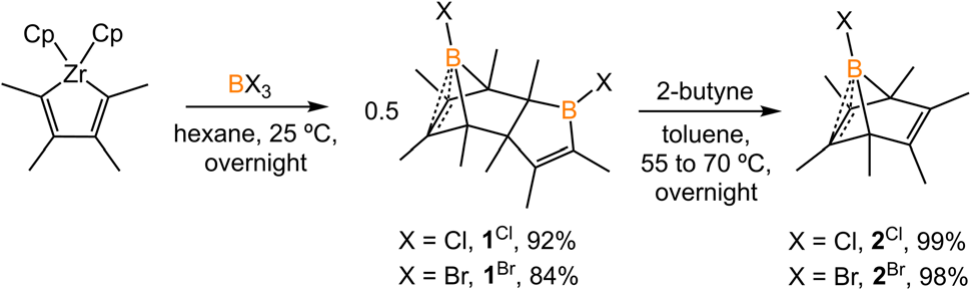
Synthesis of borole dimers (1^Cl^ and 1^Br^) and boranorbornadienes (2^Cl^ and 2^Br^).

The synthesis of 1-phenyl-2,3,4,5-tetramethylborole dimer using a zirconium reagent was developed by Fagan *et al.*^9^ The halogen-substituted borole dimers were obtained in a similar manner. The reaction of zirconium metallacycle Cp_2_Zr(C_4_Me_4_) with BCl_3_ (1.1 equiv.) led to the precipitation of Cp_2_ZrCl_2_ and production of 1-chloro-2,3,4,5-tetramethylborole dimer 1^Cl^ (Scheme 1). The borole dimer 1^Cl^ was separated from the zirconium salt by filtration, purified by recrystallization, and obtained in an excellent yield (92%). Similarly, the same procedure using BBr_3_ afforded 1-bromo-2,3,4,5-tetramethylborole dimer 1^Br^ in 84% yield. The boron atoms of 1^Cl^ and 1^Br^ in vinylic positions are observed as broad singlets in the ^11^B{^1^H} NMR spectra at *δ*_B_ 66.2 and 67.6 ppm, respectively, in the range expected for tricoordinate boron centers. In contrast, the bridging boron atoms correspond to sharp singlet signals at *δ*_B_ 3.4 (1^Cl^) and −3.9 ppm (1^Br^), attributed to the non-classical interaction between the electron-deficient boron atoms and electron-rich C=C double bonds. Treatment of the borole dimers 1^Cl^ and 1^Br^ with 2-butyne (4 equiv.) at elevated temperatures afforded the corresponding Diels–Alder products 2^Cl^ (99%) and 2^Br^ (98%). Compounds 2^Cl^ and 2^Br^ are liquids at room temperature and solidify^19^ at −35 °C. In their ^11^B{^1^H} NMR spectra, the bridging boron atoms correspond to sharp singlets at *δ*_B_ −7.5 (2^Cl^), and −11.3 ppm (2^Br^). Fagan *et al.* reported the synthesis and NMR spectra of compound 2^Ph^, but its crystal structure remained elusive.^9^ In this work, we present the crystal structure of 2^Ph^ in Fig. S52.†

### 2.2 Chloroborylene release from boranorbornadiene

**Scheme 2:**
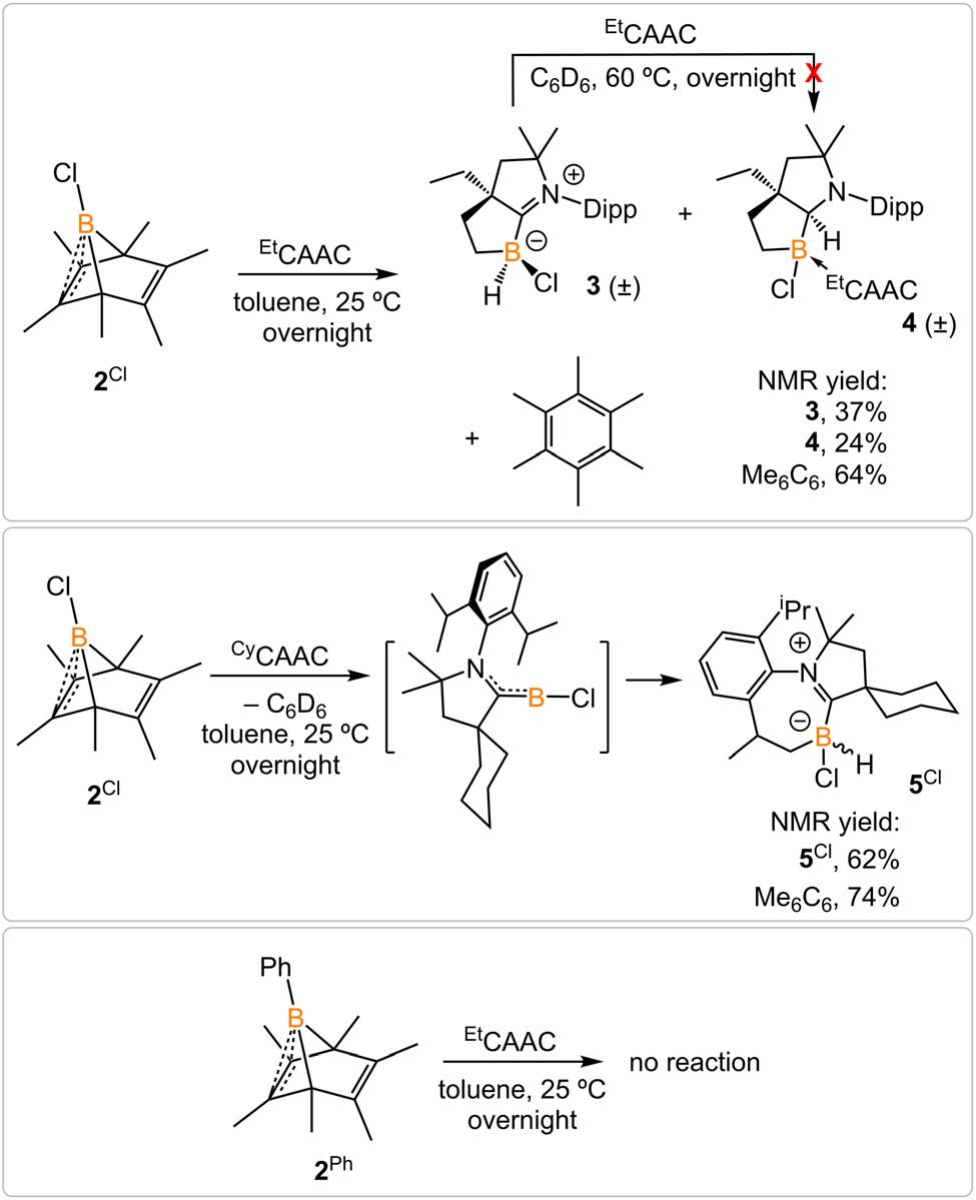
Treatment of 2^Cl^ with ^Et^CAAC and ^Cy^CAAC and attempt to generate phenylborylene.

**Fig. 2:**
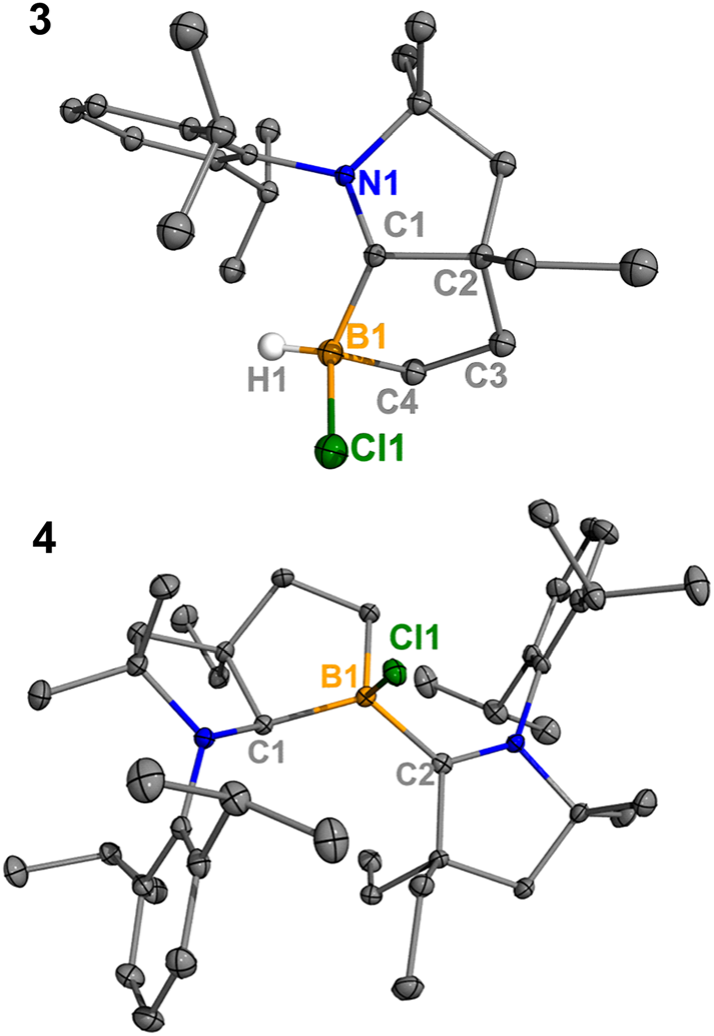
Molecular structures of 3 and 4 in the solid-state. Hydrogen atoms, except for the one connected to B1, have been omitted for clarity. Thermal ellipsoids are drawn at the 50% probability level.

Treatment of 2^Cl^ with cyclic(alkyl)(amino) carbene (^Et^CAAC) in toluene resulted in an immediate color change from colorless to orange (Scheme 2). The reaction led to the formation of several boron species based on ^11^B NMR spectroscopy. The ^1^H NMR spectrum revealed a significant amount of hexamethylbenzene (2.13 ppm in C_6_D_6_), suggesting the release of the “BCl” fragment from 2^Cl^. Two boron-containing species (3 and 4), along with hexamethylbenzene, crystallized out of the reaction mixture at −35 °C. Compound 3 displays a doublet at −10.9 ppm (^1^*J*_H–B_ = 86.0 Hz) and 4 displays a singlet at 2.9 ppm in its ^11^B{^1^H} NMR spectrum. After washing with hexanes to remove hexamethylbenzene, the remaining solids were recrystallized from toluene at −35 °C and compound 3 precipitated out first. Its ^11^B{^1^H} NMR spectrum only displayed a singlet at −10.9 ppm, indicative of a boron hydride moiety. Its ^13^C DEPT-135 (Distortionless Enhancement by Polarization Transfer) NMR spectrum displayed an inverted broad signal at 20.23 ppm (FWHM = 115.6 Hz), suggesting a methylene unit adjacent to the boron atom. Therefore, compound 3 was assigned as a CH_3_ activation product. Storage of a concentrated toluene solution of 3 at −35 °C afforded single crystals suitable for single-crystal X-ray diffraction (XRD) analysis. The solid-state structure of 3 reveals an intramolecular C–H bond activation of the ethyl group in ^Et^CAAC, a process that may occur *via* a transient dicoordinate chloroborylene (Fig. 2). Overall, the formation of 3 may be rationalized as a cascade reaction: the coordination of ^Et^CAAC to the boron atom, borylene release from hexamethylbenzene, and intramolecular C–H activation. The crystal structure of 4 was obtained by XRD by selecting 4 from a crystalline mixture of 3 and 4. Initially we hypothesized that 4 was generated from 3, in which a hydride (H1) migration from B1 to the electrophilic carbon C1 occurred followed by coordination of a second ^Et^CAAC to B1. However, heating compound 3 with ^Et^CAAC in C_6_D_6_ for one day did not result in any observable reaction. At this stage, the pathway leading to compound 4 remains unclear.

Treatment of 2^Cl^ with ^Cy^CAAC led to the formation of hexamethylbenzene and the C–H activation product 5^Cl^. Unlike 3, compound 5^Cl^ was obtained as two diastereomers (dr value = 67 : 33) with different ^11^B NMR chemical shifts (*δ* = −4.7, −8.0 ppm) and coupling constants (^1^*J*_B–H_ = 137.4 Hz, 67.6 Hz, respectively). The C–H activation products are quite similar to those generated from durylborylene (^Me^CAAC)(Dur)B: reported by Braunschweig *et al.*,^1*i*^ further implicating the formation of the proposed borylene intermediate in this type of reaction. Interestingly, in contrast to the B-Dur species, 3 and 5^Cl^ do not undergo a subsequent hydride migration from boron to carbon, an observation attributed to the stronger B–H bonds in 3 and 5^Cl^. Our attempt to generate a phenyl borylene using the same method was unsuccessful. The treatment of 2^Ph^ with ^Et^CAAC resulted in no reaction, likely a consequence of the steric hindrance of ^Et^CAAC and the phenyl group.

### 2.3 Mechanistic studies

**Fig. 3:**
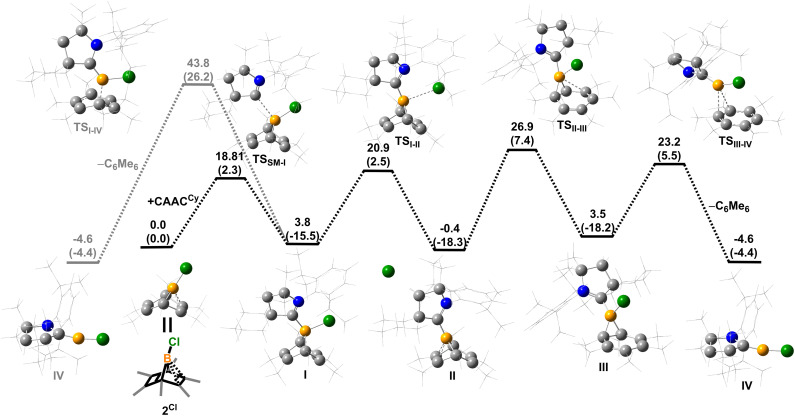
Energy profiles calculated for the reaction from SM to IV and from I to IV. The relative Gibbs free energies (calculated at 298 K) and electronic energies (in parentheses) are given in kcal mol^−1^ (in scale). (ωB97xD/6-311G**//ωB97M-V/def2-QZVPP level of theory).

Density functional theory (DFT) calculations were performed to provide further insight into the borylene-releasing process. All calculations were performed at the ωB97xD/6-311G** level of theory together with single point energy corrections at the ωB97M-V/def2-QZVPP level. According to the computational results, 2^Cl^ (SM) and ^Cy^CAAC first interact to form a Lewis acid–base adduct I (Fig. 3). While the chloroborylene (IV) could directly leave from hexamethylbenzene *via* transition state TS_I–IV_ in an exergonic step (Δ*G* = −8.1 kcal mol^−1^), its high activation barrier (43.8 kcal mol^−1^) is inconsistent with the experimental fact that the hexamethylbenzene extrusion process is complete at 25 °C (room temperature) within several hours. Alternatively, the B–C bond in I could undergo a 1,3-suprafacial-sigma shift, transforming from boranorbornadiene (I) to boranorcaradiene (III), followed by a borylene-releasing process. The barrier of the rate-determining step in this alternative stepwise pathway is predicted to be 27.3 kcal mol^−1^, a value surmountable at room temperature. Interestingly, in contrast to the per-phenyl-boranorbornadiene NHC adduct (*I*Me)(Ph)B(C_6_Ph_6_),^8,10^ which converts to its boranorcaradiene structure linked only by one transition state, the conversion from I to III is connected by two transition states (TS_I–II_ and TS_II–III_) and one intermediate (II), which is associated with a chloride dissociation and reassociation process. The intermediate II is considered to be a non-classical boronium species stabilized by a three-center two-electron bond arising from donation of the olefinic π bond to the Lewis-acidic boron center. Other possible reaction pathways from boranorcaradiene III to borylene IV are discussed in the ESI (Fig. S60†).

**Scheme 3:**
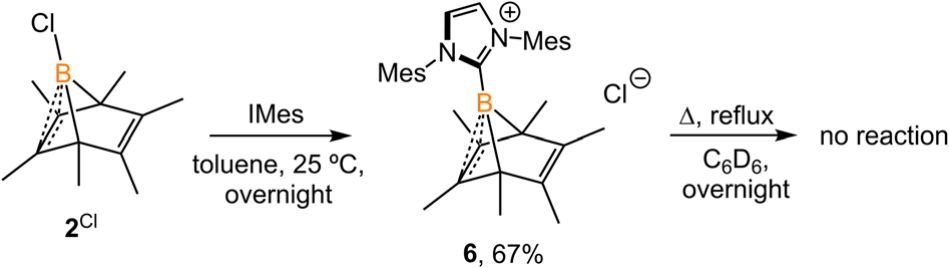
Synthesis of 6.

**Fig. 4:**
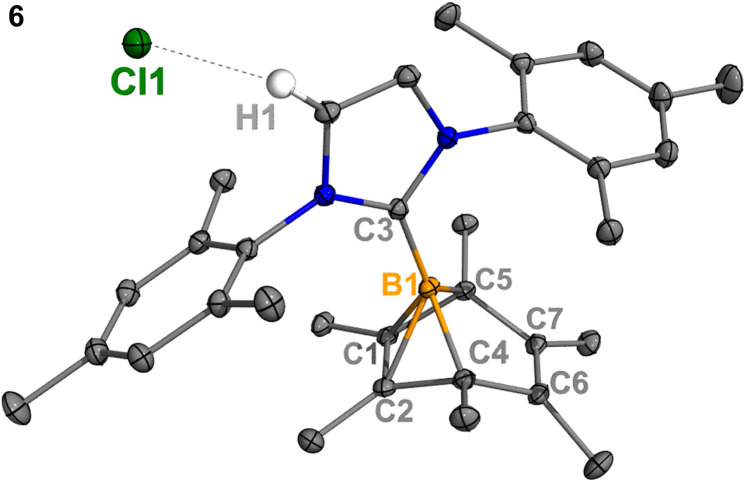
Molecular structure of 6 in the solid-state. Hydrogen atoms, except for H1, have been omitted for clarity. Thermal ellipsoids are drawn at the 50% probability level. Selected bond lengths [Å] and angles [°]: B1–C1 1.798(2), B1–C2 1.807(2), B1–C3 1.575(3), B1–C4 1.630(2), B1–C5 1.623(2), C1–C2 1.393(3), C6–C7 1.336(2), H1–Cl1 2.512, C4–B1–C5 96.78(9), C1–B1–C2 45.47, C4–B1–C5–C1 82.23.

Experimentally, all attempts to isolate intermediates between I and IV were unsuccessful, presumably because of the spontaneity of the borylene-releasing process. We proposed that using an NHC ligand instead of a CAAC ligand would render the borylene IV less stable,^2*b*,11^ and the releasing process less favorable, thereby enabling us to isolate key intermediates. Leaving a 1 : 1 mixture of 2^Cl^ and *I*Mes (Mes = 2,4,6-trimethylphenyl) undisturbed overnight (Scheme 3) resulted in the precipitation of white crystalline solids. XRD analysis revealed a structure of the boranorbornadiene cation 6 (Fig. 4), which is analogous to the putative intermediate II. The B1–C1 (1.806(2) Å) and B1–C2 (1.798(2) Å) distances in 6 are shorter than those in 2^Ph^ (1.816(2) Å and 1.811(2) Å, respectively, Fig. S52†), and the C1–C2 (1.393(2) Å) bond in 6 is longer than that in 2^Ph^ (1.388(2) Å), indicating a stronger interaction between the boron atom and C=C double bond. The center boron atom gave a single sharp resonance at −16.4 ppm in the ^11^B NMR spectrum. The formation of compound 6 corroborates our proposed mechanism and is consistent with the calculated energies [I (3.8 kcal mol^−1^), II (−0.4 kcal mol^−1^), III (3.5 kcal mol^−1^)], suggesting that II is the most stable isomer. However, 6 is even stable in boiling C_6_D_6_ for several hours, indicating that the borylene-releasing process in this case is extremely unfavorable.

### 2.4 Bromoborylene release from boranorbornadiene

**Scheme 4:**
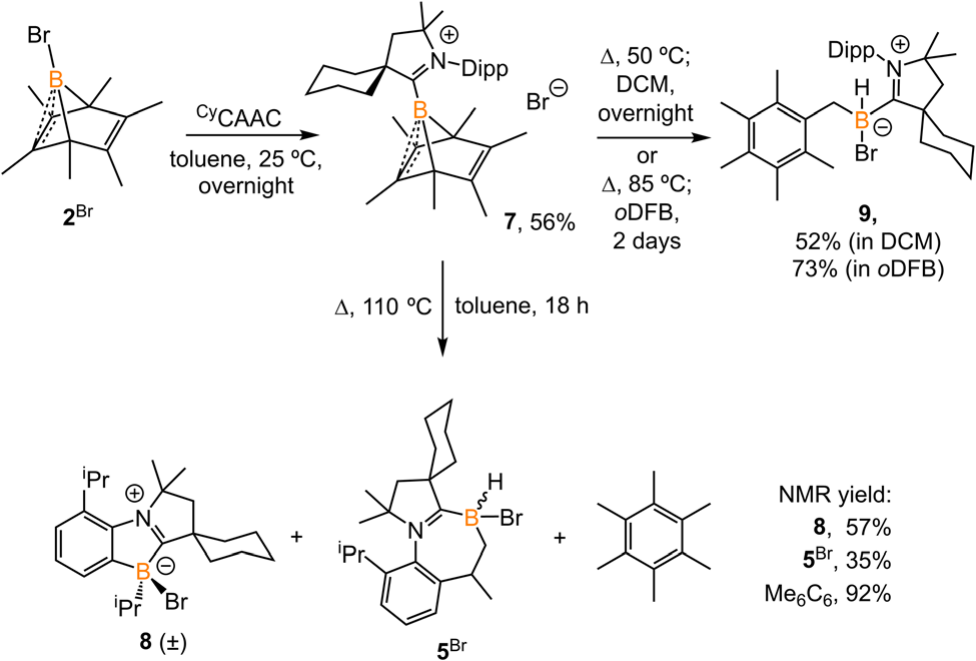
Synthesis of 7 and its reaction at elevated temperature in different solvents. (*o*DFB = *ortho*-difluorobenzene).

**Fig. 5:**
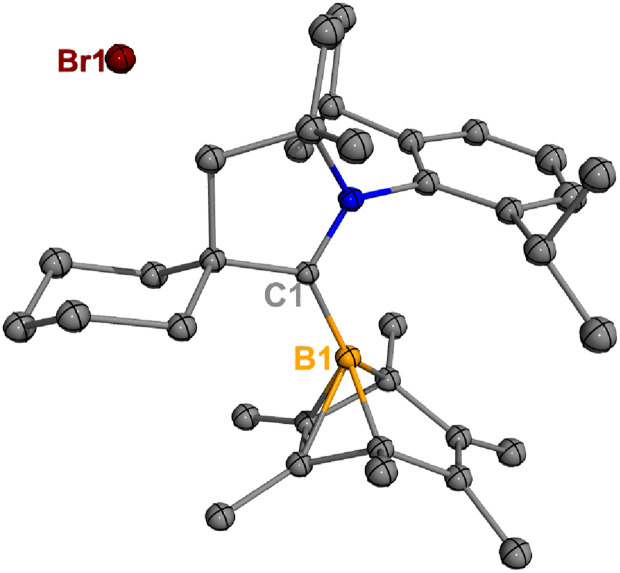
Molecular structure of 7 in the solid state. Hydrogen atoms have been omitted for clarity. Thermal ellipsoids are drawn at the 50% probability level.

**Scheme 5:**
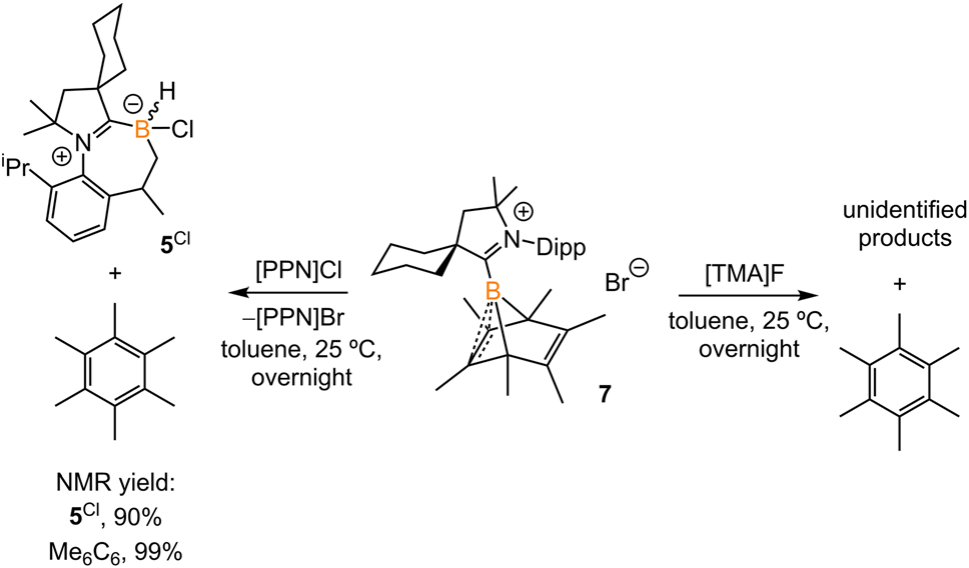
Treatment of 7 with [PPN]Cl and [TMA]F.

Treatment of 2^Br^ with ^Cy^CAAC in toluene (Scheme 4) resulted in the precipitation of yellow crystalline solids (7). The ^11^B NMR spectrum of 7 features a resonance at *δ*_B_ −13.3 ppm. XRD analysis revealed a boronium structure analogous to that of 6 (Fig. 5). Compound 7 is nearly insoluble in toluene and does not release borylene at 25 °C. However, heating a suspension of 7 in toluene at 100 °C overnight afforded a mixture of hexamethylbenzene, an intramolecular C–H activation product 5^Br^, and a C–C activation product 8, indicating that the borylene-releasing process occurred at an elevated temperature. Interestingly, the C–C activation product was not produced in the reaction of 2^Cl^ with ^Cy^CAAC (Scheme 2). Based on the studies conducted by Braunschweig^11,12^ and Lin *et al.*,^12^ the formation of 8 arises due to the enhanced Lewis acidity of CAAC-stabilized bromoborylene compared to chloroborylene. In the C(sp^2^)–C(sp^3^) activation process, bromoborylene may lower the barrier of the rate-determining π-coordination step, making the C(sp^2^)–C(sp^3^) bond activation more favorable. Treatment of 7 with bis(triphenylphosphine)iminium chloride ([PPN]Cl) in toluene at 25 °C also resulted in hexamethylbenzene extrusion and the formation of 5^Cl^ (Scheme 5). Although we attempted to generate the CAAC-stabilized fluoroborylene in a similar manner, the reaction yielded intractable mixtures containing hexamethylbenzene and unidentified boron-containing species.

**Fig. 6:**
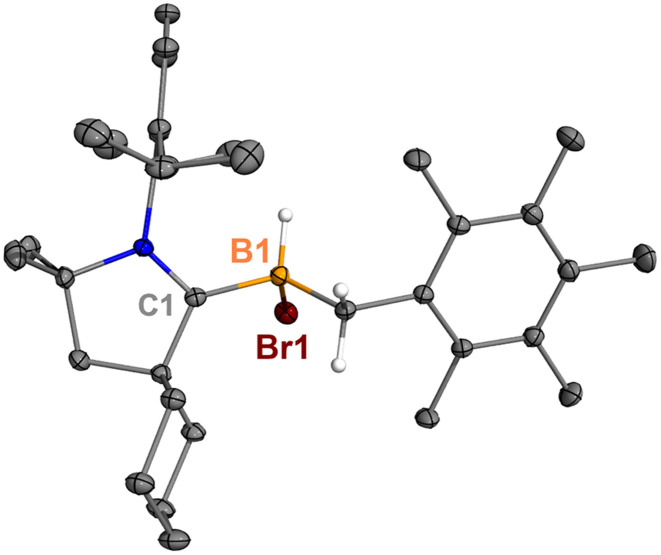
Molecular structure of 9 in the solid state. Hydrogen atoms, except for the one connected to B1, have been omitted for clarity. Thermal ellipsoids are drawn at the 50% probability level.

Surprisingly, heating 7 in polar solvents like DCM or *o*-difluorobenzene (Scheme 4) led to the formation of a single boron species (9) displaying a broad singlet at *δ*_B_ −8.0 ppm, with no evidence of hexamethylbenzene formation *via*^1^H NMR spectroscopy. Single crystals suitable for XRD analysis were grown by slow diffusion of hexanes into a concentrated DCM solution of 7. The structure of 9 corresponds to the product of a process in which the B1 atom inserts into a C–H bond of the methyl group in hexamethylbenzene (Fig. 6). This reaction is relatively clean, and no other side products, including 5^Br^ and 8, were observed in significant amounts, suggesting that the C–H activation process may not proceed through a borylene intermediate.

**Fig. 7:**
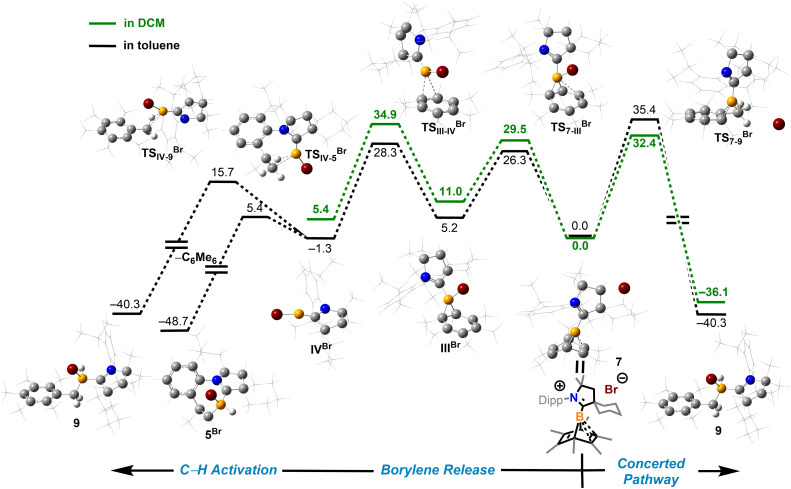
Energy profiles calculated for the reaction from 7 to 9 and from 7 to V^Br^ (or VI^Br^). The relative Gibbs free energies (calculated at 298 K) are given in kcal mol^−1^ (in scale). The calculation applied solvation models in toluene (path in black and gray) and DCM (path in green). (ωB97xD/6-311G**//ωB97M-V/def2-QZVPP level of theory).

To understand how solvents affected the reaction outcome, DFT calculations at the same level of theory (ωB97M-V/def2-QZVPP//ωB97xD/6-311G**) were performed (Fig. 7). While applying the dichloromethane solvation model (path in green), the barrier of the borylene-release process was elevated to 34.9 kcal mol^−1^, 7.6 kcal mol^−1^ higher than the chloroborylene-releasing process in toluene (Fig. 3). Alternatively, the reaction pathway leading to the formation of 9*via* transition state TS_7–9_^Br^ has a lower energy barrier (32.4 kcal mol^−1^). The optimized structure of TS_7–9_^Br^ features an agostic interaction between the boron center and the C–H bond. Therefore, the formation of 9 involves an intramolecular concerted process where (CAAC)B^+^ reductively eliminates from hexamethylbenzene and oxidatively inserts into the C–H bond. The pathway in black was calculated while applying the toluene solvation model. In toluene, the energy barrier from 7 to 9*via*TS_7–9_^Br^ increased to 35.4 kcal mol^−1^. In comparison, the energy barrier of the borylene-release process (from 7 to IV^Br^) decreased to 28.3 kcal mol^−1^ and thus became the more favorable process. Compared to the intermolecular C–H activation of hexamethylbenzene, the irreversible intramolecular C–H activation of the diisopropylphenyl (Dipp) group is more favorable, finally leading to the formation of compound 5^Br^. Overall, the computational results are consistent with our experimental observations.

### 2.5 Boron-centered Büchner reaction

**Scheme 6:**
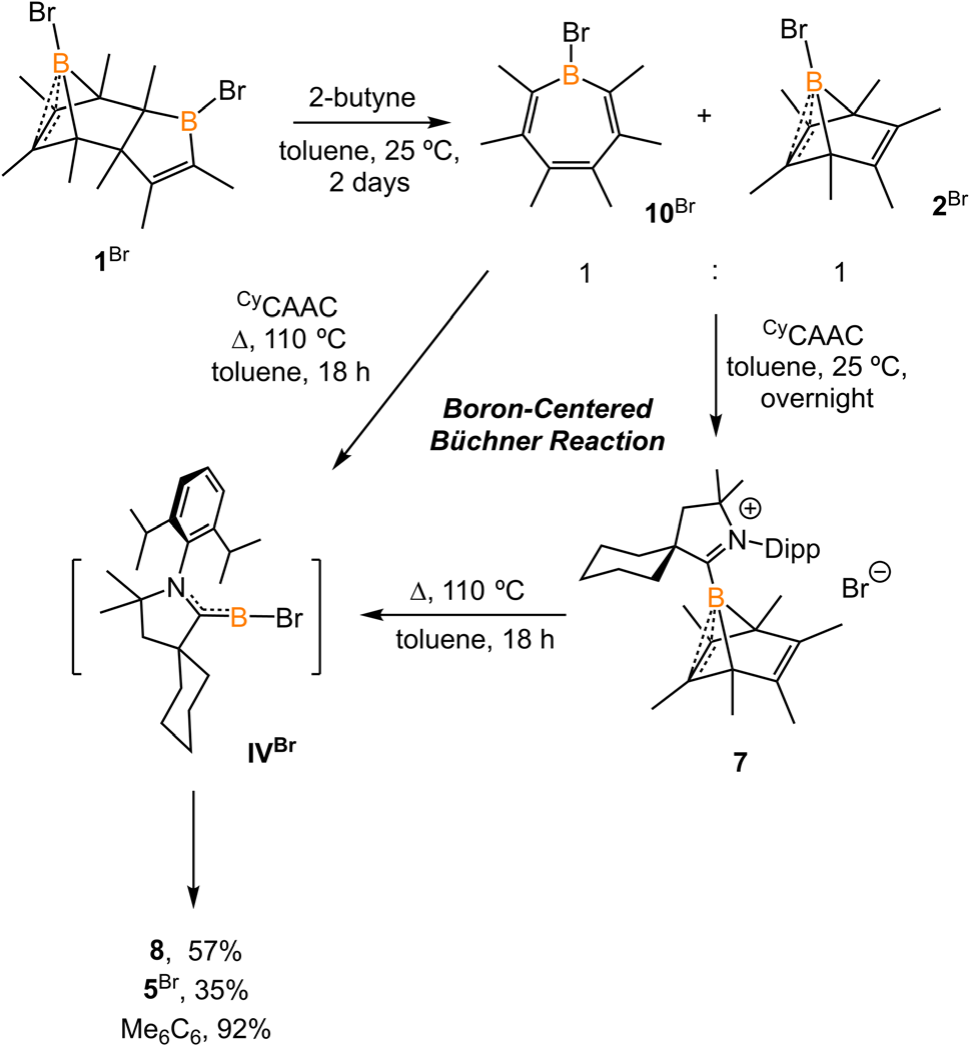
Synthesis of 10^Br^ and its conversion to 7.

The reaction of 1^Br^ with an excess of 2-butyne (10 equiv.) at 25 °C for two days afforded a 1 : 1 mixture of bromoborepin compound 10^Br^ and boranorbornadiene compound 2^Br^ (Scheme 6). The boron atom in the borepin evinces a broad singlet signal at 57.9 ppm in its ^11^B NMR spectrum. Compound 10^Br^ fully converts to 2^Br^ at an elevated temperature and proved to be the key intermediate in the transformation of 1^Br^ to 2^Br^.^13^ Compound 10^Br^ was challenging to separate from 2^Br^. Treatment of the mixture of 10^Br^ and 2^Br^ with ^Cy^CAAC in toluene overnight resulted in the precipitation of yellow crystalline solids. All components in the reaction mixture displayed only one dominant signal at −13.3 ppm in the ^11^B NMR spectrum, attributed to compound 7. This assignment is supported by ^1^H NMR spectroscopy data. Therefore, the *in situ* formed Lewis acid–base adduct ^Cy^CAAC·10^Br^ converts to 7 at 25 °C. Indeed, a similar borepin to boranorcaradiene transformation was observed by Taniguchi *et al.*^14^ In addition, compound 7 extrudes hexamethylbenzene upon heating in a non-polar solvent. Therefore, the transformation from ^Cy^CAAC·10^Br^ to borylene IV^Br^ is formally a boron-centered retro Büchner-ring-expansion reaction. It should be noted that other main-group element-centered Büchner reactions, including those for carbon,^7*b*,15^ silicon,^7*a*,16^ aluminum,^17^ and phosphorus,^18^ have been reported. The present work similarly demonstrates an example of a boron-centered retro Büchner reaction and its feasibility for releasing subvalent boron species.

## 3 Conclusions

In conclusion, we have synthesized chloro-boranorbornadiene and bromo-boranorbornadiene XB(C_6_Me_6_) (X = Cl and Br). The coordination of CAAC to XB(C_6_Me_6_) promotes hexamethylbenzene extrusion and the release of CAAC-ligated halogen–borylene (X = Cl, room temperature; X = Br, elevated temperature). The experimental and computational data suggest that the borylene-releasing process goes through a boranorbornadiene cation intermediate and a boranorcaradiene intermediate. Depending on the CAAC ligand and the substituents on boron, the subsequent transformations of di-coordinate borylene varied, producing different C–H and C–C activation products. CAAC ligands provide a better stabilization effect compared to NHC ligands, making the borylene-releasing process more kinetically and thermodynamically favorable. The release of borylene from hexamethylbenzene is the first example of a boron-centered arene extrusion reaction and the first example of thermal reductive elimination taking place at a single boron atom. These reactions have laid the groundwork for unusual borylene transfer chemistry and further studies remain ongoing in our laboratories.

## Author contributions

C. Z. conceptualized the project, synthesized and characterized the compounds, performed the computational work, and drafted the manuscript. R. J. G. and C. C. C. supervised the project, acquired financial support for the project, and revised the manuscript.

## Conflicts of interest

There are no conflicts to declare.

## Supplementary Material

SC-015-D4SC05524A-s001

SC-015-D4SC05524A-s002

SC-015-D4SC05524A-s003

## Data Availability

Experimental details, characterization, and computational details, including Fig. S1–S60 and Tables S1–S15,† X-ray crystallographic data for 1^Cl^, 2^Ph^, 3, 4, 5^Cl^, 5^Br^, 6, 7, 8, and 9 (CIF). CCDC identification codes 297451–2297460 are associated with the supplementary crystallographic data for this paper.
