# Supplementary material for: Arene extrusion as an approach to reductive elimination at boron: implication of carbene-ligated haloborylene as a transient reactive intermediate
Source: Chem Sci. 2024 Oct 3;15(43):17873–80. doi: 10.1039/d4sc05524a (PMC11472773; doi:10.1039/d4sc05524a)
Supplement: SC-015-D4SC05524A-s001 [file SC-015-D4SC05524A-s001.pdf]

Supplementary Information (SI) for Chemical Science.
This journal is © The Royal Society of Chemistry 2024

Supporting Information

Arene extrusion as an approach to reductive elimination at boron: Implication of carbene-ligated haloborylene as a transient reactive intermediate

Chonghe Zhang, Robert J. Gilliard Jr.*, and Christopher C. Cummins*

Department of Chemistry, Massachusetts Institute of Technology, Cambridge, Massachusetts 02139, United States

Supporting Information Contents:

Synthetic details and characterization data **S2**
General considerations S2
Synthesis of **1**^{Cl} S2
Synthesis of **1**^{Br} S4
Synthesis of **2**^{Cl} S6
Synthesis of **2**^{Br} S8
Synthesis of **2**^{Ph} S10
Synthesis of **6** S10
Synthesis of **7** S12
The reaction of **2**^{Cl} with ^{Et}CAAC S14
The reaction of **2**^{Cl} with ^{Cy}CAAC S21
Heating **7** in toluene S25
Heating **7** in DCM (or *o*-difluorobenzene) S31
Synthesis of **10**^{Cl} (mixed with **2**^{Cl}) S35
Synthesis of **10**^{Br} (mixed with **2**^{Br}) S37

Crystal structure **S40**

Computational Studies **S51**

Discussion on other possible mechanism **S56**

Reference **S57**

Synthetic details and characterization data

General considerations: all manipulations were performed in a Vacuum Atmospheres model MO-40M glovebox under an inert atmosphere of purified N_2 or using standard Schlenk techniques. All solvents were obtained anhydrous and oxygen-free by bubble degassing (argon) and purified by passing through columns of alumina and Q5.[1] Once collected, solvents were stored over activated 4 Å molecular sieves (20 wt%) inside the glovebox.[2] All glassware was oven-dried for at least 6 h prior to use, at temperatures greater than 150 ºC. Deuterated solvents were purchased from Cambridge Isotope Labs and were degassed three times by the freeze-pump-thaw method and stored over activated 4 Å molecular sieves for 48 h in the glovebox prior to use. 4 Å molecular sieves (Millipore-Sigma) were dried by heating at 200 ºC under dynamic vacuum for at least 48 h prior to use.

$Cp_2Zr(C_4Me_4)$,[3] $(PhB(C_4Me_4))_2$,[4] ^{Et}CAAC,[5, 6] ^{Cy}CAAC,[5, 6] and IMes,[7] were prepared according to literature procedures. 2-Butyne was purchased from Millipore-Sigma and degassed by the freeze-pump-thaw method and stored over activated 4 Å molecular sieves for 48 h in the glovebox prior to use. Bis(triphenylphosphine)iminium chloride ([PPN]Cl) and tetramethylammonium fluoride ([TMA]F) were purchased from Millipore-Sigma and dried by heating at 120 ºC under a dynamic vacuum for at least 6 h prior to use. Unless otherwise noted, all other chemicals were purchased commercially and used as received.

NMR spectra were obtained on Bruker Avance 400, Avance 401, Neo 402, Neo 500, and Neo 501 spectrometers. ^{1}H and ^{13}C NMR spectra were referenced to residual CD_2Cl_2 (^{1}H = 5.32 ppm, ^{13}C = 54.0 ppm) or C_6D_6 (^{1}H = 7.16 ppm, ^{13}C = 128.06 ppm). HRMS were obtained on high-resolution JEOL AccuTOF 4G LC-plus equipped with an ionSense DART (Direct Analysis in Real Time) source. Elemental analyses were obtained on Thermo Scientific™ Flash*Smart*™ Elemental Analyzers. Samples were removed from the glovebox in sealed vials and briefly handled in air prior to data collection.

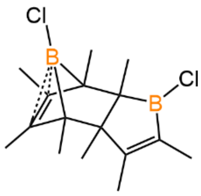

Synthesis of 1^{Cl}: to a pentane (250 mL) suspension of $Cp_2Zr(C_4Me_4)$ (10.0 g, 30.3 mmol, 1 equiv), BCl_3 (1M in hexanes, 32 mL, 32 mmol, 1.06 equiv) was added dropwise at 25 ºC. The color of the suspension immediately changed from red to brown. The suspension was stirred overnight at 25 ºC and then filtered. The filtrate was concentrated to approx. 15 mL to obtain a dark brown solution. The solution was cooled at –35 ºC and the product precipitated as brown crystalline solids (4.3 g, 92%).

^{1}H NMR (500 MHz, C_6D_6): 0.94 (s, 3H, C<u>H</u>$_3$), 1.00 (s, 3H, C<u>H</u>$_3$), 1.18 (s, 3H, C<u>H</u>$_3$), 1.28 (s, 3H, C<u>H</u>$_3$), 1.39 (s, 3H, C<u>H</u>$_3$), 1.45 (s, 3H, C<u>H</u>$_3$), 1.50 (s, 3H, C<u>H</u>$_3$), 1.54 (s, 3H, C<u>H</u>$_3$). **^{11}B NMR** (161 MHz, C_6D_6): 3.38 (s, <u>B</u>–C(CH$_3$)–C(CH$_3$)=C), 66.23 (br. s, <u>B</u>–C(CH$_3$)=C(CH$_3$)–C). **^{13}C{^{1}H} NMR** (126 MHz, C_6D_6): 11.23 (s, <u>C</u>H$_3$), 11.44 (s, <u>C</u>H$_3$), 11.75 (s, <u>C</u>H$_3$), 11.98 (s, <u>C</u>H$_3$), 14.99 (s, <u>C</u>H$_3$), 15.13 (s, <u>C</u>H$_3$), 18.08 (s, <u>C</u>H$_3$), 52.34 (br. s, B–C–<u>C</u>H$_3$), 53.10 (br. s,

B–C–<u>C</u>H3), 66.48 (s, B–C(CH3)=C(CH3)–<u>C</u>), 129.49 (s, B–C(CH3)–<u>C</u>(CH3)=C), 132.20 (s, B–C(CH3)–C(CH3)=<u>C</u>), 183.62 (s, B–C(CH3)=<u>C</u>(CH3)–C). **HRMS** (m/z): $[M+H]^+$ calcd. for $C_{16}H_{25}B_2Cl_2$, 309.15194; found 309.15171. **Elemental analysis**: calcd. for $C_{16}H_{24}B_2Cl_2$, C, 62.22; H, 7.83; found: C, 61.73; H, 7.89.

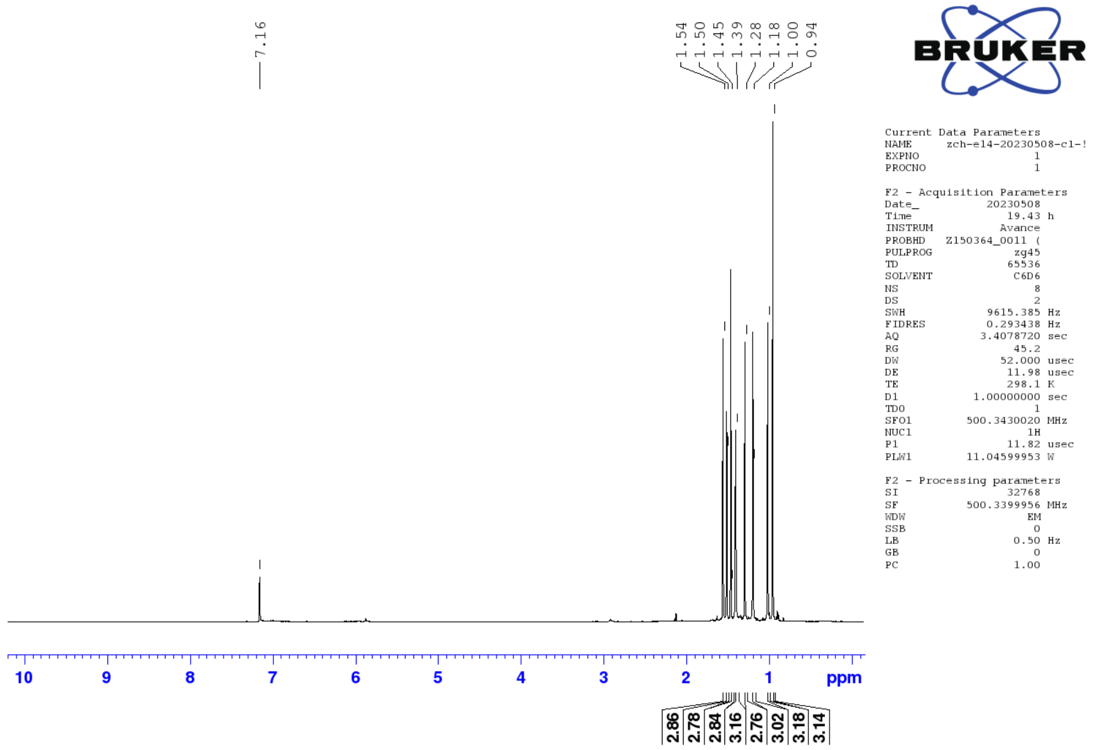

Figure S1. ^1H NMR spectrum of $\mathbf{1}^{Cl}$ in C_6D_6

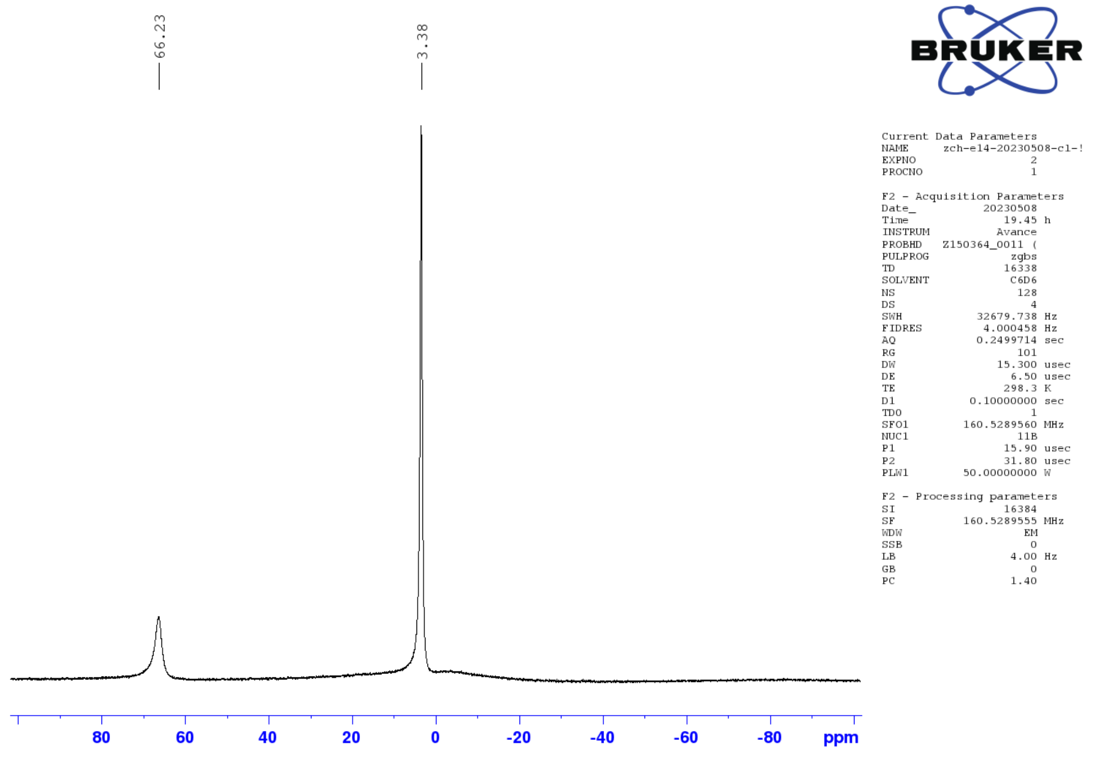

Figure S2. ^{11}B NMR spectrum of $\mathbf{1}^{Cl}$ in C_6D_6

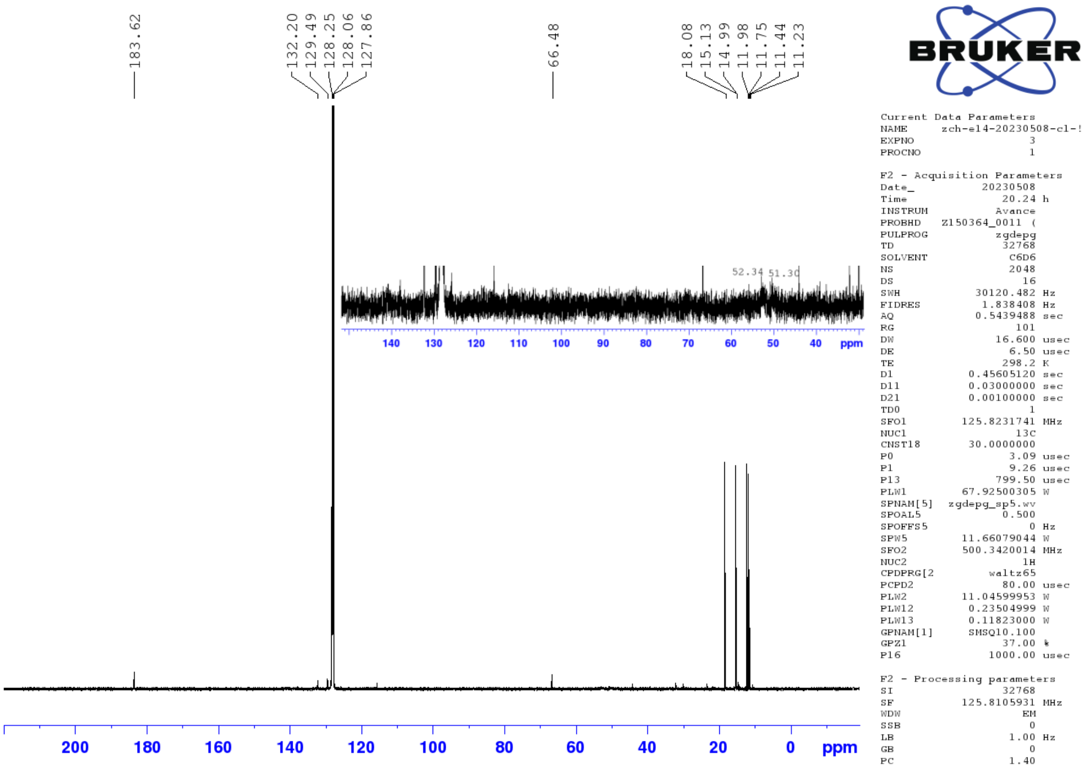

Figure S3. $^{13}C\{^{1}H\}$ NMR spectrum of $\mathbf{1}^{Cl}$ in C_6D_6

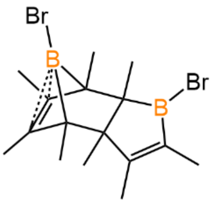

Synthesis of $\mathbf{1}^{Br}$: to a pentane (100 mL) suspension of $Cp_2Zr(C_4Me_4)$ (1.71g, 5.2 mmol, 1.0 equiv), BBr_3 (1.37 g, 5.46 mmol, 1.05 equiv) was added dropwise at 25 °C. The color of the suspension immediately changed from red to light brown. The suspension was stirred overnight at 25 °C and then filtered. All volatiles in the red-brown filtrate were removed under reduced pressure to obtain a brown solid (872 mg, 84%). The purity of the crude product was acceptable based on the NMR spectra.

^{1}H NMR (500 MHz, C_6D_6): 0.93 (s, 3H, C<u>H</u>$_3$), 1.02 (s, 3H, C<u>H</u>$_3$), 1.17 (s, 3H, C<u>H</u>$_3$), 1.25 (s, 3H, C<u>H</u>$_3$), 1.42 (s, 3H, C<u>H</u>$_3$), 1.43 (s, 3H, C<u>H</u>$_3$), 1.44 (s, 3H, C<u>H</u>$_3$), 1.59 (s, 3H, C<u>H</u>$_3$); **^{11}B NMR** (161 MHz, C_6D_6): –3.90 (s, <u>B</u>–C(CH$_3$)–C(CH$_3$)=C), 67.62 (br. s, <u>B</u>–C(CH$_3$)=C(CH$_3$)–C); **^{13}C{^{1}H} NMR** (126 MHz, C_6D_6): 11.96 (s, <u>C</u>H$_3$), 12.12 (s, <u>C</u>H$_3$), 12.37 (s, <u>C</u>H$_3$), 12.57 (s, <u>C</u>H$_3$), 12.84 (s, <u>C</u>H$_3$), 15.32 (s, <u>C</u>H$_3$), 15.45 (s, <u>C</u>H$_3$), 18.20 (s, <u>C</u>H$_3$), 51.25 (br. s, B–C–<u>C</u>H$_3$), 54.36 (br. s, B–C–<u>C</u>H$_3$), 68.02 (s, B–C(CH$_3$)=C(CH$_3$)–<u>C</u>), 130.85 (s, B–C(CH$_3$)–C(CH$_3$)=<u>C</u>), 142.47 (br. s, B–C–<u>C</u>H$_3$), 183.99 (s, B–C(CH$_3$)=<u>C</u>(CH$_3$)–C). **HRMS** (m/z): $[M+H]^+$ calcd. for $C_{16}H_{25}B_2Br_2$, 397.05091; found 397.05166. **Elemental analysis**: calcd. for $C_{16}H_{24}B_2Br_2$, C, 48.31; H, 6.08; found: C, 48.11; H, 5.95.

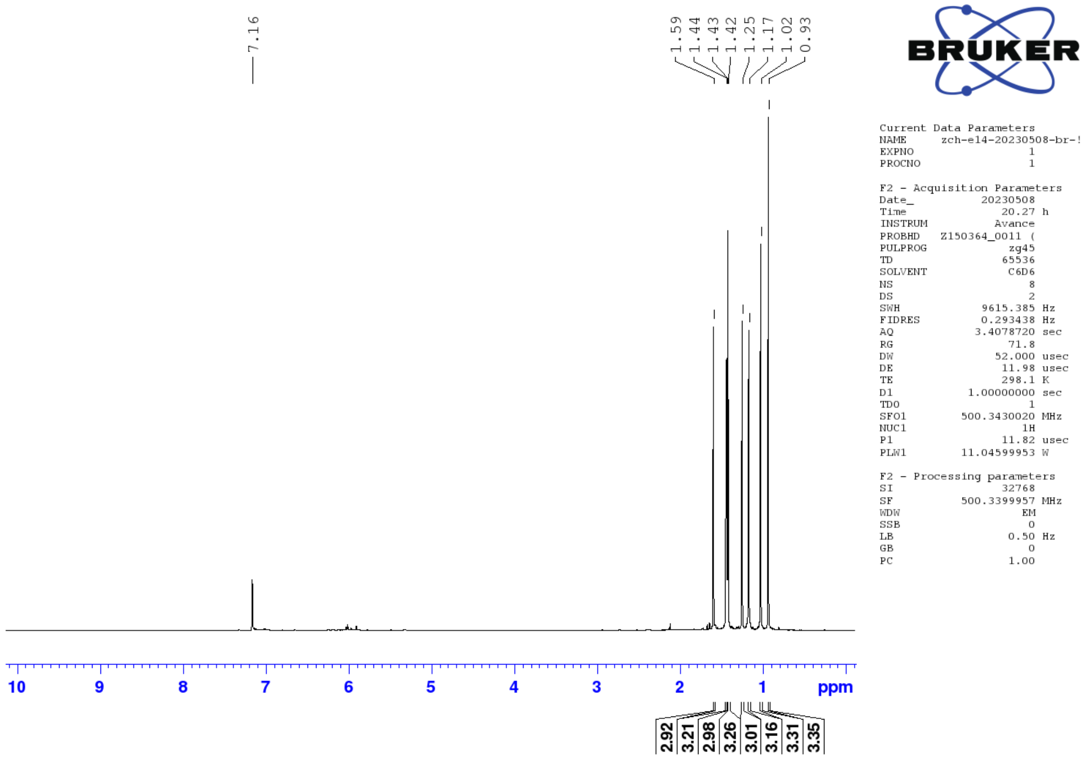

Figure S4. ^{1}H NMR spectrum of **1**^{Br} in C_6D_6

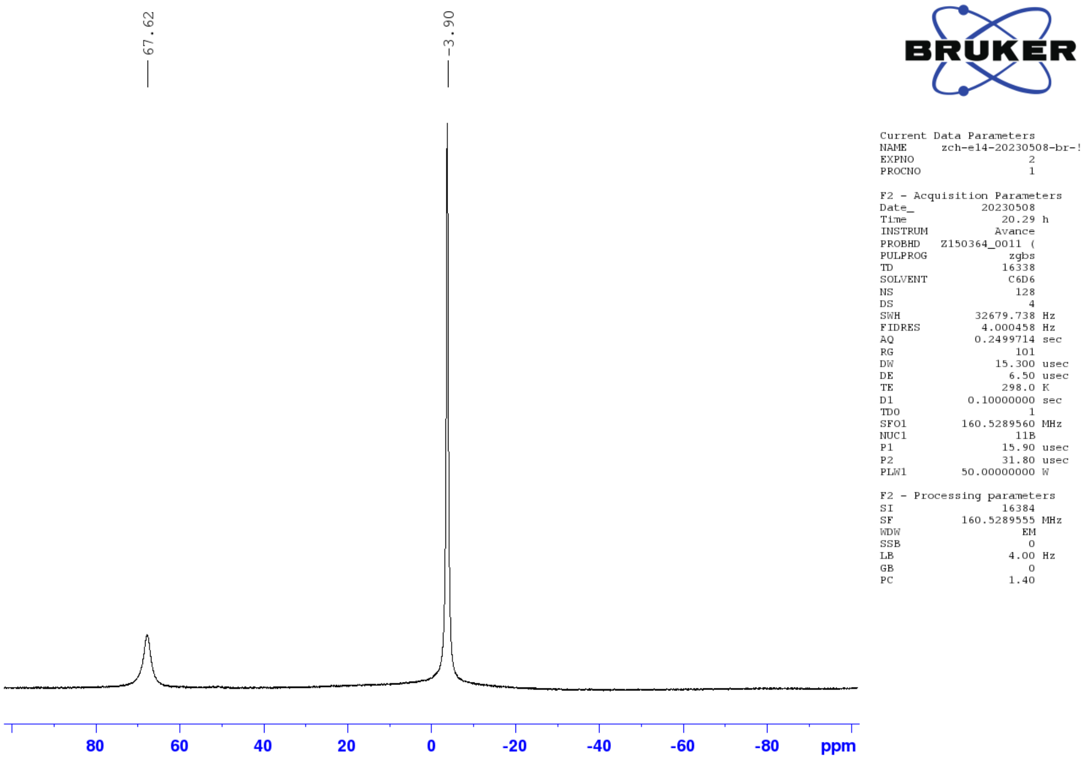

Figure S5. ^{11}B NMR spectrum of **1**^{Br} in C_6D_6

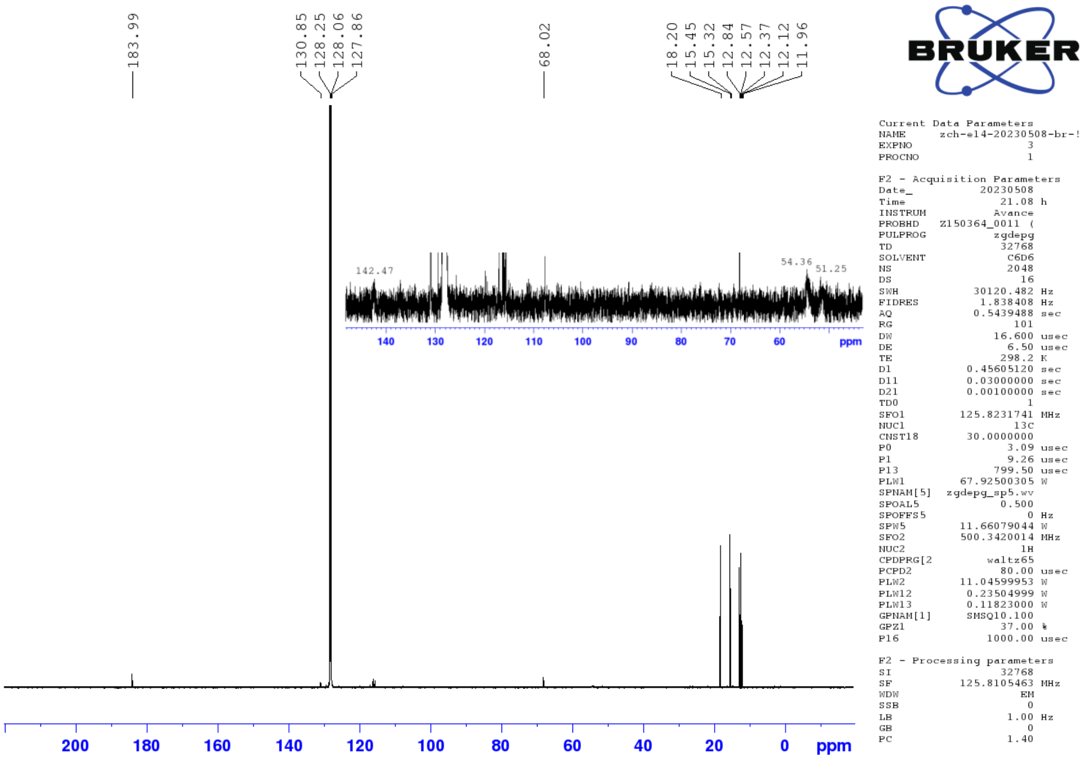

Figure S6. $^{13}C\{^1H\}$ NMR spectrum of **1**^{Br} in C_6D_6

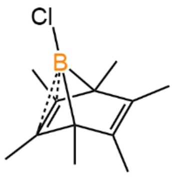

Synthesis of 2^{Cl}: inside the glovebox, compound **1**^{Cl} (730 mg, 2.36 mmol, 1.0 equiv) and 2-butyne (510 mg, 9.44 mmol, 4.0 equiv) were dissolved in toluene (7 mL) in a Schlenk flask. The flask was taken out of the glovebox and heated at 55 °C overnight to obtain an orange solution. The flask was then connected to a dynamic vacuum for approx. 10 mins to remove the remaining 2-butyne. The solution was heated at 70 °C for another 6 hours. All volatiles in the solution were removed under vacuum to obtain an orange oil (980 mg, 99%). The purity of the crude product was acceptable based on the NMR spectra.

^{1}H NMR (500 MHz, C_6D_6): 1.41 (s, 6H, C<u>H</u>$_3$), 1.52 (s, 12H, C<u>H</u>$_3$); **^{11}B NMR** (161 MHz, C_6D_6): –7.50 (s); **$^{13}C\{^1H\}$ NMR** (126 MHz, C_6D_6): 11.11 (s, <u>C</u>H$_3$), 11.64 (s, <u>C</u>H$_3$), 60.11 (br. s, B–<u>C</u>–CH$_3$), 126.95 (s, <u>C</u>=<u>C</u>). **HRMS** (m/z): [M+H]$^+$ calcd. for $C_{12}H_{19}BCl$, 209.12683; found 209.12579. **Elemental analysis**: calcd. for $C_{12}H_{18}BCl$, C, 69.12; H, 8.70; found: C, 69.42; H, 8.75.

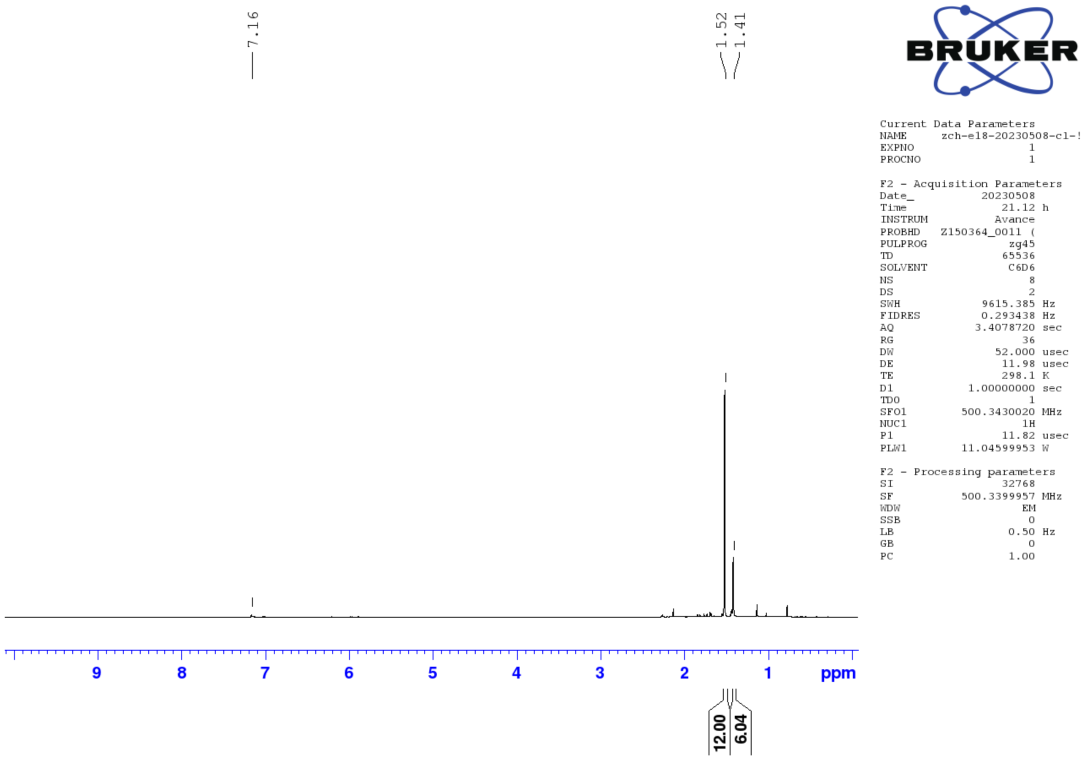

Figure S7. ^{1}H NMR spectrum of **2**^{Cl} in C_6D_6

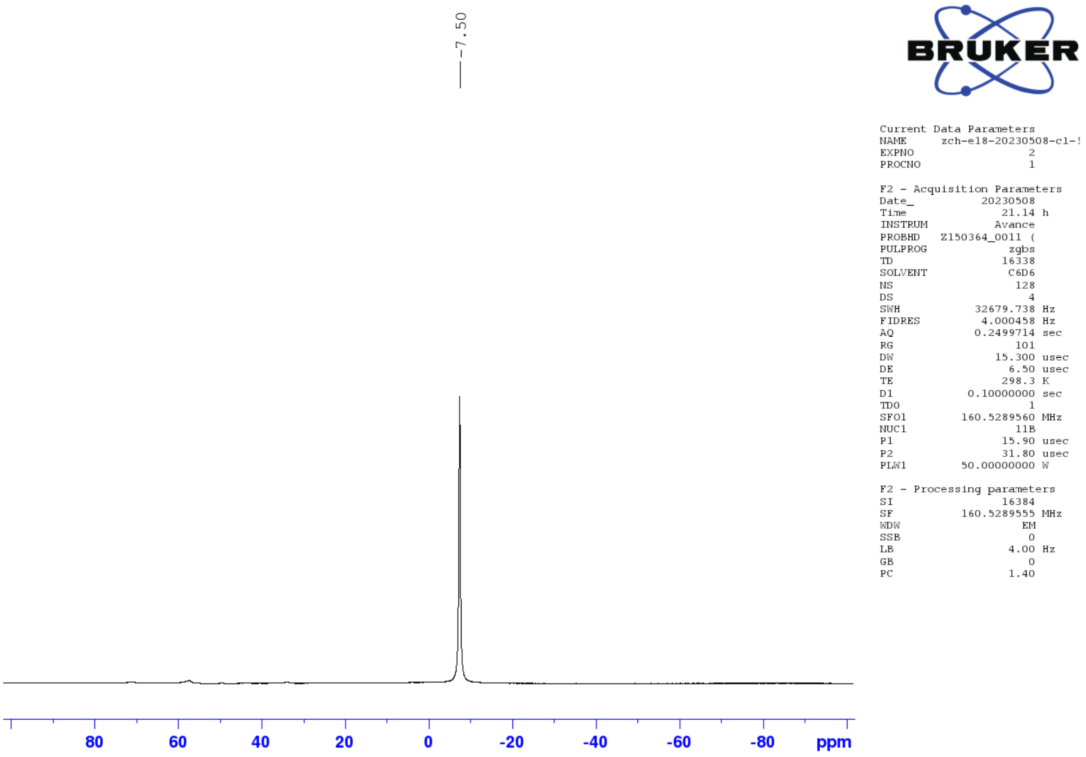

Figure S8. ^{11}B NMR spectrum of **2**^{Cl} in C_6D_6

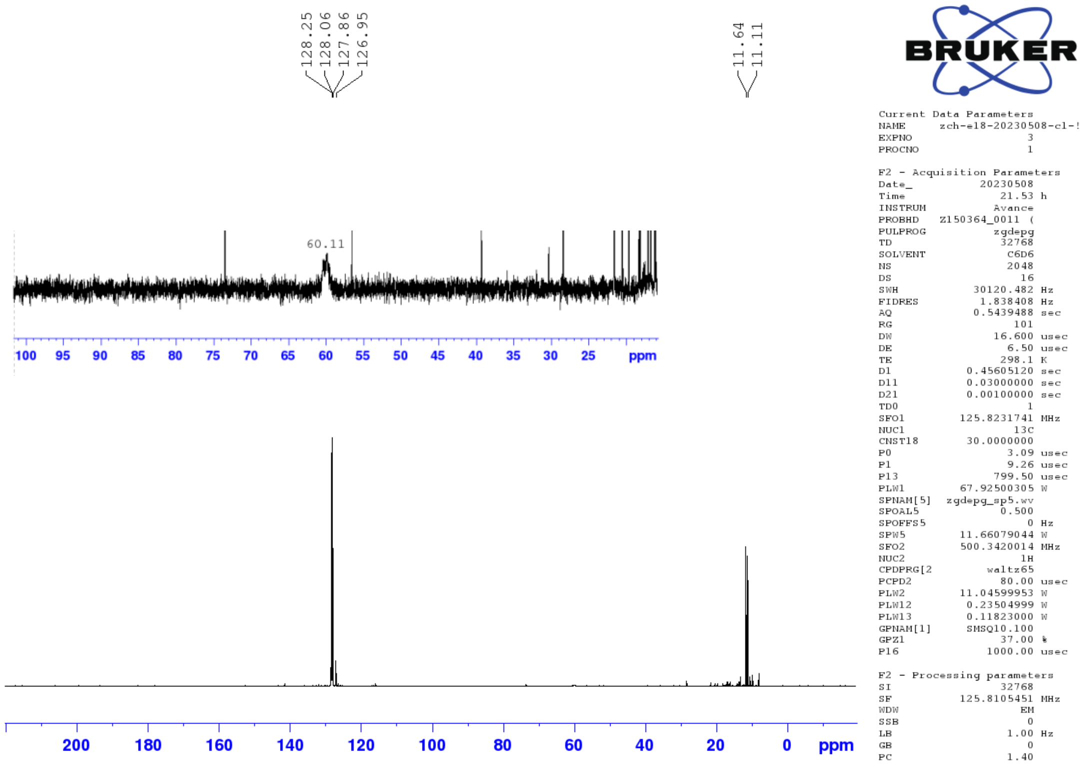

Figure S9. $^{13}C\{^1H\}$ NMR spectrum of **2**^{Cl} in C_6D_6

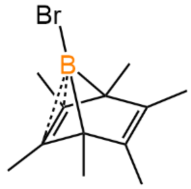

Synthesis of 2^{Br}: inside the glovebox, compound **1**^{Br} (730 mg, 1.83 mmol, 1.0 equiv) and 2-butyne (395 mg, 7.32 mmol, 4.0 equiv) were dissolved in toluene (11 mL) in a Schlenk flask. The flask was taken out of the glovebox and heated at 60 ºC for one day to obtain an orange solution. All volatiles in the solution were removed under vacuum to obtain an orange oil (907 mg, 98%). The purity of the crude product was acceptable based on the NMR spectra.

^{1}H NMR (500 MHz, C_6D_6): 1.40 (s, 6H, C<u>H</u>$_3$), 1.50 (s, 12H, C<u>H</u>$_3$); **^{11}B NMR** (161 MHz, C_6D_6): –11.30 (s); **^{13}C{^{1}H} NMR** (126 MHz, C_6D_6): 11.84 (s, <u>C</u>H$_3$), 11.99 (s, <u>C</u>H$_3$), 60.77 (br. s, B–<u>C</u>–CH$_3$), 126.44 (s, <u>C</u>=<u>C</u>). **HRMS** (m/z): not detected. **Elemental analysis**: calcd. for $C_{12}H_{18}BBr$, C, 56.97; H, 7.17; found: C, 56.33; H, 7.13.

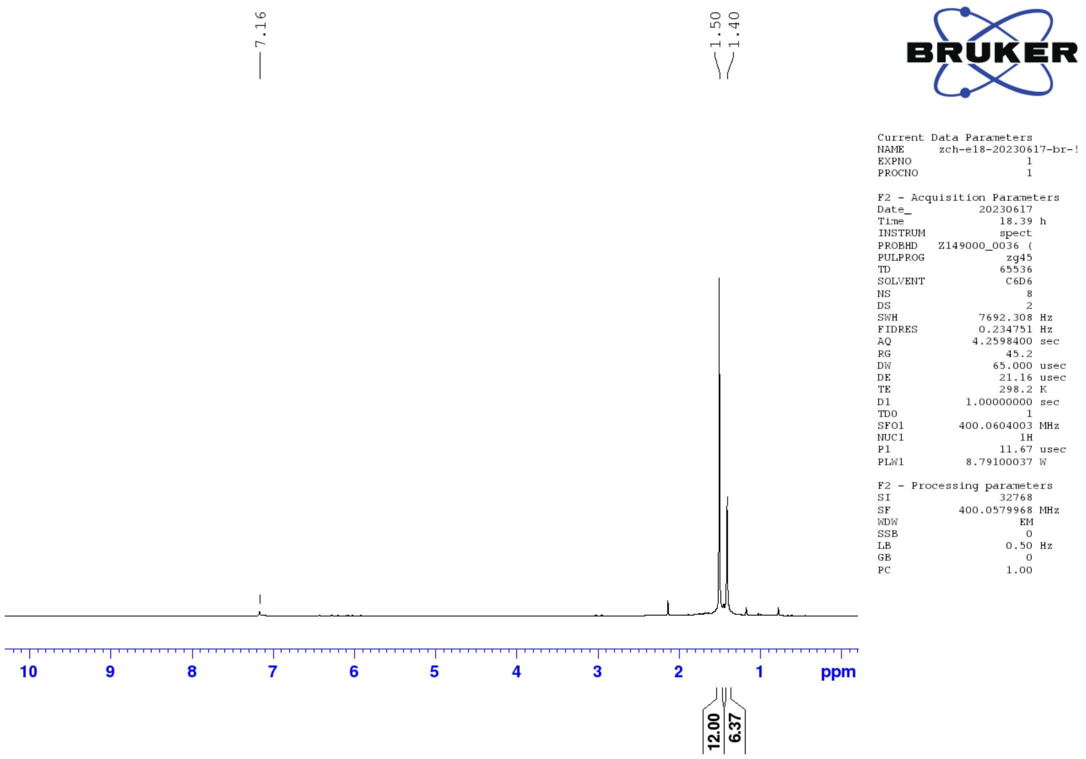

Figure S10. ^{1}H NMR spectrum of **2**^{Br} in C_6D_6

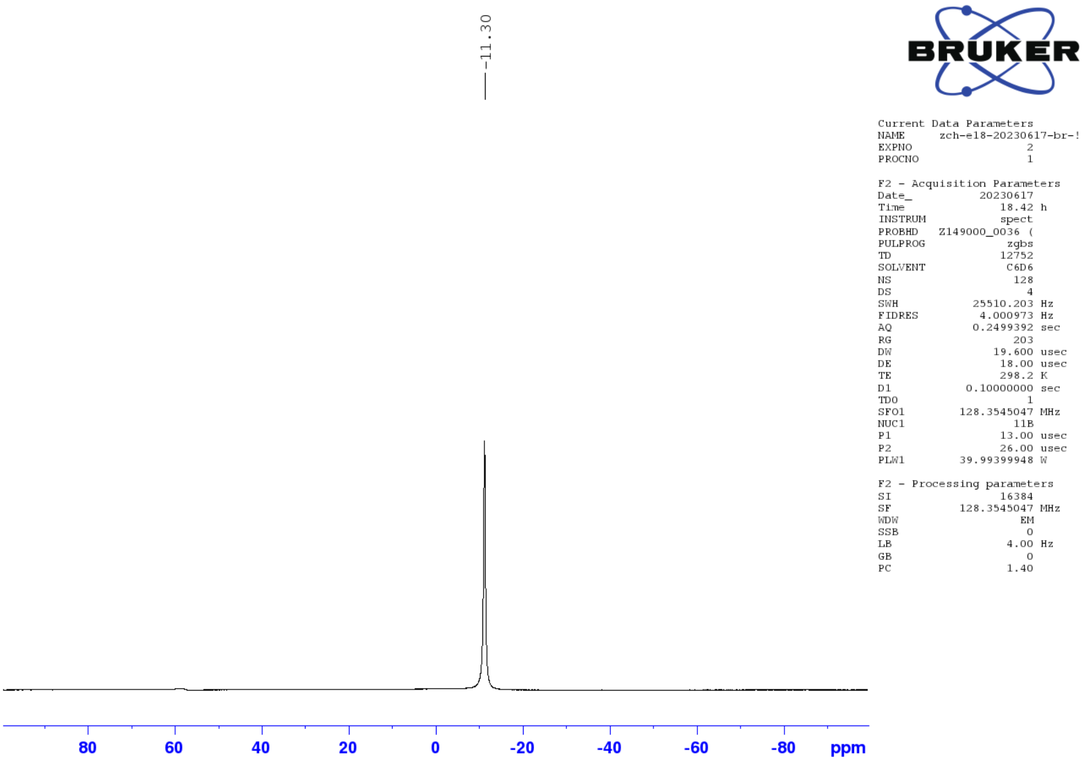

Figure S11. ^{11}B NMR spectrum of **2**^{Br} in C_6D_6

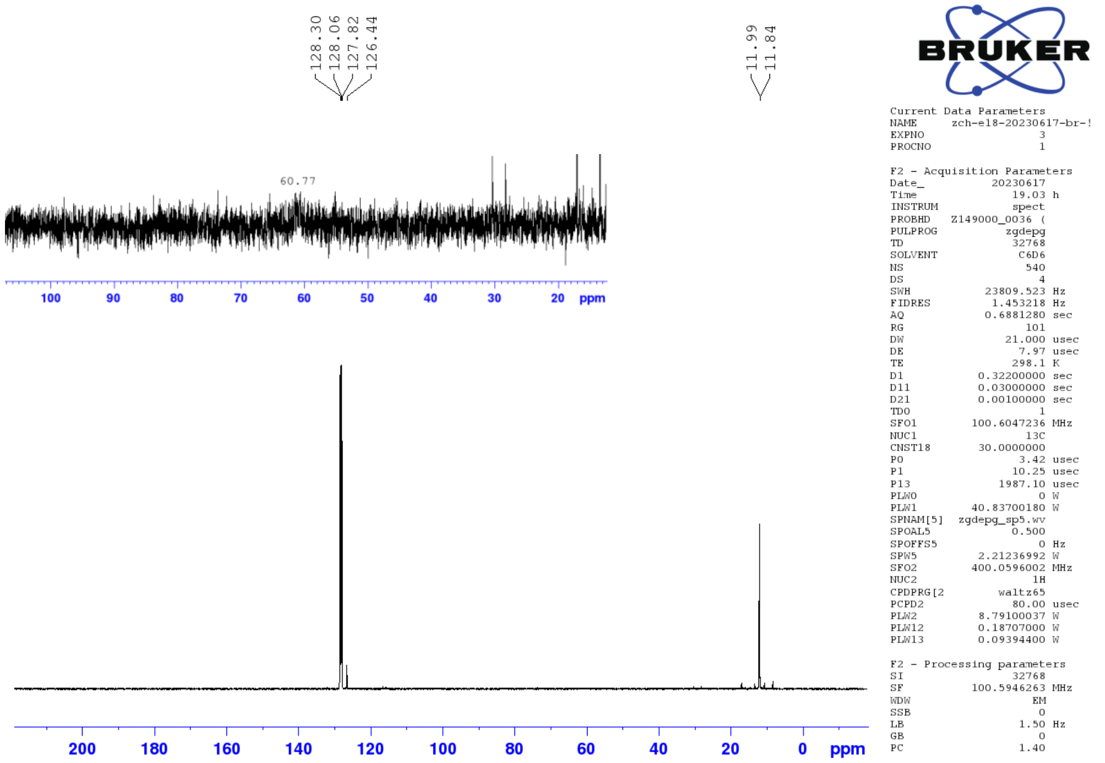

Figure S12. $^{13}C\{^{1}H\}$ NMR spectrum of $\mathbf{2}^{Br}$ in C_6D_6

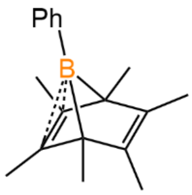

Synthesis of $\mathbf{2}^{Ph}$: inside the glovebox, compound $\mathbf{1}^{Ph}$ (600 mg, 1.53 mmol, 1.0 equiv) and 2-butyne (331 mg, 6.12 mmol, 4.0 equiv) were dissolved in toluene (20 mL) in a Schlenk flask. The flask was taken out of the glovebox and heated at 60 ºC for one day to obtain a light brown solution. All volatiles in the solution were removed under vacuum to obtain an off-white solid. The solid was washed with hexanes (5 mL) and dried under vacuum to obtain the final product. A second crop of the product, a crystalline solid, was obtained by cooling the remaining hexanes solution at –35 ºC (combined yield: 635 mg, 83%). The NMR spectra were in accordance with those in literature.[4]

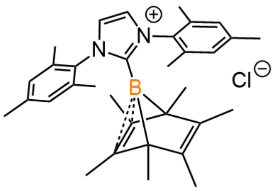

Synthesis of 6: in a vial, a toluene (5 mL) solution of 1,3-bis(2,4,6-trimethylphenyl)-imidazol-2-ylidene (*I*Mes) (182 mg, 0.60 mmol, 1.0 equiv) was slowly added to a toluene (1 mL) solution of $\mathbf{2}^{Cl}$ (125 mg, 0.60 mmol, 1.0 equiv). The solution was left to stand still overnight, and colorless crystalline solids gradually precipitated. The mother liquid was removed, and the remaining solids were dissolved in DCM (0.5 mL). Hexanes (0.5 mL) was slowly added to the DCM solution. The solution was then cooled at –35ºC overnight to obtain colorless crystalline solids (67%, 206 mg).

^{1}H NMR (500 MHz, DCM): 0.95 (s, 6H, B–C–C$\underline{H}_3$), 1.35 (s, 12H, B–C(CH$_3$)–C(C$\underline{H}_3$)), 2.05 (s, 12H, C$^{Mes}\underline{H}_3$), 2.39 (s, 6H, C$^{Mes}\underline{H}_3$), 7.10 (s, 4H, C$^{Mes}\underline{H}$), 7.80 (s, 2H, N–C$\underline{H}$); **^{11}B NMR** (161 MHz, DCM): –16.44 (s); **^{13}C{^{1}H} NMR** (126 MHz, DCM): 11.78 (s, C$^{norbornadiene}\underline{C}H_3$), 12.98 (s, C$^{norbornadiene}\underline{C}H_3$), 18.43 (s, *o*-C$^{Mes}\underline{C}H_3$), 21.22 (s, *p*-C$^{Mes}\underline{C}H_3$), 59.86 (br. s, B–

C(CH$_3$)), 124.38 (s, *p*-C^{Mes}CH$_3$), 126.35 (br. s, B–C(CH$_3$)–C(CH$_3$)), 127.54 (s, N–CH), 130.29 (s, C^{Mes}H), 135.17 (s, *m*-C^{Mes}CH$_3$), 141.80 (s, *o*-C^{Mes}CH$_3$). **HRMS** (m/z): [M]$^+$ calcd. for $C_{33}H_{42}BN_2$, 477.34410; found 477.34156. **Elemental analysis**: calc. for $C_{33}H_{42}BN_2Cl$ + 1.1 DCM, C, 67.54; H, 7.35; N, 4.62; found: C, 67.66; H, 7.40; N, 4.70.

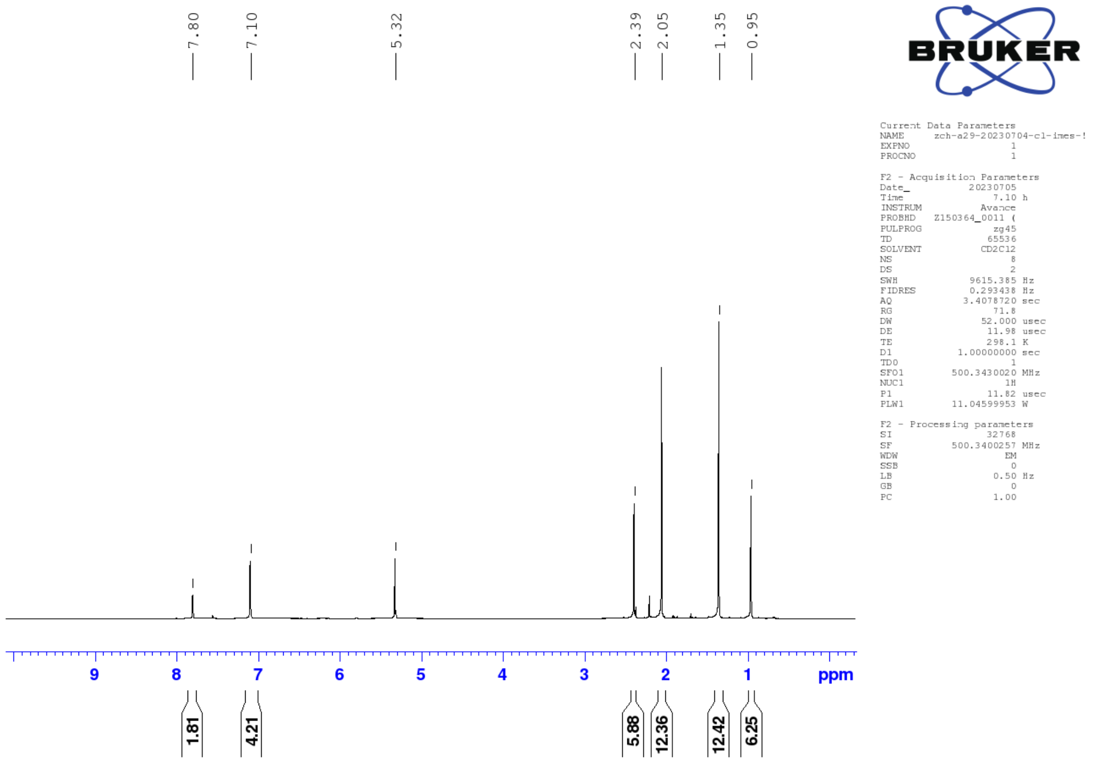

Figure S13. ^{1}H NMR spectrum of **6** in d2-DCM.

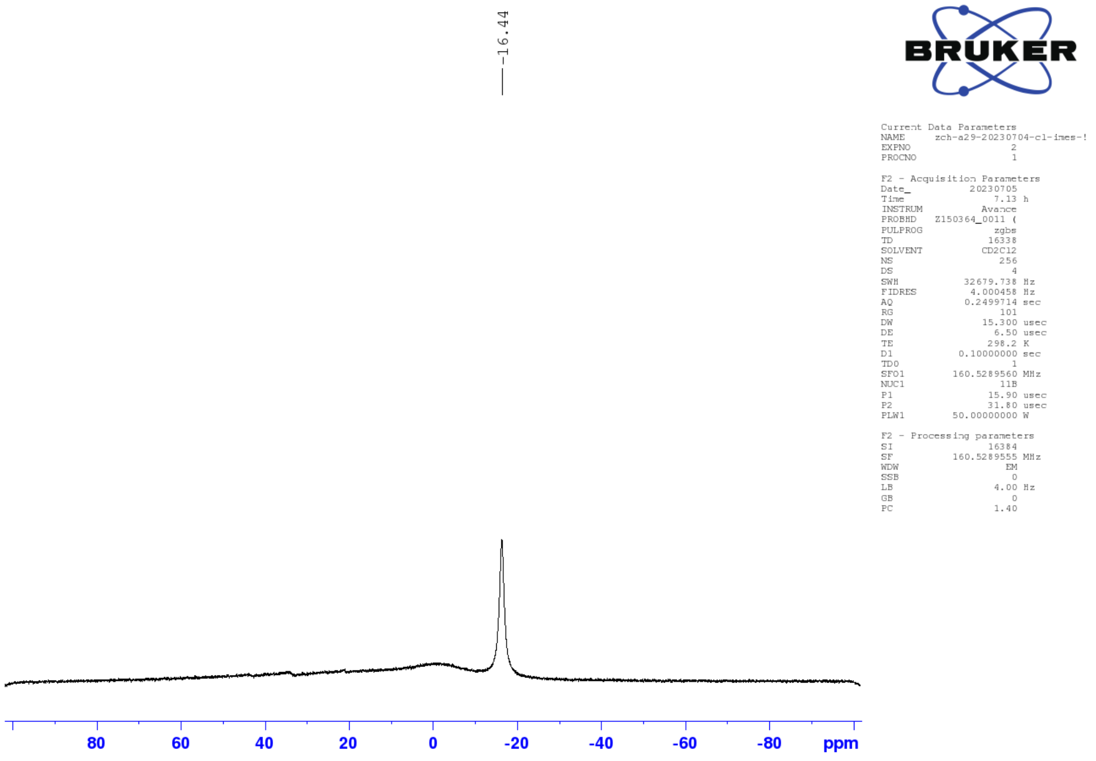

Figure S14. ^{11}B NMR spectrum of **6** in d2-DCM.

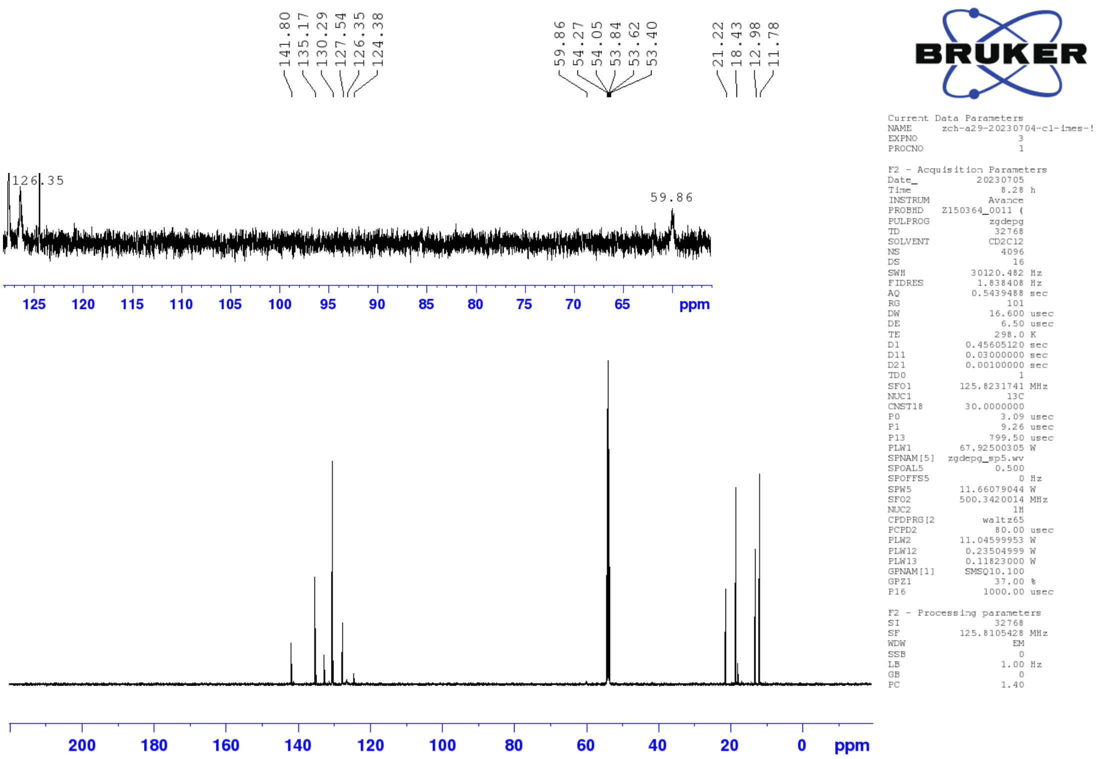

Figure S15. $^{13}C\{^1H\}$ NMR spectrum of **6** in d2-DCM.

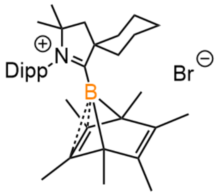

Synthesis of 7: in a vial, a toluene (10 mL) solution of cyclic(alkyl)(amino) carbene ^{Cy}CAAC (604 mg, 1.85 mmol, 1.0 equiv) was slowly added to a toluene (1 mL) solution of $\mathbf{2}^{Br}$ (468 mg, 1.85 mmol, 1.0 equiv). The solution was left to stand still overnight, and yellowish crystalline solids precipitated. The solids were separated from mother liquid by filtration. The solids were washed with toluene (2 mL) and dried under vacuum to afford the final product (56%, 601 mg).

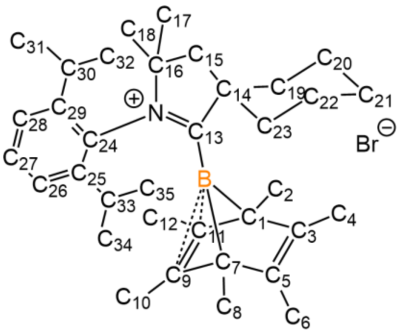

^{1}**H NMR** (500 MHz, DCM): 1.00 (s, 6H, $C_2\underline{H}_3$ and $C_8\underline{H}_3$), 1.23 (d, J = 6.6, 6H, $C_{32}\underline{H}_3$ and $C_{35}\underline{H}_3$,or $C_{31}\underline{H}_3$ and $C_{34}\underline{H}_3$), 1.32 (m, 1H, $C_{21}\underline{H}$), 1.35 (d, J = 6.6, 6H, $C_{32}\underline{H}_3$ and $C_{35}\underline{H}_3$,or $C_{31}\underline{H}_3$ and $C_{34}\underline{H}_3$), 1.35 (br. s, 6H, $C_4\underline{H}_3$ and $C_6\underline{H}_3$,or $C_{10}\underline{H}_3$ and $C_{12}\underline{H}_3$), 1.51 (m, 2H, $C_{20}\underline{H}$ and $C_{22}\underline{H}$), 1.58 (s, 6H, $C_{17}\underline{H}_2$ and $C_{18}\underline{H}_2$), 1.59 (m, 4H, $C_{19}\underline{H}_2$ and $C_{23}\underline{H}_2$), 1.86 (m, 1H, $C_{21}\underline{H}$), 1.91 (m, 2H, $C_{20}\underline{H}$ and $C_{22}\underline{H}$), 2.07 (br. s, 6H, $C_4\underline{H}_3$ and $C_6\underline{H}_3$,or $C_{10}\underline{H}_3$ and $C_{12}\underline{H}_3$), 2.34 (s, 2H, $C_{15}\underline{H}_2$), 2.54 (sept, J = 6.6, 2H, $C_{30}\underline{H}$ and $C_{33}\underline{H}$), 7.35 (d, J = 7.8, 2H, $C_{26}\underline{H}$ and $C_{28}\underline{H}$), 7.53 (t, J = 7.8, 1H, C_{27}); ^{11}**B NMR** (161 MHz, DCM): –13.27 (s); ^{13}**C{^{1}H} NMR** (126

MHz, DCM): 12.60 (br. s, C_{10} and C_{12} ,or C_4 and C_6), 12.96 (s, C_2 and C_8), 14.11 (br. s, C_{10} and C_{12} ,or C_4 and C_6), 22.16 (s, C_{20} and C_{22}), 24.90 (s, C_{21}), 24.14 (s, C_{31} and C_{34} ,or C_{32} and C_{35}), 27.92 (s, C_{31} and C_{34} ,or C_{32} and C_{35}), 29.44 (s, C_{30} and C_{33}), 35.60 (s, C_{19} and C_{23}), 35.65 (s, C_{17} and C_{18}), 45.21 (s, C_{15}), 61.53 (br. s, C_1 and C_7), 61.68 (s, C_{14}), 84.26 (s, C_{16}), 125.52 (br. s, C_3 and C_5, or C_9 and C_{11}), 126.42 (s, C_{27}), 131.60 (s, C_{26} and C_{28}), 131.61 (br. s, C_3 and C_5, or C_9 and C_{11}), 133.84 (s, C_{25} and C_{29}), 145.91 (s, C_{24}), 209.70 (only observed by HMBC, C_{13}). (Some assignments of the peaks were accomplished by the assistance of 2D spectra) **HRMS** (m/z): $[M]^+$ calcd. for $C_{35}H_{53}BN$, 498.42710; found 498.42844. **Elemental analysis**: calcd. for $C_{35}H_{53}BNBr$, C, 72.67; H, 9.23; N, 2.42; found, C, 72.83; H. 9.14; N, 2.28.

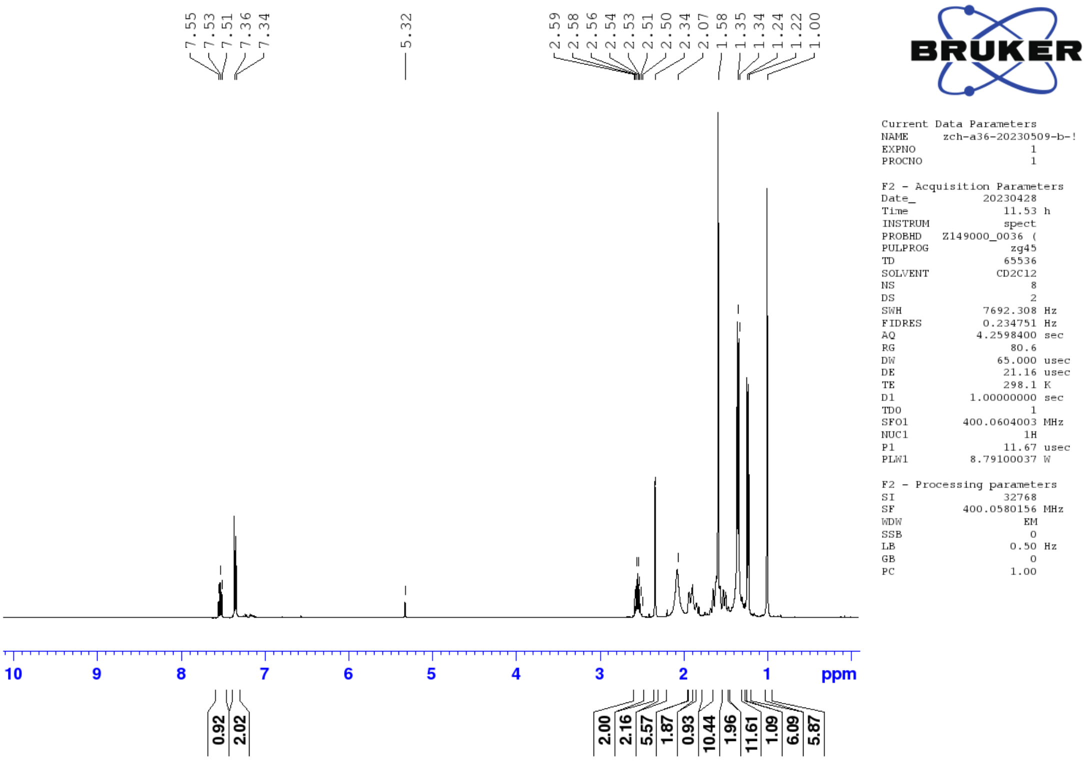

Figure S16. ^1H NMR spectrum of **7** in d2-DCM.

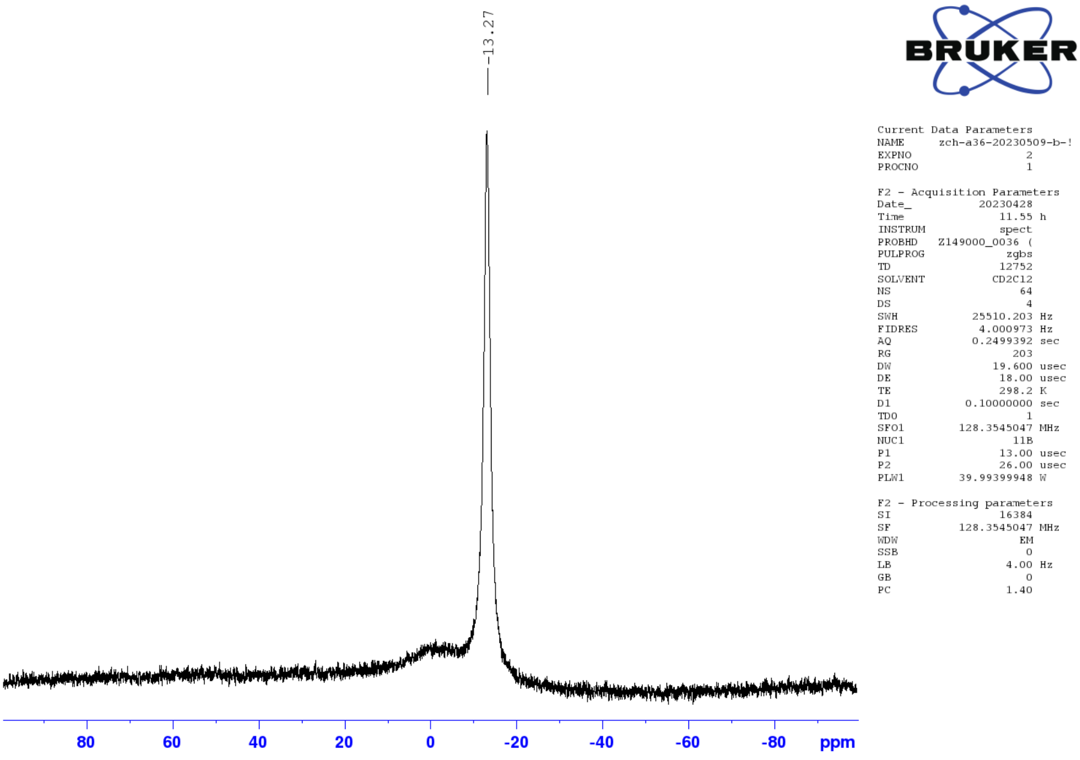

Figure S17. ^{11}B NMR spectrum of **7** in d2-DCM.

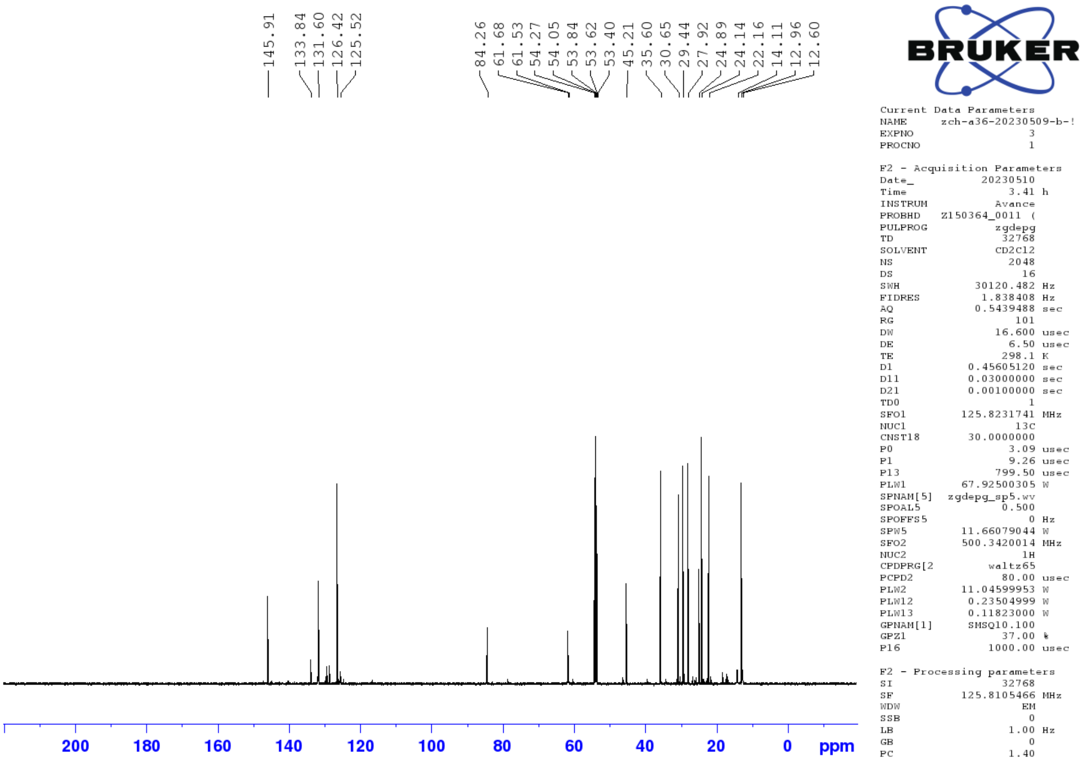

Figure S18. $^{13}C^{8}$ NMR spectrum of **7** in d2-DCM.

The reaction of 2^{Cl} with ^{Et}CAAC: to a toluene (100 mL) solution of $\mathbf{2}^{Cl}$ (505 mg, 2.42 mmol, 1.0 equiv), a toluene (20 mL) solution of ^{Et}CAAC (1.22 g, 2.42 mmol, 1.0 equiv) was slowly added at 25 ºC while stirring. The solution immediately turned orange and then gradually

turned yellow. The solution was stirred overnight and then concentrated to approx. 20 mL. The concentrated solution was cooled at –35 ºC for approx. 20 minutes and compound **3** was precipitated as white crystalline solids (59 mg, 6.8%). After compound **3** was isolated from the mother liquid by filtration, the solution was concentrated to approx. 10 mL and stored at –35 ºC for 2 days. Compound **3**, **4**, and hexamethylbenzene precipitated as a mixture. The mother liquid was removed and the remaining solids were washed with hexanes (10 mL×2) and dried to afford compound **3** and **4** as a ratio of 1:1.4 (172 mg in total).

NMR yield determination: to a toluene (1 mL) solution of $\mathbf{2}^{Cl}$ (10.4 mg, 0.05 mmol, 1.0 equiv), a toluene (1 mL) solution of ^{Et}CAAC (15.7 mg, 0.05 mmol, 1.0 equiv) was slowly added at 25 ºC while stirring. The solution was stirred overnight. Acenaphthene (7.7 mg, 0.05 mmol, 1.0 equiv) was then added to the solution as an internal reference. All volatiles in the solution were removed under vacuum and the remaining solids were dissolved in deuterated DCM. The ^{1}H NMR spectrum suggested that the yield of **3**, **4** and C_6Me_6 were 37%, 24%, and 64%, respectively.

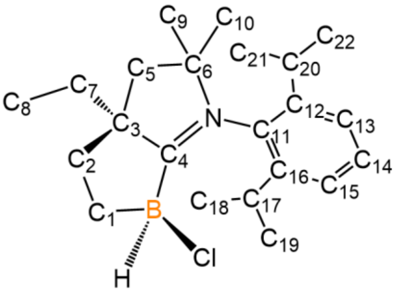

^{1}H NMR (500 MHz, C_6D_6): 0.76 (s, 3H, $C_9\underline{H}_3$ and $C_{10}\underline{H}_3$), 0.92 (t, *J* = 7.3, 3H, $C_8\underline{H}_3$), 1.04 (s, 3H, $C_9\underline{H}_3$ and $C_{10}\underline{H}_3$), 1.07 (d, *J* = 6.7, 3H, $C_{18}\underline{H}_3$ or $C_{19}\underline{H}_3$ or $C_{21}\underline{H}_3$ or $C_{22}\underline{H}_3$), 1.13 (d, *J* = 6.7, 3H, $C_{18}\underline{H}_3$ or $C_{19}\underline{H}_3$ or $C_{21}\underline{H}_3$ or $C_{22}\underline{H}_3$), 1.22 (m, 1H, $C_2\underline{H}$), 1.30 (d, *J* = 6.7, 3H, $C_{18}\underline{H}_3$ or $C_{19}\underline{H}_3$ or $C_{21}\underline{H}_3$ or $C_{22}\underline{H}_3$), 1.40 (d, *J* = 13.2, 1H, $C_5\underline{H}$), 1.53 (d, *J* = 6.7, 3H, $C_{18}\underline{H}_3$ or $C_{19}\underline{H}_3$ or $C_{21}\underline{H}_3$ or $C_{22}\underline{H}_3$), 1.56 to 1.71 (m, 2H, $C_1\underline{H}_2$), 1.76 (d, *J* = 13.2, 1H, $C_5\underline{H}$), 1.83 (dq, *J* = 14.3, *J* = 7.3, 1H, $C_7\underline{H}$), 2.38 (ddd, *J* = 12.7, *J* = 8.8, *J* = 3.2, 1H, $C_2\underline{H}$), 2.46 (sept, *J* = 6.7, 1H, $C_{17}\underline{H}_3$ or $C_{20}\underline{H}_3$), 2.71 (dq, *J* = 14.3, *J* = 7.3, 1H, $C_7\underline{H}$), 2.90 (sept, *J* = 6.7, 1H, $C_{17}\underline{H}_3$ or $C_{20}\underline{H}_3$), 3.08 (br. s, 1H, B$\underline{H}$), 6.98 (dd, *J* = 7.6, *J* = 1.3, 1H, $C_{15}\underline{H}$ or $C_{13}\underline{H}$), 7.03 (dd, *J* = 7.9, *J* = 1.3, 1H, $C_{15}\underline{H}$ or $C_{13}\underline{H}$), 7.11 (t, *J* = 7.7, 1H, $C_{14}\underline{H}$); **^{1}H{^{11}B} NMR** (400 MHz, C_6D_6): 0.76 (s, 3H, $C_9\underline{H}_3$ and $C_{10}\underline{H}_3$), 0.92 (t, *J* = 7.3, 3H, $C_8\underline{H}_3$), 1.04 (s, 3H, $C_9\underline{H}_3$ and $C_{10}\underline{H}_3$), 1.07 (d, *J* = 6.7, 3H, $C_{18}\underline{H}_3$ or $C_{19}\underline{H}_3$ or $C_{21}\underline{H}_3$ or $C_{22}\underline{H}_3$), 1.13 (d, *J* = 6.7, 3H, $C_{18}\underline{H}_3$ or $C_{19}\underline{H}_3$ or $C_{21}\underline{H}_3$ or $C_{22}\underline{H}_3$), 1.22 (m, 1H, $C_2\underline{H}$), 1.30 (d, *J* = 6.7, 3H, $C_{18}\underline{H}_3$ or $C_{19}\underline{H}_3$ or $C_{21}\underline{H}_3$ or $C_{22}\underline{H}_3$), 1.40 (d, *J* = 13.2, 1H, $C_5\underline{H}$), 1.53 (d, *J* = 6.7, 3H, $C_{18}\underline{H}_3$ or $C_{19}\underline{H}_3$ or $C_{21}\underline{H}_3$ or $C_{22}\underline{H}_3$), 1.56 to 1.71 (m, 2H, $C_1\underline{H}_2$), 1.76 (d, *J* = 13.2, 1H, $C_5\underline{H}$), 1.83 (dq, *J* = 14.3, *J* = 7.3, 1H, $C_7\underline{H}$), 2.38 (ddd, *J* = 12.7, *J* = 8.8, *J* = 3.2, 1H, $C_2\underline{H}$), 2.46 (sept, *J* = 6.7, 1H, $C_{17}\underline{H}_3$ or $C_{20}\underline{H}_3$), 2.71 (dq, *J* = 14.3, *J* = 7.3, 1H, $C_7\underline{H}$), 2.90 (sept, *J* = 6.7, 1H, $C_{17}\underline{H}_3$ or $C_{20}\underline{H}_3$), 3.08 (dd, *J* = 8.6, *J* = 3.9, 1H, B$\underline{H}$), 6.98 (dd, *J* = 7.6, *J* = 1.3, 1H, $C_{15}\underline{H}$ or $C_{13}\underline{H}$), 6.03 (dd, *J* = 7.9, *J* = 1.3, 1H, $C_{15}\underline{H}$ or $C_{13}\underline{H}$), 7.11 (t, *J* = 7.7, 1H, $C_{14}\underline{H}$). **^{11}B NMR** (161 MHz, C_6D_6): –10. 87 (d, *J* = 86.0); **^{11}B{^{1}H} NMR** (161 MHz, C_6D_6): –10. 87 (s); **^{13}C{^{1}H} NMR** (126 MHz,

C_6D_6): 9.70 (s, C_8), 20.23 (br. s, C_1), 23.99 (s, C_{18} or C_{19} or C_{21} or C_{22}), 24.36 (s, C_{18} or C_{19} or C_{21} or C_{22}), 25.86 (s, C_{18} or C_{19} or C_{21} or C_{22}), 26.99 (s, C_{18} or C_{19} or C_{21} or C_{22}), 28.32 (s, C_9 or C_{10}), 28.83 (s, C_9 or C_{10}), 29.11 (s, C_{17} or C_{20}), 29.16 (s, C_7), 29.81 (s, C_{17} or C_{20}), 35.82 (s, C_2), 47.05 (s, C_5), 64.76 (s, C_3), 84.83 (s, C_6), 125.02 (s, C_{13} or C_{15}), 125.36 (s, C_{13} or C_{15}), 130.00 (s, C_{14}), 132.39 (s, C_{11}), 145.47 (s, C_{12} or C_{16}), 145.58 (s, C_{12} or C_{16}). (Some assignments of the peaks were accomplished by the assistance of 2D spectra) **HRMS** (m/z): $[M]^+$ calcd. for $C_{22}H_{35}BClN$, 359.25511; found 359.25608. **Elemental analysis**: calcd. for $C_{22}H_{35}BClN$, C, 73.44; H, 9.81; N, 3.89; found: C, 73.71; H, 9.64; N, 3.75.

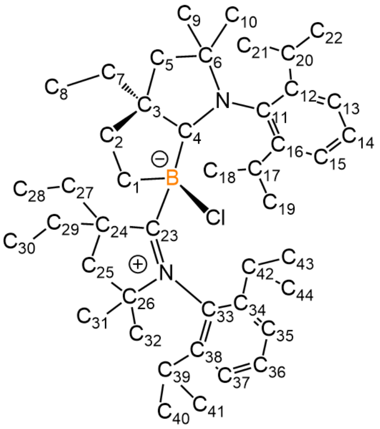

^{1}H NMR (500 MHz, C_6D_6): 0.09 (m, 1H, $C_1\underline{H}$), 0.21 (m, 1H, $C_1\underline{H}$), 0.50 (t, J = 7.4, 3H, $C_{28}\underline{H}_3$ or $C_{30}\underline{H}_3$), 0.67 (s, 3H, $C_9\underline{H}_3$ or $C_{10}\underline{H}_3$), 0.74 (t, J = 7.5, 3H, $C_{28}\underline{H}_3$ or $C_{30}\underline{H}_3$) ,0.75 (s, 3H, $C_9\underline{H}_3$ or $C_{10}\underline{H}_3$), 0.97 (m, 3H, $C_8\underline{H}_3$), 0.98 (d, J = 6.6, 3H, $C_{40}\underline{H}_3$ or $C_{41}\underline{H}_3$ or $C_{43}\underline{H}_3$ or $C_{44}\underline{H}_3$), 1.05 (d, J = 6.6, 3H, $C_{40}\underline{H}_3$ or $C_{41}\underline{H}_3$ or $C_{43}\underline{H}_3$ or $C_{44}\underline{H}_3$), 1.17 (s, 3H, $C_{31}\underline{H}_3$ or $C_{32}\underline{H}_3$), 1.22 (s, 3H, $C_{31}\underline{H}_3$ or $C_{32}\underline{H}_3$), 1.24 (d, J = 6.6, 3H, $C_{40}\underline{H}_3$ or $C_{41}\underline{H}_3$ or $C_{43}\underline{H}_3$ or $C_{44}\underline{H}_3$), 1.25 (d, J = 6.7, 3H, $C_{18}\underline{H}_3$ or $C_{19}\underline{H}_3$ or $C_{21}\underline{H}_3$ or $C_{22}\underline{H}_3$), 1.39 (d, J = 6.8, 3H, $C_{18}\underline{H}_3$ or $C_{19}\underline{H}_3$ or $C_{21}\underline{H}_3$ or $C_{22}\underline{H}_3$), 1.40 (m, 1H, $C_5\underline{H}$), 1.48 (m, 1H, $C_5\underline{H}$), 1.51 (m, 2H, $C_{27}\underline{H}_2$ or $C_{29}\underline{H}_2$), 1.53 (d, J = 6.8, 3H, $C_{18}\underline{H}_3$ or $C_{19}\underline{H}_3$ or $C_{21}\underline{H}_3$ or $C_{22}\underline{H}_3$), 1.63 (d, J = 6.8, 3H, $C_{18}\underline{H}_3$ or $C_{19}\underline{H}_3$ or $C_{21}\underline{H}_3$ or $C_{22}\underline{H}_3$), 1.63 (d, J = 6.5, 3H, $C_{40}\underline{H}_3$ or $C_{41}\underline{H}_3$ or $C_{43}\underline{H}_3$ or $C_{44}\underline{H}_3$), 1.64 (m, 1H, $C_7\underline{H}$), 1.70 (m, 1H, $C_2\underline{H}$), 1.76 (m, 1H, $C_7\underline{H}$), 1.78 (m, 1H, $C_{27}\underline{H}$ or $C_{29}\underline{H}$), 1.90 (m, 1H, $C_{27}\underline{H}$ or $C_{29}\underline{H}$), 1.99 (s, 2H, $C_{25}\underline{H}_2$), 2.45 (sept., J = 6.6, 1H, $C_{39}\underline{H}_3$ or $C_{42}\underline{H}_3$), 2.73 (m, 1H, $C_2\underline{H}$), 3.51 (sept., J = 6.5, 1H, $C_{39}\underline{H}_3$ or $C_{42}\underline{H}_3$), 3.56 (sept., J = 6.7, 1H, $C_{17}\underline{H}_3$ or $C_{20}\underline{H}_3$), 3.62 (s, 1H, $C_4\underline{H}$), 5.02 (sept., J = 6.8, 1H, $C_{17}\underline{H}_3$ or $C_{20}\underline{H}_3$), 6.88 (dd, J = 7.1, J = 2.1, 1H, $C_{35}\underline{H}_3$ or $C_{37}\underline{H}_3$), 7.04 (m, 1H, $C_{35}\underline{H}_3$ or $C_{36}\underline{H}_3$ or $C_{37}\underline{H}_3$), 7.06 (m, 1H, $C_{35}\underline{H}_3$ or $C_{36}\underline{H}_3$ or $C_{37}\underline{H}_3$), 7.15 (m, 1H, $C_{13}\underline{H}_3$ or $C_{14}\underline{H}_3$ or $C_{15}\underline{H}_3$), 7.19 (m, 1H, $C_{13}\underline{H}_3$ or $C_{14}\underline{H}_3$ or $C_{15}\underline{H}_3$), 7.30 (m, 1H, $C_{13}\underline{H}_3$ or $C_{14}\underline{H}_3$ or $C_{15}\underline{H}_3$); **^{11}B NMR** (161 MHz, C_6D_6): 2.95 (s); **^{13}C{^{1}H} NMR** (126 MHz, C_6D_6): 10.84 (s, C_{28} or C_{30}), 10.94 (s, C_{28} or C_{30}), 11.17 (s, C_8), 22.43 (br. s, C_1), 23.69 (s, C_{18} or C_{19} or C_{21} or C_{22}), 24.56 (s, C_{18} or C_{19} or C_{21} or C_{22}), 25.13 (s, C_{40} or C_{41} or C_{43} or C_{44}), 25.68 (s, C_{40} or C_{41} or C_{43} or C_{44}), 26.36 (s, C_9 or C_{10}), 26.88 (s, C_{40} or C_{41} or C_{43} or C_{44}), 27.25 (s, C_{40} or C_{41} or C_{43} or C_{44}), 27.29 (s, C_{17} or C_{20}), 27.57 (s, C_{18} or C_{19} or C_{21} or C_{22}), 27.87 (s, C_{18} or C_{19} or C_{21} or C_{22}), 28.20 (s, C_9 or C_{10}), 28.60 (s, C_{39} or C_{42}), 28.81 (s, C_{39} or C_{42}), 29.65 (s, C_{17} or C_{20}), 30.41 (s, C_{31} or C_{32}), 30.50 (s, C_{31} or C_{32}), 32.73 (s, C_{27} or C_{29}), 32.78 (s, C_2), 33.33 (s, C_7), 34.86 (s, C_{27} or C_{29}), 42.43 (s, C_5), 56.14 (s, C_{24}), 56.34 (s, C_{27} or C_{29}), 56.37 (s, C_3), 61.59 (s, C_{26}), 65.12 (s, C_6), 77.40 (br. s, C_4), 124.87 (s, C_{35} or C_{37}),

124.93 (s, C_{13} or C_{14} or C_{15}), 125.18 (s, C_{13} or C_{14} or C_{15}), 125.31 (s, C_{35} or C_{36} or C_{37}), 126.61 (s, C_{13} or C_{14} or C_{15}), 129.85 (s, C_{35} or C_{36} or C_{37}), 136.52 (s, C_{34} or C_{38}), 145 (s, C_{34} or C_{38}), 146.42 (s, C_{33}), 149.28 (s, C_{12} or C_{16}), 149.60 (s, C_{11}), 152.30 (s, C_{12} or C_{16}). (Some assignments of the peaks were accomplished by the assistance of 2D spectra) **HRMS** (m/z): $[M+H]^+$ calcd. for $C_{44}H_{71}BClN_2$, 673.53988; found 673.54293.

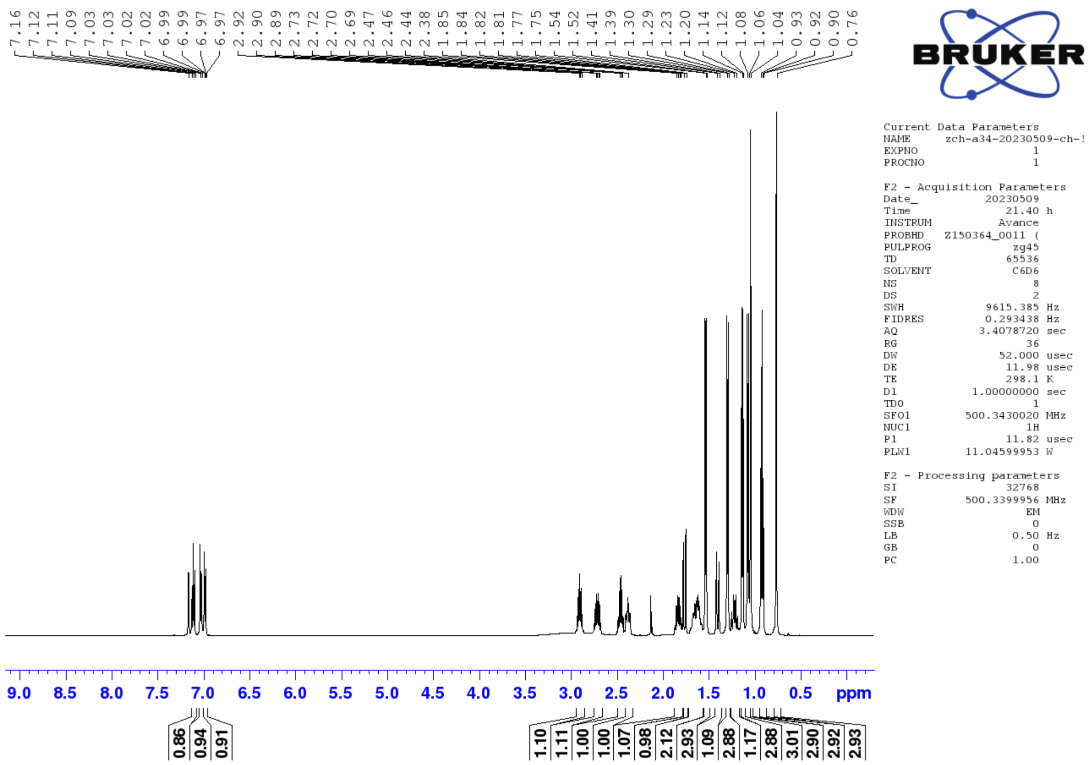

Figure S19. ^1H NMR spectrum of **3** in C_6D_6.

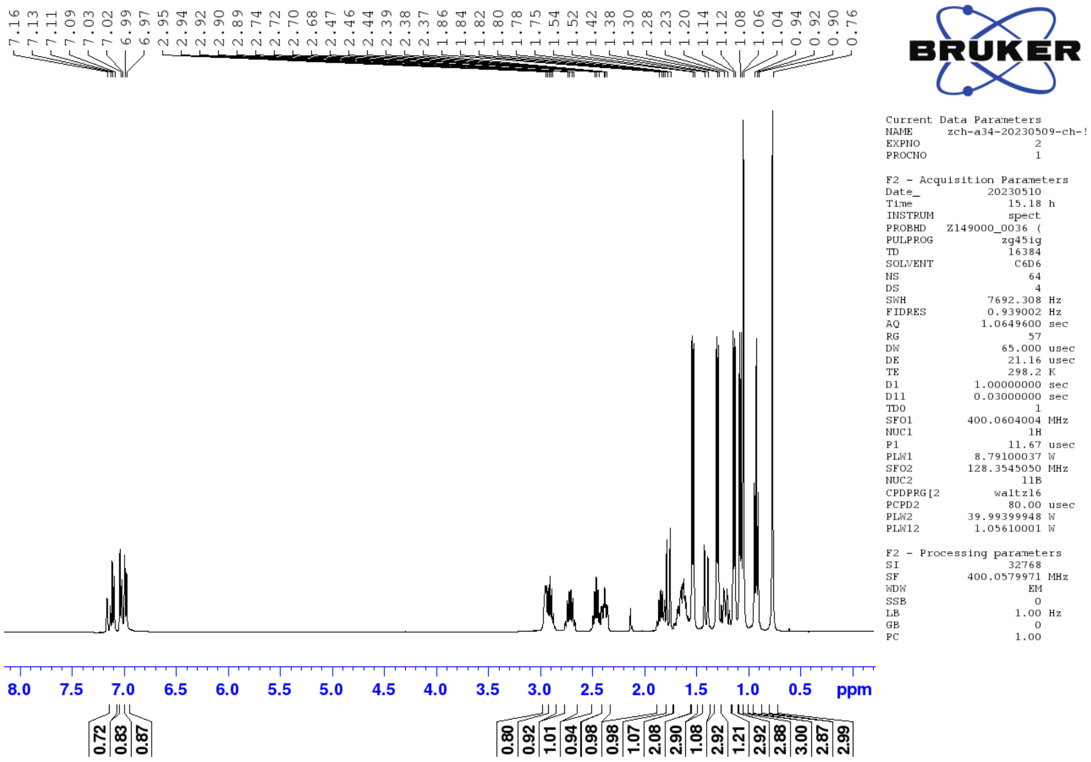

Figure S20. $^{1}H\{^{11}B\}$ NMR spectrum of **3** in C_6D_6.

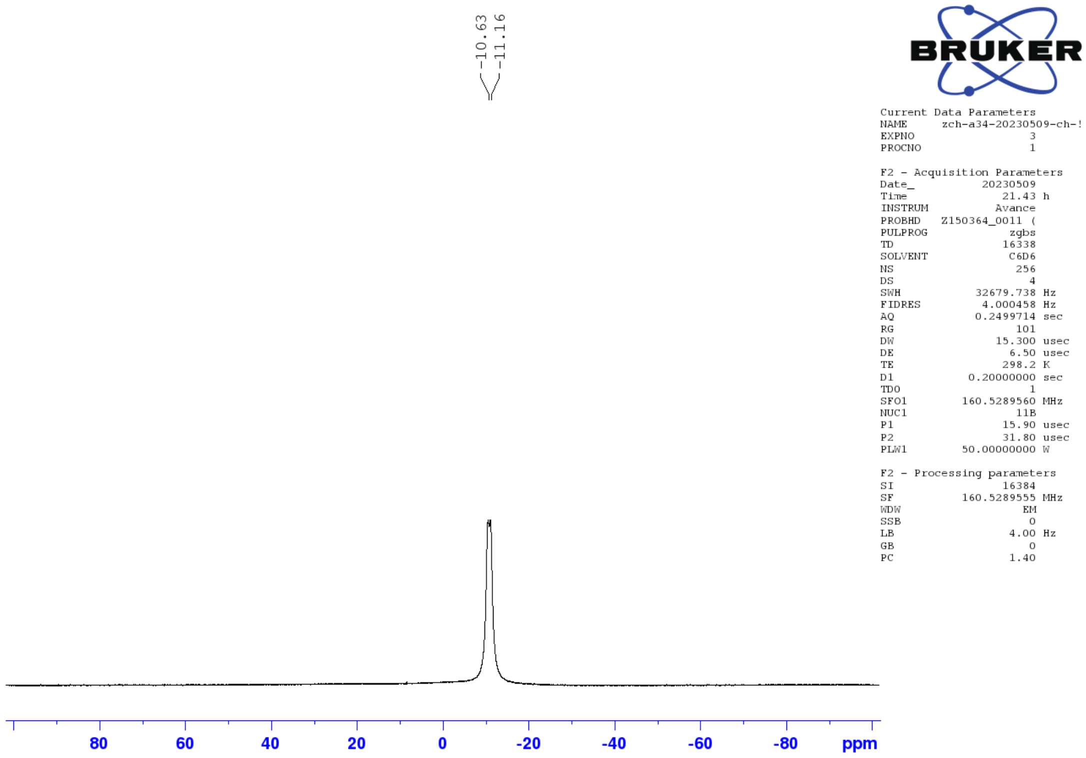

Figure S21. ^{11}B NMR spectrum of **3** in C_6D_6.

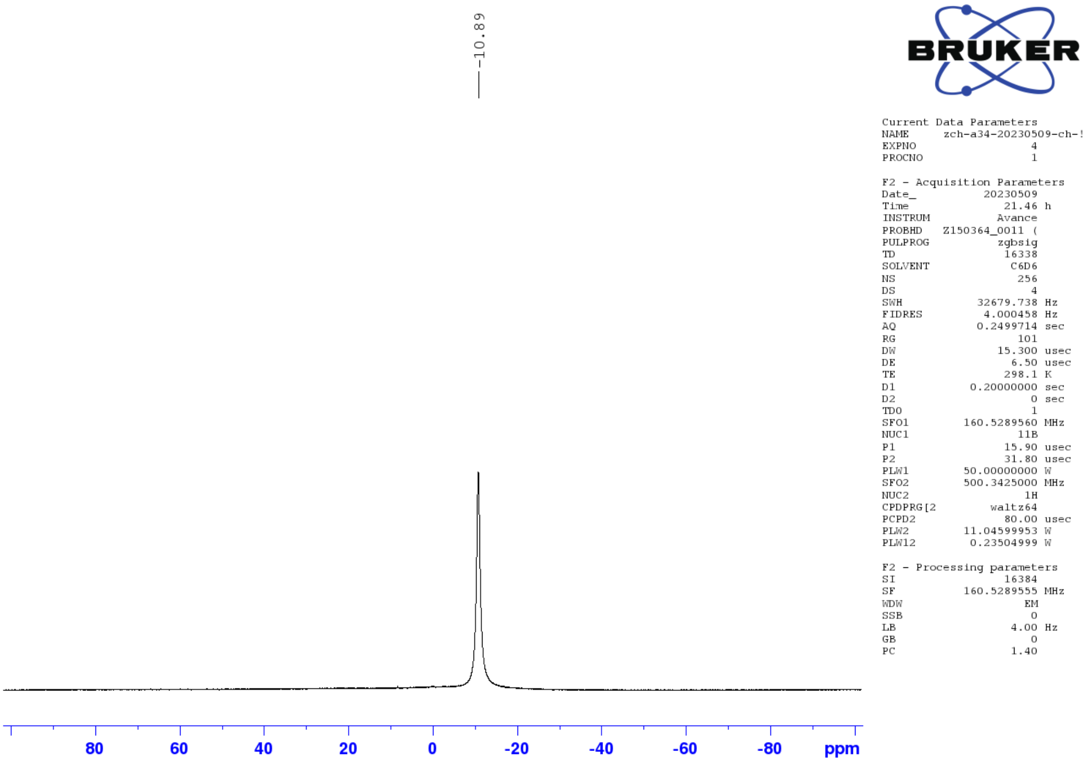

Figure S22. $^{11}B\{^{1}H\}$ NMR spectrum of **3** in C_6D_6.

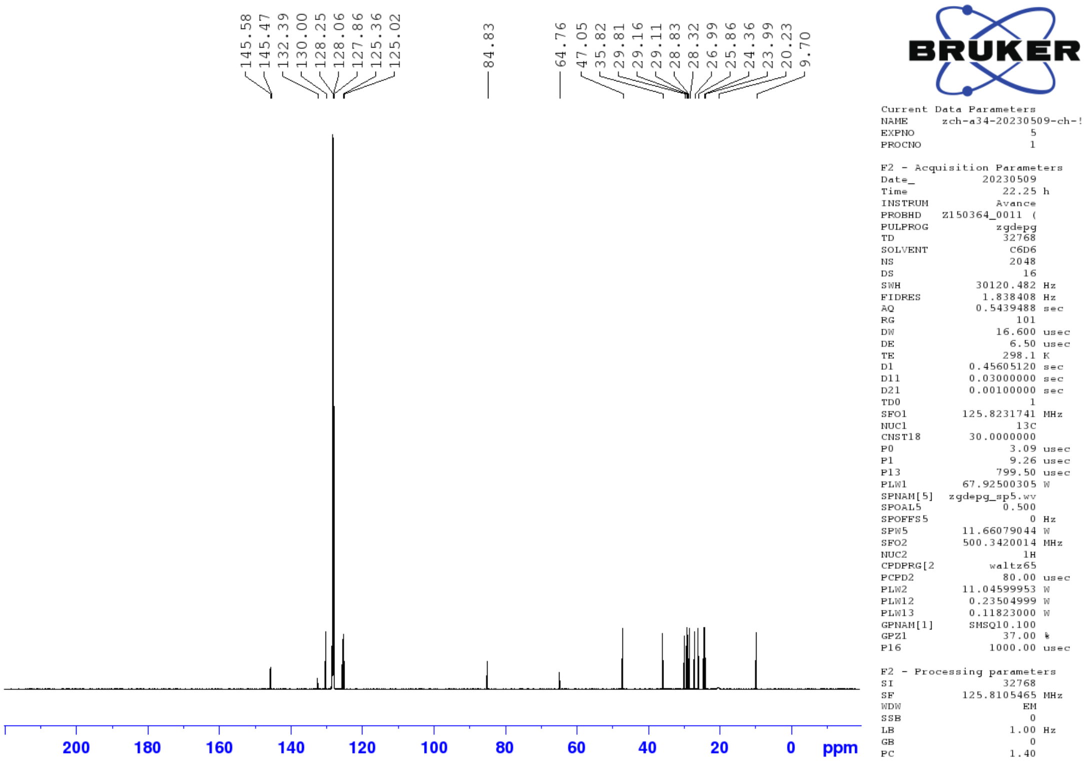

Figure S23. $^{13}C\{^{1}H\}$ NMR spectrum of **3** in C_6D_6.

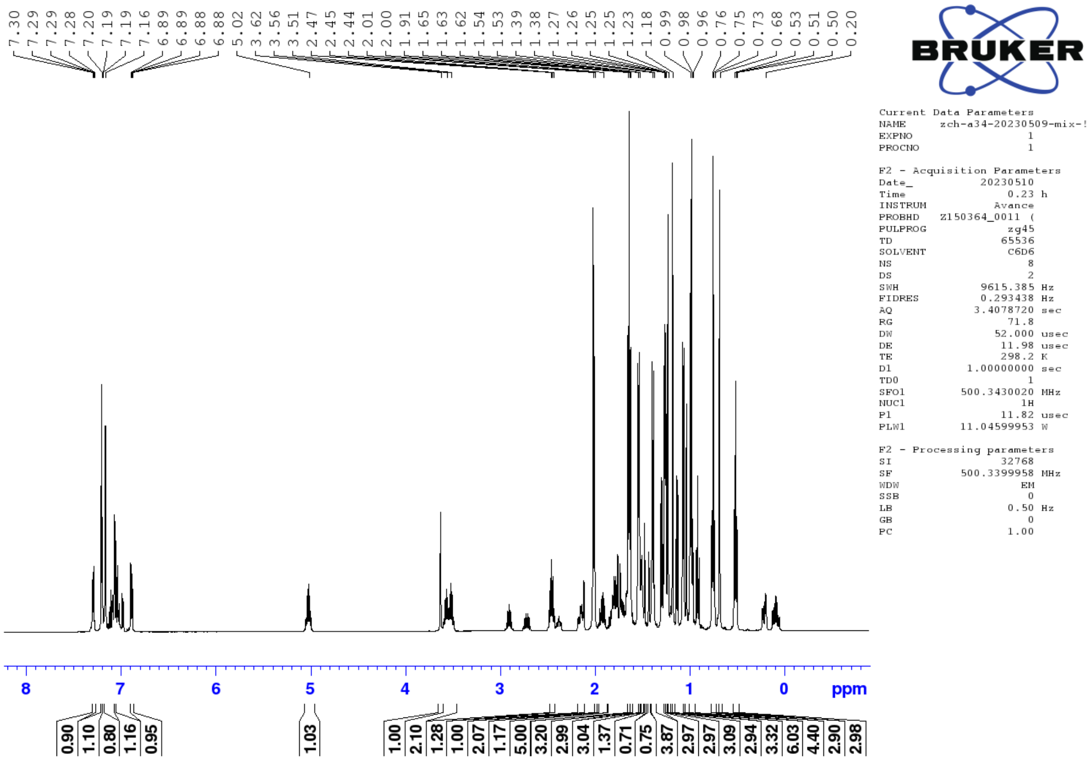

Figure S24. ^{1}H NMR spectrum of mixed **3** and **4** in C_6D_6. Only the signals belonging to **4** were labeled and integrated.

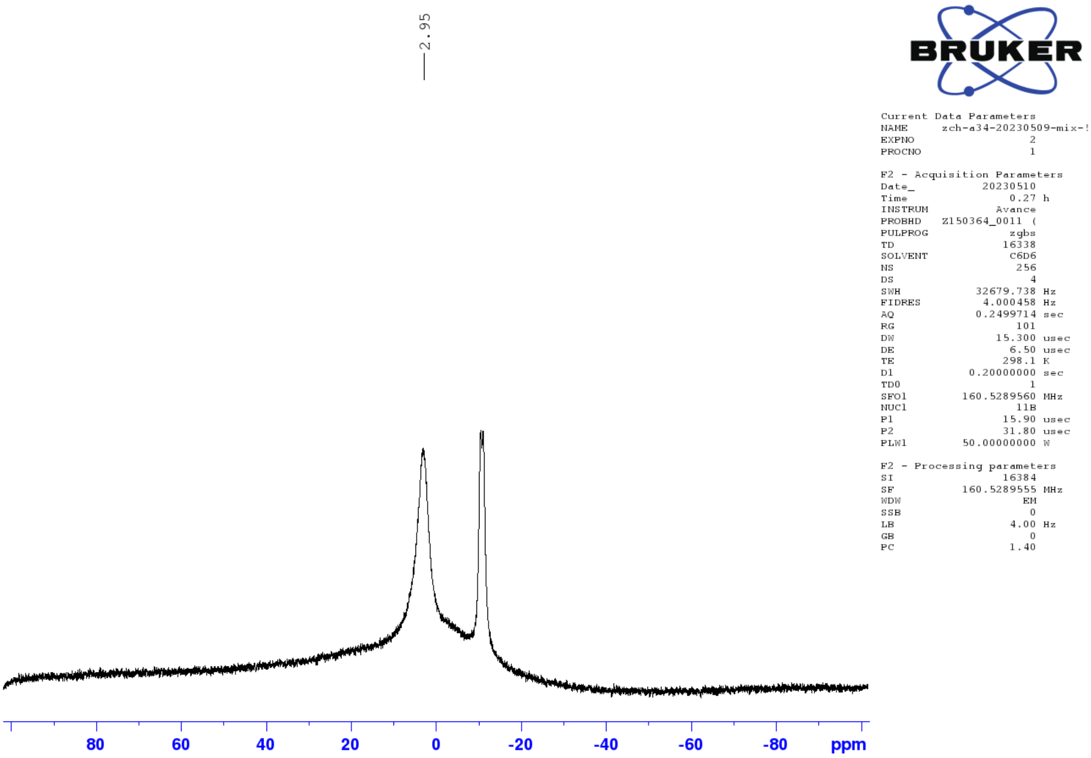

Figure S25. ^{11}B NMR spectrum of mixed **3** and **4** in C_6D_6. Only the signals belonging to **4** were labeled.

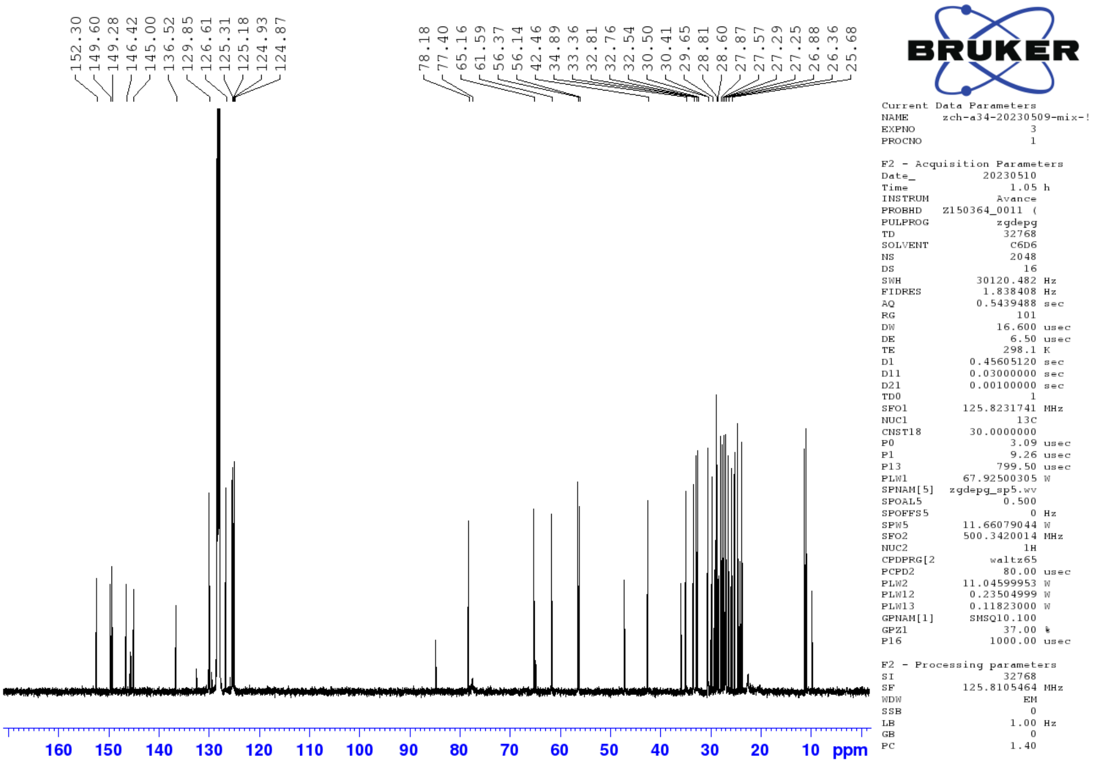

Figure S26. $^{13}C\{^{1}H\}$ NMR spectrum of mixed **3** and **4** in C_6D_6. Only the signals belonging to **4** were labeled.

The reaction of 2^{Cl} with ^{Cy}CAAC: to a toluene (60 mL) solution of $\mathbf{2}^{Cl}$ (371 mg, 1.78 mmol, 1.0 equiv), a toluene (20 mL) solution of ^{Cy}CAAC (580 mg, 1.78 mmol, 1.0 equiv) was slowly added at 25 °C while stirring. The solution immediately turned orange and solids precipitated. The solution was stirred overnight to afford a dark orange suspension. All volatiles in the suspension were removed under reduced pressure, and the remaining solids were washed with hexanes (40 mL×2). The washed solids were dissolved in DCM (2 mL), and hexanes (1 mL) was slowly added. The solution was stored at –35 °C for 2 days and white solids precipitated from the solution. The mother liquid was removed by filtration and the solids were dried under vacuum to afford compound $\mathbf{5}^{Cl}$ (270 mg, 41%) as a white powder.

NMR yield determination: to a toluene (1 mL) solution of $\mathbf{2}^{Cl}$ (10.4 mg, 0.05 mmol, 1.0 equiv), a toluene (1 mL) solution of ^{Cy}CAAC (16.3 mg, 0.05 mmol, 1.0 equiv) was slowly added at 25 °C while stirring. The solution was stirred overnight. Acenaphthene (7.7 mg, 0.05 mmol, 1.0 equiv) was then added to the solution as an internal reference. All volatiles in the solution were removed under vacuum and the remaining solids were dissolved in deuterated DCM. The ^{1}H NMR spectrum suggested that the yield of $\mathbf{5^{Cl}}$ and C_6Me_6 was 62% and 74%, respectively.

The reaction of 7 with [PPN]Cl: compound **7** (28.3 mg, 0.05 mmol, 1.0 equiv) and [PPN]Cl (143 mg, 0.25 mmol, 5.0 equiv) were suspended in toluene and the mixture was stirred overnight at room temperature. All volatiles in the solution were removed under vacuum to

afford a mixture of **5^{Cl}**, [PPN]Br, and C_6Me_6. The ^{1}H NMR spectrum (in C_6D_6) suggested that the ratio of **5^{Cl}** and C_6Me_6 was 1.0:1.1.

The product was characterized as two diastereomers.

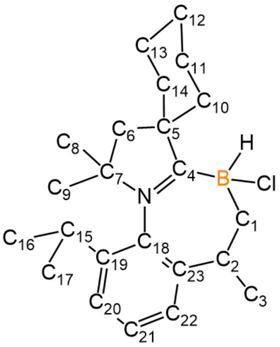

Major Isomer (67%):

^{1}H NMR (500 MHz, C_6D_6): 0.70 (s, 3H, $C_8\underline{H}_3$ or $C_9\underline{H}_3$), 0.93 (s, 3H, $C_8\underline{H}_3$ or $C_9\underline{H}_3$), 1.02 (d, J = 6.6, 3H, $C_3\underline{H}_3$ or $C_{16}\underline{H}_3$ or $C_{17}\underline{H}_3$), 1.07 (tt, J = 12.7, J = 3.6, 2H, $C_{12}\underline{H}_3$), 1.13 (d, J = 6.9, 3H, $C_3\underline{H}_3$ or $C_{16}\underline{H}_3$ or $C_{17}\underline{H}_3$), 1.15 (1H, $C_{10}\underline{H}$ or $C_{14}\underline{H}$), 1.32 (d, J = 6.6, 3H, $C_3\underline{H}_3$ or $C_{16}\underline{H}_3$ or $C_{17}\underline{H}_3$), 1.34 (1H, $C_{10}\underline{H}$ or $C_{14}\underline{H}$), 1.56 (1H, $C_6\underline{H}$), 1.56 (2H, $C_{11}\underline{H}$ or $C_{13}\underline{H}$), 1.59 (m, 2H, $C_1\underline{H}_2$), 1.59 (2H, $C_{11}\underline{H}$ or $C_{13}\underline{H}$), 1.70 (d, J = 13.7, 1H, $C_6\underline{H}$), 2.56 (m, 1H, $C_2\underline{H}$ or $C_{15}\underline{H}$), 2.65 (sept, J = 6.9, 1H, $C_2\underline{H}$ or $C_{15}\underline{H}$), 3.00 (br. s, 1H, B$\underline{H}$), 3.37 (dt, J = 13.5, J = 4.0, 1H, $C_{10}\underline{H}$ or $C_{14}\underline{H}$), 3.43 (dt, J = 13.4, J = 4.0, 1H, $C_{10}\underline{H}$ or $C_{14}\underline{H}$), 7.04 (m, 1H, $C_{20}\underline{H}$ or $C_{21}\underline{H}$ or $C_{22}\underline{H}$), 7.07 (m, 1H, $C_{20}\underline{H}$ or $C_{21}\underline{H}$ or $C_{22}\underline{H}$), 7.15 (m, 1H, $C_{20}\underline{H}$ or $C_{21}\underline{H}$ or $C_{22}\underline{H}$); **^{1}H{^{11}B} NMR** (400 MHz, C_6D_6): 0.70 (s, 3H, $C_8\underline{H}_3$ or $C_9\underline{H}_3$), 0.93 (s, 3H, $C_8\underline{H}_3$ or $C_9\underline{H}_3$), 1.02 (d, J = 6.6, 3H, $C_3\underline{H}_3$ or $C_{16}\underline{H}_3$ or $C_{17}\underline{H}_3$), 1.07 (tt, J = 12.7, J = 3.6, 2H, $C_{12}\underline{H}_3$), 1.13 (d, J = 6.9, 3H, $C_3\underline{H}_3$ or $C_{16}\underline{H}_3$ or $C_{17}\underline{H}_3$), 1.15 (1H, $C_{10}\underline{H}$ or $C_{14}\underline{H}$), 1.32 (d, J = 6.6, 3H, $C_3\underline{H}_3$ or $C_{16}\underline{H}_3$ or $C_{17}\underline{H}_3$), 1.34 (1H, $C_{10}\underline{H}$ or $C_{14}\underline{H}$), 1.56 (1H, $C_6\underline{H}$), 1.56 (2H, $C_{11}\underline{H}$ or $C_{13}\underline{H}$), 1.59 (m, 2H, $C_1\underline{H}_2$), 1.59 (2H, $C_{11}\underline{H}$ or $C_{13}\underline{H}$), 1.70 (d, J = 13.7, 1H, $C_6\underline{H}$), 2.56 (m, 1H, $C_2\underline{H}$ or $C_{15}\underline{H}$), 2.65 (sept, J = 6.9, 1H, $C_2\underline{H}$ or $C_{15}\underline{H}$), 3.01 (m, 1H, B$\underline{H}$), 3.37 (dt, J = 13.5, J = 4.0, 1H, $C_{10}\underline{H}$ or $C_{14}\underline{H}$), 3.43 (dt, J = 13.4, J = 4.0, 1H, $C_{10}\underline{H}$ or $C_{14}\underline{H}$), 7.04 (m, 1H, $C_{20}\underline{H}$ or $C_{21}\underline{H}$ or $C_{22}\underline{H}$), 7.07 (m, 1H, $C_{20}\underline{H}$ or $C_{21}\underline{H}$ or $C_{22}\underline{H}$), 7.15 (m, 1H, $C_{20}\underline{H}$ or $C_{21}\underline{H}$ or $C_{22}\underline{H}$); **^{11}B NMR** (161 MHz, C_6D_6): –7.95 (br. d, J = 67.6); **^{11}B{^{1}H} NMR** (161 MHz, C_6D_6): –7.99 (s); **^{13}C{^{1}H} NMR** (126 MHz, C_6D_6): 20.86 (s, $\underline{C}_3$ or $\underline{C}_{16}$ or $\underline{C}_{17}$), 21.74 (s, $\underline{C}_3$ or $\underline{C}_{16}$ or $\underline{C}_{17}$), 22.15 (s, $\underline{C}_{11}$ or $\underline{C}_{13}$), 22.34 (s, $\underline{C}_{12}$), 25.11 (s, $\underline{C}_{11}$ or $\underline{C}_{13}$), 25.88 (s, $\underline{C}_3$ or $\underline{C}_{16}$ or $\underline{C}_{17}$), 28.41 (s, $\underline{C}_8$ or $\underline{C}_8$), 29.99 (s, $\underline{C}_8$ or $\underline{C}_9$), 30.10 (s, $\underline{C}_2$ or $\underline{C}_{15}$), 32.01 (s, $\underline{C}_{10}$ or $\underline{C}_{14}$), 34.47 (s, $\underline{C}_2$ or $\underline{C}_{15}$), 34.74 (s, $\underline{C}_{10}$ or $\underline{C}_{14}$), 40.48 (br. s, $\underline{C}_1$), 46.85 (s, $\underline{C}_6$), 58.60 (s, $\underline{C}_5$), 77.57 (s, $\underline{C}_7$), 122.30 (s, $\underline{C}_{20}$ or $\underline{C}_{21}$ or $\underline{C}_{22}$), 124.17 (s, $\underline{C}_{20}$ or $\underline{C}_{21}$ or $\underline{C}_{22}$), 129.78 (s, $\underline{C}_{20}$ or $\underline{C}_{21}$ or $\underline{C}_{22}$), 133.28 (s, $\underline{C}_{18}$ or $\underline{C}_{19}$ or $\underline{C}_{23}$), 143.34 (s, $\underline{C}_{18}$ or $\underline{C}_{19}$ or $\underline{C}_{23}$), 144.37 (s, $\underline{C}_{18}$ or $\underline{C}_{19}$ or $\underline{C}_{23}$).

Minor Isomer (33%):

^{1}H NMR (500 MHz, C_6D_6): 0.58 (s, 3H, $C_8\underline{H}_3$ or $C_9\underline{H}_3$), 1.01 (2H, $C_{11}\underline{H}_2$ or $C_{12}\underline{H}_2$ or $C_{13}\underline{H}_2$), 1.09 (2H, $C_{11}\underline{H}_2$ or $C_{12}\underline{H}_2$ or $C_{13}\underline{H}_2$), 1.12 (3H, $C_3\underline{H}_3$ or $C_{16}\underline{H}_3$ or $C_{17}\underline{H}_3$), 1.25 (1H, $C_1\underline{H}$), 1.30 (3H, $C_3\underline{H}_3$ or $C_{16}\underline{H}_3$ or $C_{17}\underline{H}_3$), 1.39 (s, 3H, $C_8\underline{H}_3$ or $C_9\underline{H}_3$), 1.46 (1H, $C_{10}\underline{H}$ or $C_{14}\underline{H}$), 1.46 (1H, $C_6\underline{H}$), 1.47 (3H, $C_3\underline{H}_3$ or $C_{16}\underline{H}_3$ or $C_{17}\underline{H}_3$), 1.51 (2H, $C_{11}\underline{H}_2$ or $C_{12}\underline{H}_2$ or $C_{13}\underline{H}_2$), 1.61 (1H, $C_6\underline{H}$), 1.73 (1H, $C_{10}\underline{H}$ or $C_{14}\underline{H}$), 1.85 (1H, $C_1\underline{H}$), 1.92 (1H, $C_{10}\underline{H}$ or $C_{14}\underline{H}$), 1.99 (1H, $C_{10}\underline{H}$ or $C_{14}\underline{H}$), 2.75 (1H, $C_2\underline{H}$ or $C_{15}\underline{H}$), 3.46 (1H, $C_2\underline{H}$ or $C_{15}\underline{H}$), 6.99 (m, 1H, $C_{20}\underline{H}$ or $C_{21}\underline{H}$ or $C_{22}\underline{H}$),

7.06 (m, 1H, $C_{20}\underline{H}$ or $C_{21}\underline{H}$ or $C_{22}\underline{H}$), 7.14 (m, 1H, $C_{20}\underline{H}$ or $C_{21}\underline{H}$ or $C_{22}\underline{H}$); **^{1}H{^{11}B} NMR** (400 MHz, C_6D_6): 0.58 (s, 3H, $C_8\underline{H}_3$ or $C_9\underline{H}_3$), 1.01 (2H, $C_{11}\underline{H}_2$ or $C_{12}\underline{H}_2$ or $C_{13}\underline{H}_2$), 1.09 (2H, $C_{11}\underline{H}_2$ or $C_{12}\underline{H}_2$ or $C_{13}\underline{H}_2$), 1.12 (3H, $C_3\underline{H}_3$ or $C_{16}\underline{H}_3$ or $C_{17}\underline{H}_3$), 1.25 (1H, $C_1\underline{H}$), 1.30 (3H, $C_3\underline{H}_3$ or $C_{16}\underline{H}_3$ or $C_{17}\underline{H}_3$), 1.39 (s, 3H, $C_8\underline{H}_3$ or $C_9\underline{H}_3$), 1.46 (1H, $C_{10}\underline{H}$ or $C_{14}\underline{H}$), 1.46 (1H, $C_6\underline{H}$), 1.47 (3H, $C_3\underline{H}_3$ or $C_{16}\underline{H}_3$ or $C_{17}\underline{H}_3$), 1.51 (2H, $C_{11}\underline{H}_2$ or $C_{12}\underline{H}_2$ or $C_{13}\underline{H}_2$), 1.61 (1H, $C_6\underline{H}$), 1.73 (1H, $C_{10}\underline{H}$ or $C_{14}\underline{H}$), 1.85 (1H, $C_1\underline{H}$), 1.92 (1H, $C_{10}\underline{H}$ or $C_{14}\underline{H}$), 1.99 (1H, $C_{10}\underline{H}$ or $C_{14}\underline{H}$), 2.75 (1H, $C_2\underline{H}$ or $C_{15}\underline{H}$), 3.46 (1H, $C_2\underline{H}$ or $C_{15}\underline{H}$), 3.54 (m, 1H, B$\underline{H}$), 6.99 (m, 1H, $C_{20}\underline{H}$ or $C_{21}\underline{H}$ or $C_{22}\underline{H}$), 7.06 (m, 1H, $C_{20}\underline{H}$ or $C_{21}\underline{H}$ or $C_{22}\underline{H}$), 7.14 (m, 1H, $C_{20}\underline{H}$ or $C_{21}\underline{H}$ or $C_{22}\underline{H}$); **^{1}B NMR** (161 MHz, C_6D_6): –4.73 (br. d, J = 137.4); **^{11}B{^{1}H} NMR** (161 MHz, C_6D_6): –4.78 (s); **^{13}C{^{1}H} NMR** (126 MHz, C_6D_6): 19.72 (s, $\underline{C}_3$ or $\underline{C}_{16}$ or $\underline{C}_{17}$), 23.44 (s, $\underline{C}_{11}$ or $\underline{C}_{12}$ or $\underline{C}_{13}$), 24.62 (s, $\underline{C}_{11}$ or $\underline{C}_{12}$ or $\underline{C}_{13}$), 26.14 (s, $\underline{C}_3$ or $\underline{C}_{16}$ or $\underline{C}_{17}$), 26.24 (s, $\underline{C}_{11}$ or $\underline{C}_{12}$ or $\underline{C}_{13}$), 26.94 (s, $\underline{C}_8$ or $\underline{C}_9$), 27.80 (s, $\underline{C}_{11}$ or $\underline{C}_{13}$), 27.96 (s, $\underline{C}_8$ or $\underline{C}_9$), 28.38 (s, $\underline{C}_2$ or $\underline{C}_{15}$), 30.77 (s, $\underline{C}_2$ or $\underline{C}_{15}$), 32.44 (s, $\underline{C}_{10}$ or $\underline{C}_{14}$), 33.41 (s, $\underline{C}_{10}$ or $\underline{C}_{14}$), 37.98 (s, $\underline{C}_1$), 44.14 (s, $\underline{C}_5$), 49.16 (s, $\underline{C}_6$), 71.31 (s, $\underline{C}_7$), 123.15 (s, $\underline{C}_{20}$ or $\underline{C}_{21}$ or $\underline{C}_{22}$), 126.30 (s, $\underline{C}_{20}$ or $\underline{C}_{21}$ or $\underline{C}_{22}$), 126.91 (s, $\underline{C}_{20}$ or $\underline{C}_{21}$ or $\underline{C}_{22}$), 135.03 (s, $\underline{C}_{18}$ or $\underline{C}_{19}$ or $\underline{C}_{23}$), 142.46 (s, $\underline{C}_{18}$ or $\underline{C}_{19}$ or $\underline{C}_{23}$), 146.46 (s, $\underline{C}_{18}$ or $\underline{C}_{19}$ or $\underline{C}_{23}$). (Some assignments of the peaks were accomplished by the assistance of 2D spectra) **HRMS** (m/z): $[M+H]^+$ calcd. for $C_{23}H_{36}BClN$, 372.26849; found 372.26728. **Elemental analysis**: calcd. for $C_{23}H_{35}BClN$, C, 74.30; H, 9.49; N, 3.77; found, C, 74.58; H, 9.29; N, 3.67.

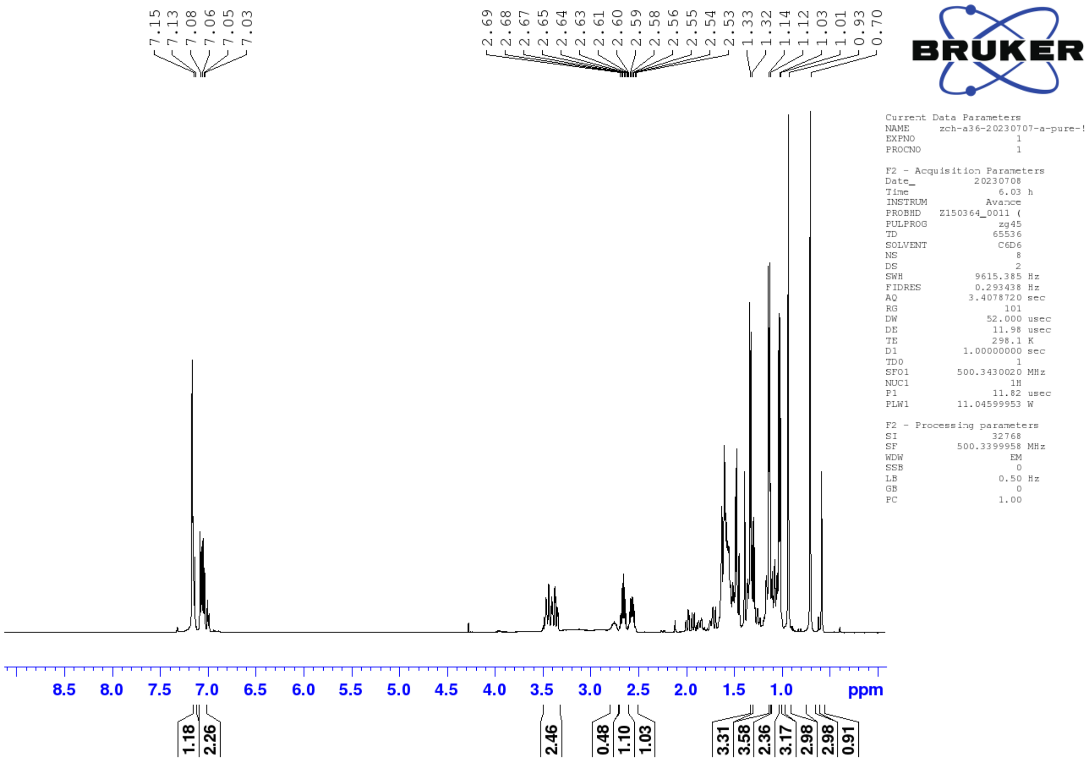

Figure S27. ^{1}H NMR spectrum of $\mathbf{5}^{Cl}$ in C_6D_6. Only the signals belonging to the major isomer were labeled and integrated.

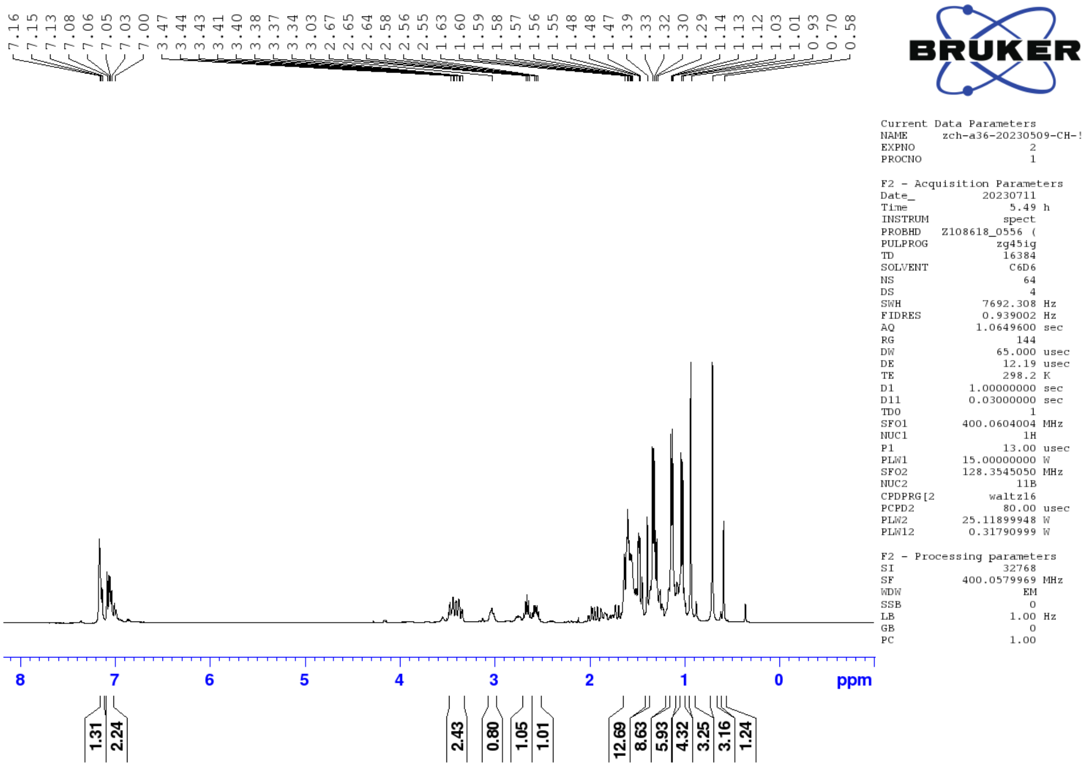

Figure S28. $^{1}H\{^{11}B\}$ NMR spectrum of **5**^{Cl} in C_6D_6. Only the signals belonging to the major isomer were labeled and integrated.

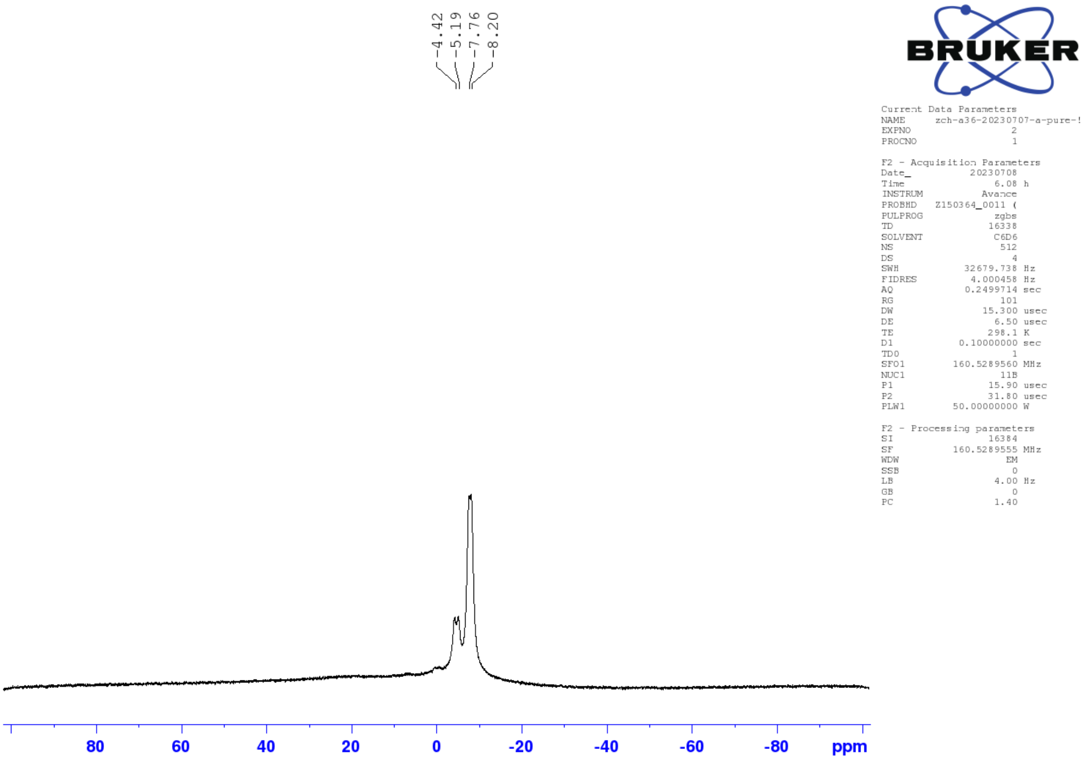

Figure S29. ^{11}B NMR spectrum of **5**^{Cl} in C_6D_6.

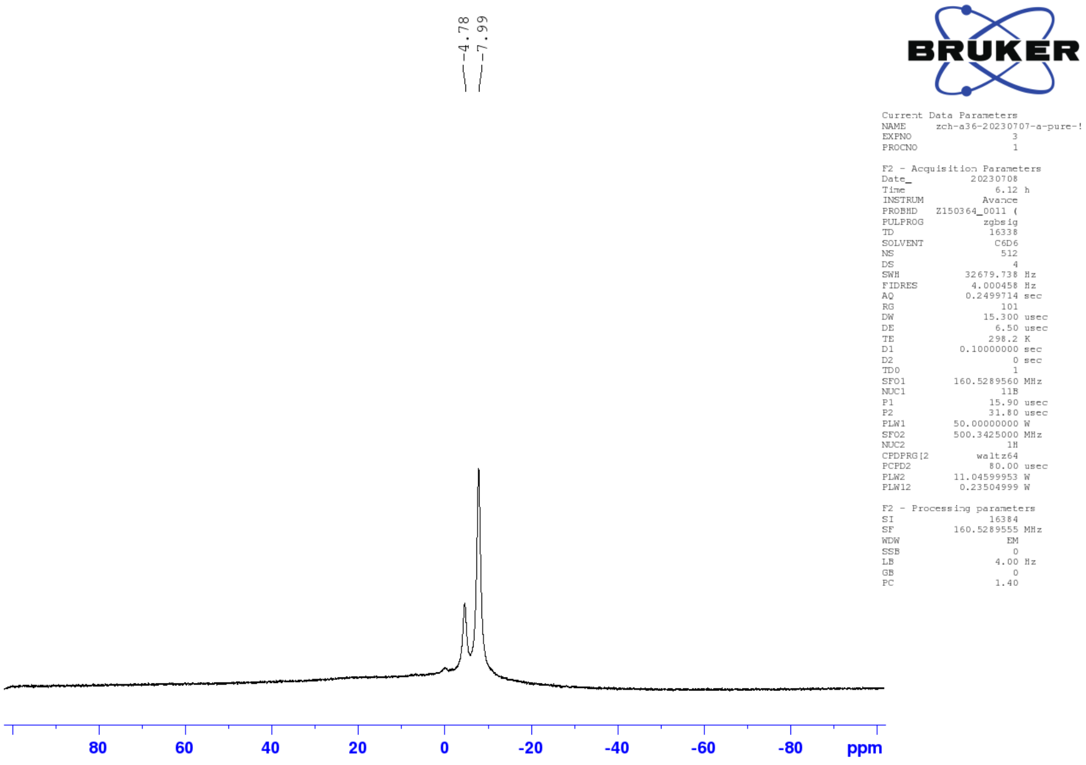

Figure S30. $^{11}B\{^{1}H\}$ NMR spectrum of $\mathbf{5}^{Cl}$ in C_6D_6.

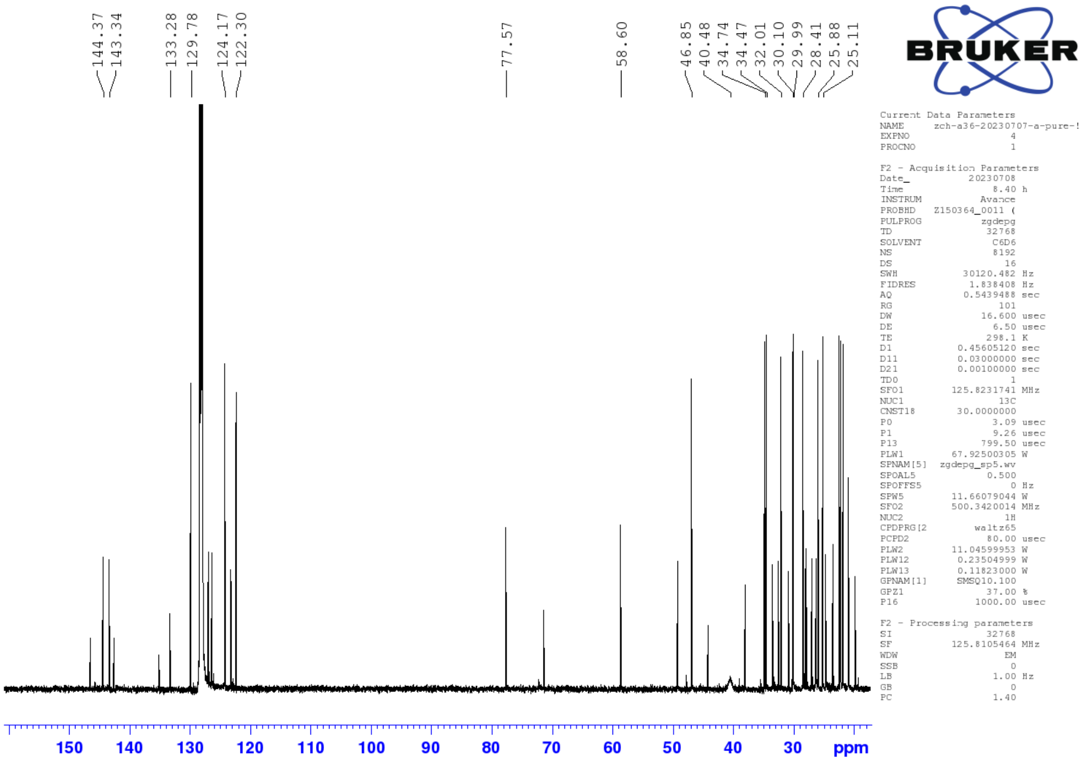

Figure S31. $^{13}C\{^{1}H\}$ NMR spectrum of $\mathbf{5}^{Cl}$ in C_6D_6. Only the signals belonging to the major isomer were labeled.

Heating 7 in toluene: in a Schlenk tube, toluene (20 mL) was added to **7** (587 mg, 1.01 mmol) to form a yellow suspension. The suspension was stirred and heated at 110 °C for

18 hours. The yellow suspension gradually converted to a homogeneous solution. All volatiles in the solution were removed under reduced pressure, and the remaining solids were washed with hexanes (30 mL×2). The washed solids were dissolved in DCM (1 mL), and hexanes (1 mL) was slowly added. The solution was stored at –35 ºC for 2 days and white solids precipitated from the solution. The mother liquid was removed by filtration and the solids were dried to afford compound **5**^{Br} (142 mg, 34%) as a white powder. The two portions of 30 mL hexanes solution were combined and evaporated under vacuum. The remaining solids were sublimed at 100 ºC, 250 mTorr for 4 hours. The remaining solids were dissolved in DCM (0.5 mL), added with hexanes (1.5 mL), and the mixture was stored at –35 ºC for 2 days. The mother liquid was removed, and the remaining crystalline solids were dried under vacuum. The recrystallization process was repeated twice to afford compound **8** (overall yield: 15 mg, 3%).

NMR yield determination: in a Schlenk tube, toluene (1 mL) was added to **7** (28.9 mg, 0.05 mmol, 1.0 equiv) to form a yellow suspension. The suspension was stirred and heated at 110 ºC for 18 hours. The yellow suspension gradually converted to a homogeneous solution. Acenaphthene (7.7 mg, 0.05 mmol, 1.0 equiv) was then added to the solution as an internal reference. All volatiles in the solution were removed under vacuum and the remaining solids were dissolved in deuterated DCM. The ^{1}H NMR spectrum suggested that the yield of **8**, **5^{Br}** and C_6Me_6 were 57%, 35% and 92%, respectively.

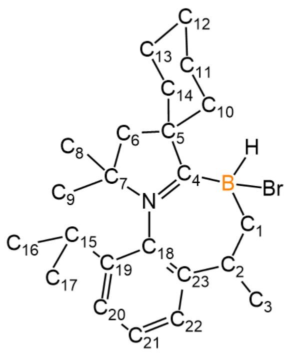

Major Isomer (85%):
^{1}H NMR (500 MHz, C_6D_6): 0.67 (s, 3H, $C_8\underline{H}_3$ or $C_9\underline{H}_3$), 0.92 (s, 3H, $C_8\underline{H}_3$ or $C_9\underline{H}_3$), 1.00 (d, *J* = 6.7, 3H, $C_3\underline{H}_3$ or $C_{16}\underline{H}_3$ or $C_{17}\underline{H}_3$), 1.07 (m, 2H, $C_{11}\underline{H}_2$ or $C_{12}\underline{H}_2$ or $C_{13}\underline{H}_2$), 1.12 (d, *J* = 6.8, 3H, $C_3\underline{H}_3$ or $C_{16}\underline{H}_3$ or $C_{17}\underline{H}_3$), 1.12 (m, 1H, $C_{10}\underline{H}$ or $C_{14}\underline{H}$), 1.30 (m, 1H, $C_{10}\underline{H}$ or $C_{14}\underline{H}$), 1.30 (d, *J* = 6.7, 3H, $C_3\underline{H}_3$ or $C_{16}\underline{H}_3$ or $C_{17}\underline{H}_3$), 1.46 (1H, $C_6\underline{H}$), 1.56 (m, 2H, $C_{11}\underline{H}_2$ or $C_{12}\underline{H}_2$ or $C_{13}\underline{H}_2$), 1.57 (d, *J* = 13.6, 1H, $C_6\underline{H}$), 1.61 (m, 2H, $C_{11}\underline{H}_2$ or $C_{12}\underline{H}_2$ or $C_{13}\underline{H}_2$), 1.63 (m, 1H, $C_1\underline{H}$), 1.73 (m, 1H, $C_1\underline{H}$), 2.52 (sept, *J* = 6.7, 1H, $C_2\underline{H}$ or $C_{15}\underline{H}$), 2.64 (sept, *J* = 6.7, 1H, $C_2\underline{H}$ or $C_{15}\underline{H}$), 3.01 (br. s, 1H, B$\underline{H}$), 3.48 (td, *J* = 13.2, *J* = 3.5, 1H, $C_{10}\underline{H}$ or $C_{14}\underline{H}$), 3.56 (td, *J* = 13.4, *J* = 4.0 1H, $C_{10}\underline{H}$ or $C_{14}\underline{H}$), 7.04 (m, 1H, $C_{20}\underline{H}$ or $C_{21}\underline{H}$ or $C_{22}\underline{H}$), 7.04 (m, 1H, $C_{20}\underline{H}$ or $C_{21}\underline{H}$ or $C_{22}\underline{H}$), 7.13 (m, 1H, $C_{20}\underline{H}$ or $C_{21}\underline{H}$ or $C_{22}\underline{H}$); **^{1}H{^{11}B} NMR** (400 MHz, C_6D_6): 0.67 (s, 3H, $C_8\underline{H}_3$ or $C_9\underline{H}_3$), 0.92 (s, 3H, $C_8\underline{H}_3$ or $C_9\underline{H}_3$), 1.00 (d, *J* = 6.7, 3H, $C_3\underline{H}_3$ or $C_{16}\underline{H}_3$ or $C_{17}\underline{H}_3$), 1.07 (m, 2H, $C_{11}\underline{H}_2$ or $C_{12}\underline{H}_2$ or $C_{13}\underline{H}_2$), 1.12 (d, *J* = 6.8, 3H, $C_3\underline{H}_3$ or $C_{16}\underline{H}_3$ or $C_{17}\underline{H}_3$), 1.12 (m, 1H, $C_{10}\underline{H}$ or $C_{14}\underline{H}$), 1.30 (m, 1H, $C_{10}\underline{H}$ or $C_{14}\underline{H}$), 1.30 (d, *J* = 6.7, 3H, $C_3\underline{H}_3$ or $C_{16}\underline{H}_3$ or $C_{17}\underline{H}_3$), 1.46 (1H, $C_6\underline{H}$), 1.56 (m, 2H, $C_{11}\underline{H}_2$ or $C_{12}\underline{H}_2$ or $C_{13}\underline{H}_2$), 1.57 (d, *J* = 13.6, 1H,

$C_6\underline{H}$), 1.61 (m, 2H, $C_{11}\underline{H}_2$ or $C_{12}\underline{H}_2$ or $C_{13}\underline{H}_2$), 1.63 (m, 1H, $C_1\underline{H}$), 1.73 (m, 1H, $C_1\underline{H}$), 2.52 (sept, *J* = 6.7, 1H, $C_2\underline{H}$ or $C_{15}\underline{H}$), 2.64 (sept, *J* = 6.7, 1H, $C_2\underline{H}$ or $C_{15}\underline{H}$), 3.07 (t, *J* = 8.4 1H, $B\underline{H}$), 3.48 (td, *J* = 13.2, *J* = 3.5, 1H, $C_{10}\underline{H}$ or $C_{14}\underline{H}$), 3.56 (td, *J* = 13.4, *J* = 4.0 1H, $C_{10}\underline{H}$ or $C_{14}\underline{H}$), 7.04 (m, 1H, $C_{20}\underline{H}$ or $C_{21}\underline{H}$ or $C_{22}\underline{H}$), 7.04 (m, 1H, $C_{20}\underline{H}$ or $C_{21}\underline{H}$ or $C_{22}\underline{H}$), 7.13 (m, 1H, $C_{20}\underline{H}$ or $C_{21}\underline{H}$ or $C_{22}\underline{H}$); **^{11}B NMR** (161 MHz, C_6D_6): –10.73 (br. s, FWHM = 353.9 Hz); **^{11}B{^{1}H} NMR** (161 MHz, C_6D_6): –10.59 (br. s, FWHM = 205.9 Hz); **^{13}C{^{1}H} NMR** (126 MHz, C_6D_6): 20.66 (s, $\underline{C}_3$ or $\underline{C}_{16}$ or $\underline{C}_{17}$), 21.74 (s, $\underline{C}_3$ or $\underline{C}_{16}$ or $\underline{C}_{17}$), 22.19 (s, $\underline{C}_{11}$ or $\underline{C}_{12}$ or $\underline{C}_{13}$), 22.35 (s, $\underline{C}_{11}$ or $\underline{C}_{12}$ or $\underline{C}_{13}$), 25.00 (s, $\underline{C}_{11}$ or $\underline{C}_{12}$ or $\underline{C}_{13}$), 25.79 (s, $\underline{C}_3$ or $\underline{C}_{16}$ or $\underline{C}_{17}$), 28.42 (s, $\underline{C}_8$ or $\underline{C}_8$), 29.95 (s, $\underline{C}_8$ or $\underline{C}_9$), 30.11 (s, $\underline{C}_2$ or $\underline{C}_{15}$), 32.07 (s, $\underline{C}_{10}$ or $\underline{C}_{14}$), 34.93 (s, $\underline{C}_2$ or $\underline{C}_{15}$), 35.02 (s, $\underline{C}_{10}$ or $\underline{C}_{14}$), 40.37 (br. s, $\underline{C}_1$), 47.01 (s, $\underline{C}_6$), 58.80 (s, $\underline{C}_5$), 77.57 (s, $\underline{C}_7$), 122.34 (s, $\underline{C}_{20}$ or $\underline{C}_{21}$ or $\underline{C}_{22}$), 124.28 (s, $\underline{C}_{20}$ or $\underline{C}_{21}$ or $\underline{C}_{22}$), 129.88 (s, $\underline{C}_{20}$ or $\underline{C}_{21}$ or $\underline{C}_{22}$), 133.29 (s, $\underline{C}_{18}$ or $\underline{C}_{19}$ or $\underline{C}_{23}$), 143.27 (s, $\underline{C}_{18}$ or $\underline{C}_{19}$ or $\underline{C}_{23}$), 143.96 (s, $\underline{C}_{18}$ or $\underline{C}_{19}$ or $\underline{C}_{23}$). (Some assignments of the peaks were accomplished by the assistance of 2D spectra)
Minor Isomer (15%): most of the peaks are hard to identify.
^{11}B NMR (161 MHz, C_6D_6): –5.38 (br. s); **^{11}B{^{1}H} NMR** (161 MHz, C_6D_6): –5.47 (br. s).
HRMS (m/z): $[M]^+$ calcd. for $C_{23}H_{35}BBrN$, 415.20459; found 415.20369.

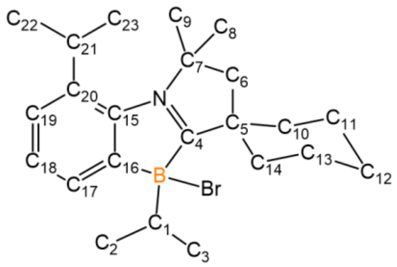

^{1}H NMR (500 MHz, C_6D_6): 0.77 (d, *J* = 6.9, 3H, $C_2\underline{H}_3$ or $C_3\underline{H}_3$), 0.98 (1H, $C_{11}\underline{H}$ or $C_{12}\underline{H}$ or $C_{13}\underline{H}$), 1.00 (1H, $C_{11}\underline{H}$ or $C_{12}\underline{H}$ or $C_{13}\underline{H}$), 1.06 (d, *J* = 6.9, 3H, $C_{22}\underline{H}_3$ or $C_{23}\underline{H}_3$), 1.09 (d, *J* = 6.9, 3H, $C_2\underline{H}_3$ or $C_3\underline{H}_3$), 1.16 (1H, $C_{11}\underline{H}$ or $C_{12}\underline{H}$ or $C_{13}\underline{H}$), 1.17 (s, 3H, $C_8\underline{H}_3$ or $C_9\underline{H}_3$), 1.28 (s, 3H, $C_8\underline{H}_3$ or $C_9\underline{H}_3$), 1.40 (m, 1H, $C_{10}\underline{H}$ or $C_{14}\underline{H}$), 1.45 (1H, $C_{11}\underline{H}$ or $C_{12}\underline{H}$ or $C_{13}\underline{H}$), 1.49 (1H, $C_{11}\underline{H}$ or $C_{12}\underline{H}$ or $C_{13}\underline{H}$), 1.51 (1H, $C_{11}\underline{H}$ or $C_{12}\underline{H}$ or $C_{13}\underline{H}$), 1.74 (d, *J* = 6.8, 3H, $C_2\underline{H}_3$ or $C_3\underline{H}_3$), 1.78 (m, 1H, $C_{10}\underline{H}$ or $C_{14}\underline{H}$), 1.78 (1H, $C_6\underline{H}$), 1.82 (1H, $C_6\underline{H}$), 2.26 (td, *J* = 13.4, *J* = 3.2, 1H, $C_{10}\underline{H}$ or $C_{14}\underline{H}$), 2.86 (td, *J* = 13.9, *J* = 3.6, 1H, $C_{10}\underline{H}$ or $C_{14}\underline{H}$), 3.27 (sept, *J* = 6.8, 1H, $C_{21}\underline{H}$), 7.02 (dd, *J* = 7.8, *J* = 1.3, 1H, $C_{19}\underline{H}$), 7.18 (m, 1H, $C_{18}\underline{H}$), 7.86 (d, *J* = 6.6, 1H, $C_{17}\underline{H}$); **^{11}B NMR** (161 MHz, C_6D_6): –4.71 (s); **^{13}C{^{1}H} NMR** (126 MHz, C_6D_6): 20.31 (s, $\underline{C}_2$ or $\underline{C}_3$), 21.41 (s, $\underline{C}_2$ or $\underline{C}_3$), 22.15 (s, $\underline{C}_{11}$ or $\underline{C}_{12}$ or $\underline{C}_{13}$), 22.44 (s, $\underline{C}_{11}$ or $\underline{C}_{12}$ or $\underline{C}_{13}$), 23.70 (s, $\underline{C}_{22}$ or $\underline{C}_{23}$), 24.81 (s, $\underline{C}_{22}$ or $\underline{C}_{23}$), 25.37 (s, $\underline{C}_{11}$ or $\underline{C}_{12}$ or $\underline{C}_{13}$), 27.67 (s, $\underline{C}_8$ or $\underline{C}_9$), 29.32 (s, $\underline{C}_8$ or $\underline{C}_9$), 29.45 (s, $\underline{C}_{21}$), 33.81 (s, $\underline{C}_{10}$ or $\underline{C}_{14}$), 38.15 (s, $\underline{C}_{10}$ or $\underline{C}_{14}$), 52.66 (s, $\underline{C}_5$), 53.85 (s, $\underline{C}_6$), 70.87 (s, $\underline{C}_7$), 125.89 (s, $\underline{C}_{19}$), 128.35 (s, $\underline{C}_{18}$), 130.70 (s, $\underline{C}_{17}$), 134.69 (s, $\underline{C}_{15}$ or $\underline{C}_{20}$), 141.26 (s, $\underline{C}_{15}$ or $\underline{C}_{20}$). (Some assignments of the peaks were accomplished by the assistance of 2D spectra) **HRMS** (m/z): $[M+H]^+$ calcd. for $C_{23}H_{36}BBrN$, 416.21240; found 416.21000.

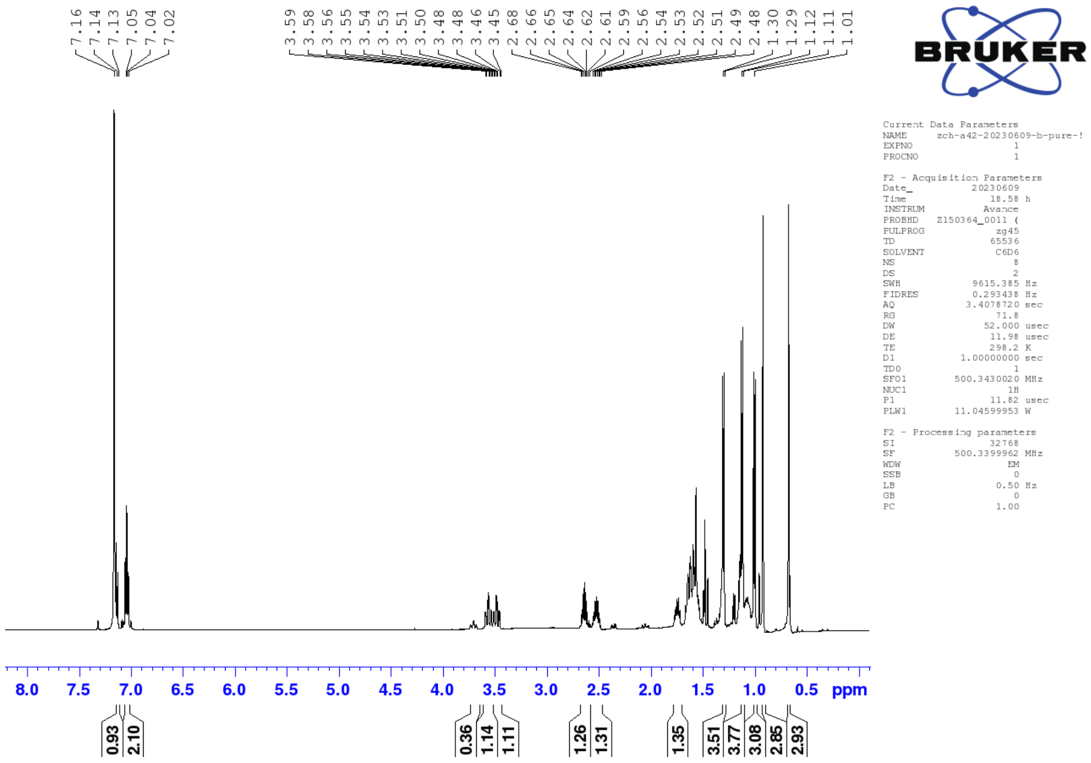

Figure S32. ^{1}H NMR spectrum of **5**^{Br} in C_6D_6. Only the signals belonging to the major isomer were labeled and integrated.

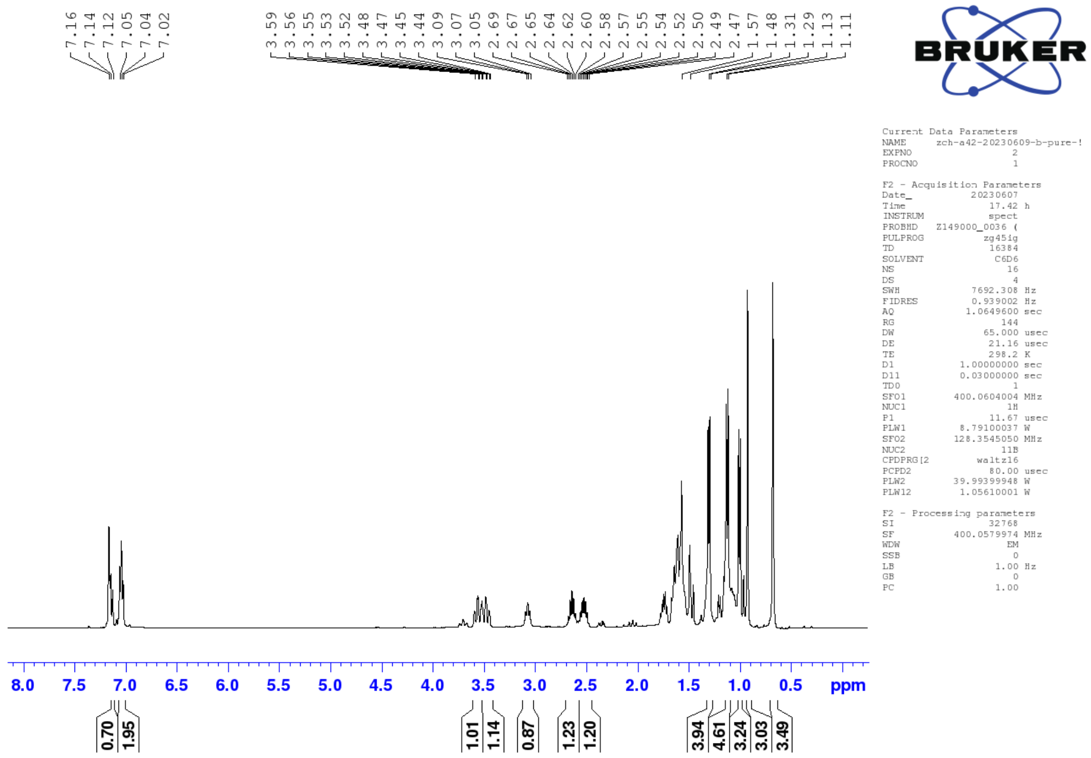

Figure S33. ^{1}H{^{11}B} NMR spectrum of **5**^{Br} in C_6D_6. Only the signals belonging to the major isomer were labeled and integrated.

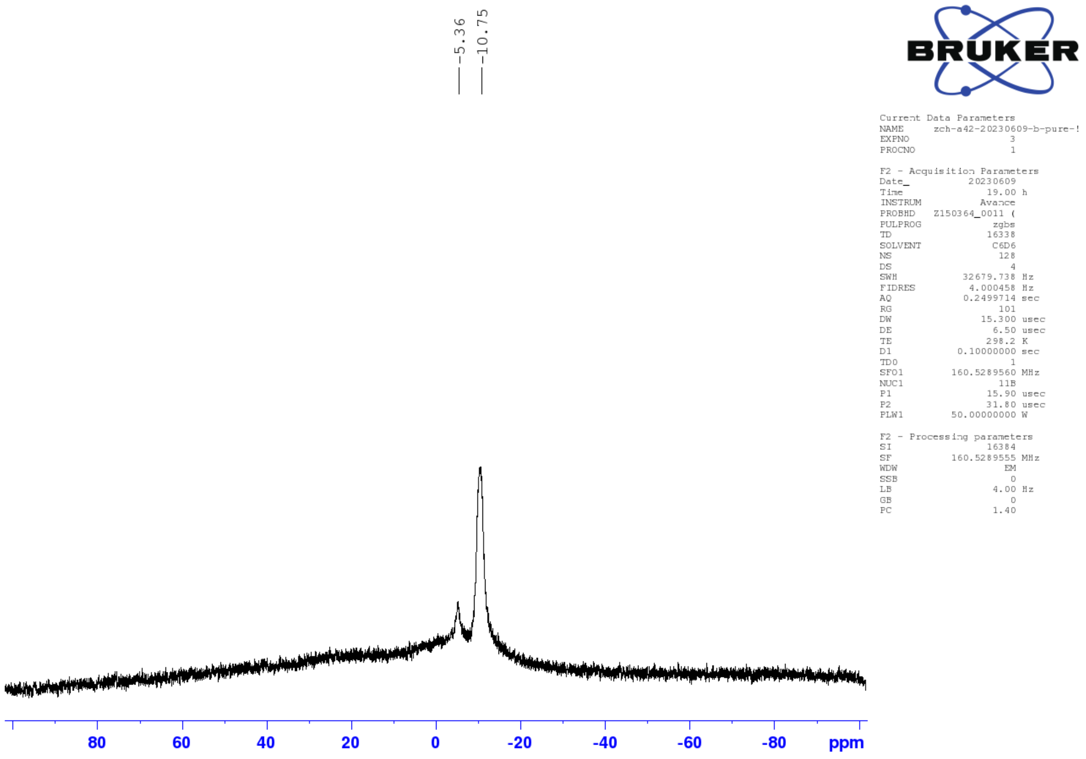

Figure S34. ^{11}B NMR spectrum of $\mathbf{5}^{Br}$ in C_6D_6.

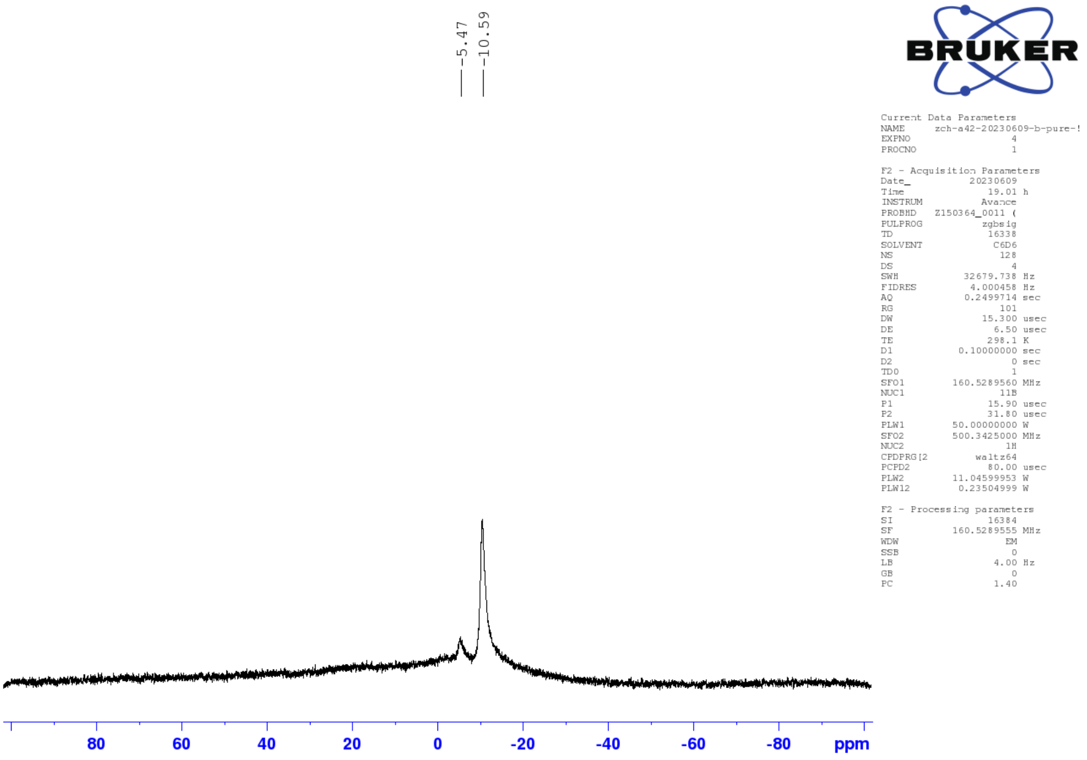

Figure S35. $^{11}B\{^1H\}$ NMR spectrum of $\mathbf{5}^{Br}$ in C_6D_6.

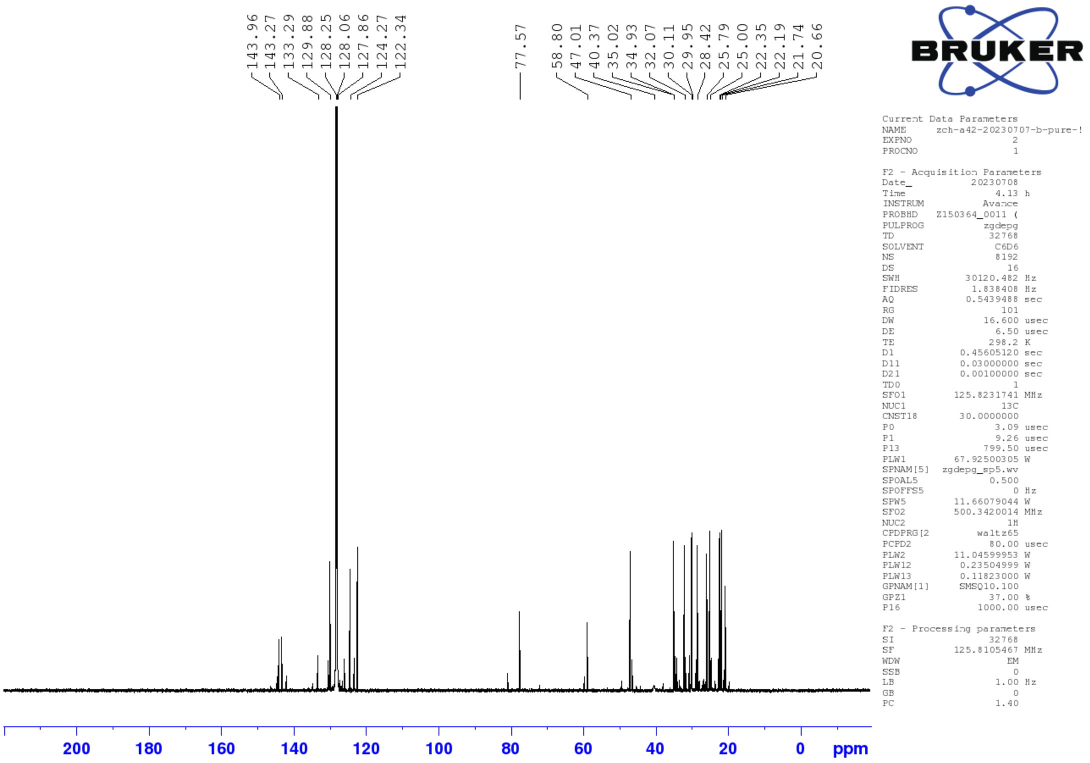

Figure S36. $^{13}C\{^{1}H\}$ NMR spectrum of **5**^{Br} in C_6D_6. Only the signals belonging to the major isomer were labeled.

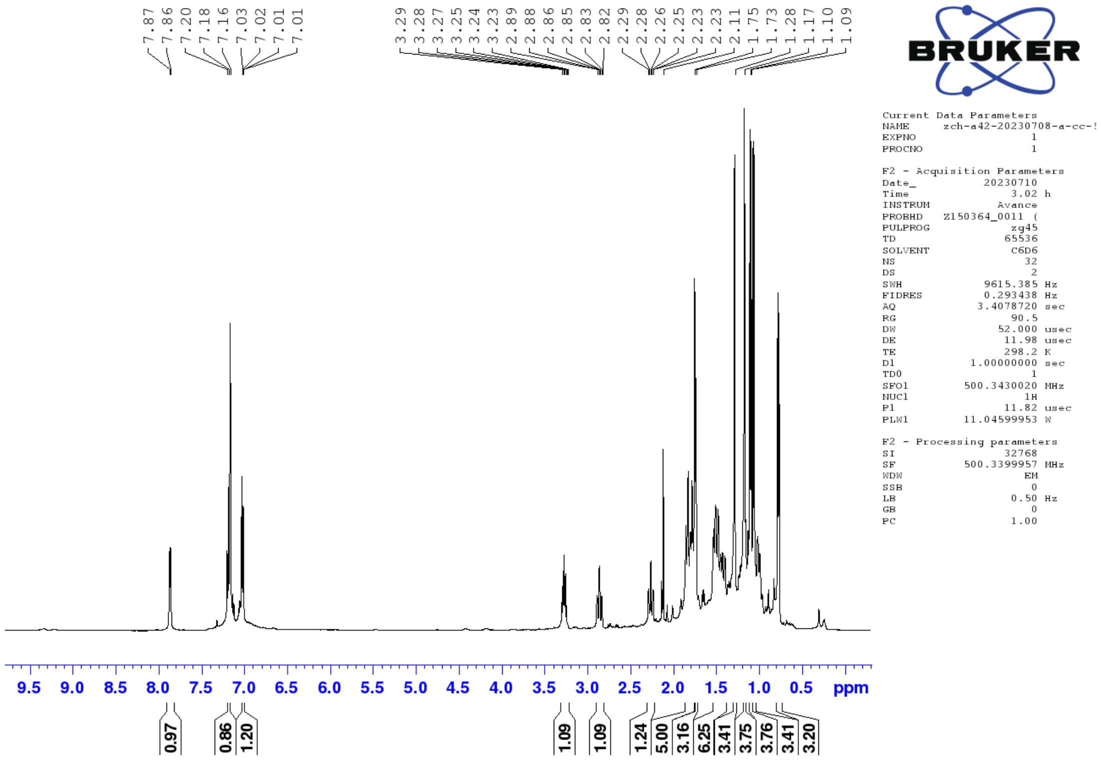

Figure S37. ^{1}H NMR spectrum of **8** in C_6D_6.

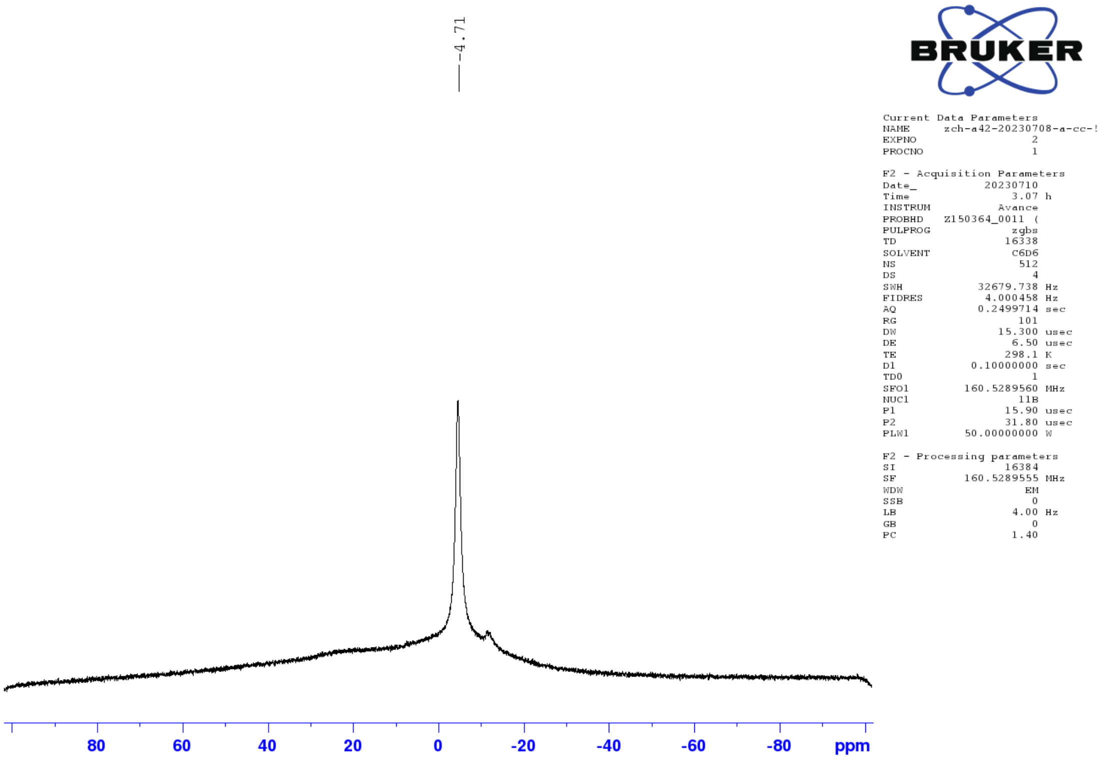

Figure S38. ^{11}B NMR spectrum of **8** in C_6D_6.

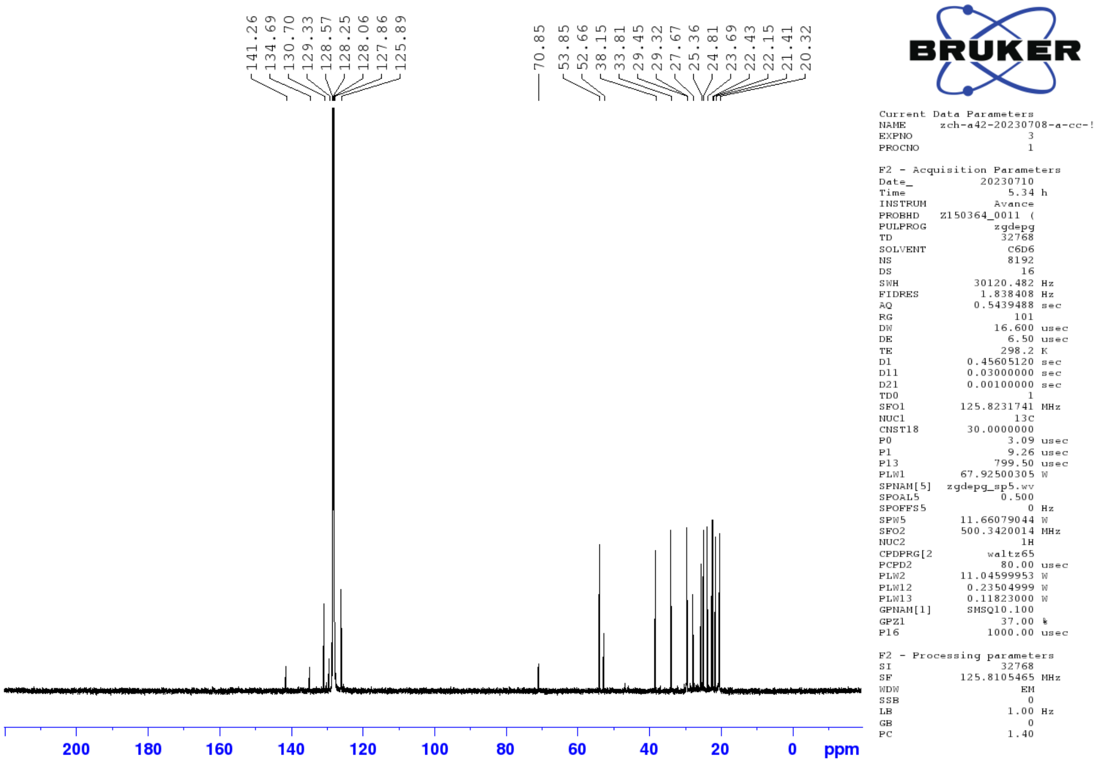

Figure S39. ^{13}C{^{1}H} NMR spectrum of **8** in C_6D_6.

Heating 7 in DCM (or *o*-difluorobenzene): in a Schlenk tube, **7** (172 mg, 0.29 mmol) was dissolved in DCM (10 mL). The solution was stirred and heated at 50 °C overnight. All volatiles in the solution were removed under reduced pressure, and the remaining solids

were recrystallized in DCM/hexanes at –35 ºC, affording compound **11** as a white solid (87.1 mg, 52%). This reaction was also conducted using *o*-difluorobenzene as solvent instead of DCM. The reaction was heated at 85 ºC for 2 days, and the isolated yield improved from 52% to 73%.

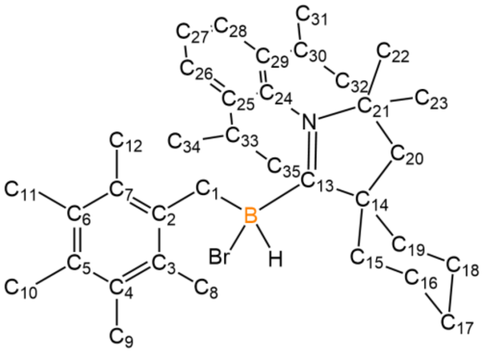

^{1}H NMR (500 MHz, C_6D_6): 0.80 (br. s, 3H, $C_{22}\underline{H}_3$ or $C_{23}\underline{H}_3$), 1.01 (br. s, 3H, $C_{22}\underline{H}_3$ or $C_{23}\underline{H}_3$), 1.08 (6H, $C_{31}\underline{H}_3$ and $C_{32}\underline{H}_3$, or $C_{34}\underline{H}_3$ and $C_{35}\underline{H}_3$), 1.33 (6H, $C_{31}\underline{H}_3$ and $C_{32}\underline{H}_3$, or $C_{34}\underline{H}_3$ and $C_{35}\underline{H}_3$), 1.59 (m, 1H, $C_{20}\underline{H}$), 1.77 (m, 1H, $C_{20}\underline{H}$), 2.16 (s, 3H, $C_{10}\underline{H}_3$), 2.19 (6H, $C_8\underline{H}_3$ and $C_{12}\underline{H}_3$, or $C_9\underline{H}_3$ and $C_{11}\underline{H}_3$), 2.16 (s, 3H, $C_{10}\underline{H}_3$), 2.19 (1H, $C_1\underline{H}$), 2.56 (1H, $C_1\underline{H}$), 2.57 (s, 6H, $C_8\underline{H}_3$ and $C_{12}\underline{H}_3$, or $C_9\underline{H}_3$ and $C_{11}\underline{H}_3$), 2.69 (m, 1H, $C_{30}\underline{H}$ or $C_{34}\underline{H}$), 3.19 (m, 1H, $C_{30}\underline{H}$ or $C_{34}\underline{H}$), 7.00 (m, 1H, $C_{26}\underline{H}$ or $C_{27}\underline{H}$ or $C_{28}\underline{H}$), 7.02 (m, 1H, $C_{26}\underline{H}$ or $C_{27}\underline{H}$ or $C_{28}\underline{H}$), 7.08 (m, 1H, $C_{26}\underline{H}$ or $C_{27}\underline{H}$ or $C_{28}\underline{H}$), $C_{15}\underline{H}_2$-$C_{19}\underline{H}_2$: 1.22 (br. m), 1.47 (br. m), 1.60 (br. m), 1.76 (br. m), 2.33 (br. m), 2.42 (br. m), 2.53 (br. m), 3.03 (br. m), 3.12 (br. m); **^{1}H{^{11}B} NMR** (400 MHz, C_6D_6): 0.80 (br. s, 3H, $C_{22}\underline{H}_3$ or $C_{23}\underline{H}_3$), 1.01 (br. s, 3H, $C_{22}\underline{H}_3$ or $C_{23}\underline{H}_3$), 1.08 (6H, $C_{31}\underline{H}_3$ and $C_{32}\underline{H}_3$, or $C_{34}\underline{H}_3$ and $C_{35}\underline{H}_3$), 1.33 (6H, $C_{31}\underline{H}_3$ and $C_{32}\underline{H}_3$, or $C_{34}\underline{H}_3$ and $C_{35}\underline{H}_3$), 1.59 (m, 1H, $C_{20}\underline{H}$), 1.77 (m, 1H, $C_{20}\underline{H}$), 2.16 (s, 3H, $C_{10}\underline{H}_3$), 2.19 (6H, $C_8\underline{H}_3$ and $C_{12}\underline{H}_3$, or $C_9\underline{H}_3$ and $C_{11}\underline{H}_3$), 2.16 (s, 3H, $C_{10}\underline{H}_3$), 2.19 (1H, $C_1\underline{H}$), 2.56 (1H, $C_1\underline{H}$), 2.57 (s, 6H, $C_8\underline{H}_3$ and $C_{12}\underline{H}_3$, or $C_9\underline{H}_3$ and $C_{11}\underline{H}_3$), 2.69 (m, 1H, $C_{30}\underline{H}$ or $C_{34}\underline{H}$), 3.05 (m, 1H, B$\underline{H}$), 3.19 (m, 1H, $C_{30}\underline{H}$ or $C_{34}\underline{H}$), 7.00 (m, 1H, $C_{26}\underline{H}$ or $C_{27}\underline{H}$ or $C_{28}\underline{H}$), 7.02 (m, 1H, $C_{26}\underline{H}$ or $C_{27}\underline{H}$ or $C_{28}\underline{H}$), 7.08 (m, 1H, $C_{26}\underline{H}$ or $C_{27}\underline{H}$ or $C_{28}\underline{H}$), $C_{15}\underline{H}_2$-$C_{19}\underline{H}_2$: 1.22 (br. m), 1.47 (br. m), 1.60 (br. m), 1.76 (br. m), 2.33 (br. m), 2.42 (br. m), 2.53 (br. m), 3.03 (br. m), 3.12 (br. m); **^{11}B NMR** (161 MHz, C_6D_6): –8.03 (s); **^{11}B{^{1}H} NMR** (161 MHz, C_6D_6): –8.06 (s); **^{13}C{^{1}H} NMR** (126 MHz, C_6D_6): 17.10 (s, $\underline{C}_{10}$), 17.40 (s, C_8 and C_{12}, or C_9 and C_{11}), 18.89 (s, C_8 and C_{12}, or C_9 and C_{11}), 17.10 (s, $\underline{C}_{10}$), 23.42 (br. s, C_{31} or C_{32} or C_{34} or C_{35}), 24.77 (br. s, C_{31} or C_{32} or C_{34} or C_{35}), 26.05 (br. s, C_{31} or C_{32} or C_{34} or C_{35}), 26.99 (br. s, $\underline{C}_{22}$ or C_{23}), 28.26 (br. s, C_{31} or C_{32} or C_{34} or C_{35}), 28.87 (br. s, C_{30} or C_{33}), 29.36 (br. s, C_{30} or C_{33}), 30.55 (br. s, $\underline{C}_{22}$ or C_{23}), 45.96 (s, $\underline{C}_{20}$), 59.89 (s, $\underline{C}_{14}$), 77.38 (s, $\underline{C}_{21}$), 124.34 (br. s, C_{26} or C_{27} or C_{28}), 126.15 (br. s, C_{26} or C_{27} or C_{28}), 129.26 (s, C_5), 129.66 (s, C_{26} or C_{27} or C_{28}), 131.39 (s, C_3 and C_7, or C_4 and C_6), 131.83 (s, C_3 and C_7, or C_4 and C_6), 133.89 (s, C_{24} or C_{25} or C_{29}), 139.95 (s, C_1), 144.64 (s, C_{24} or C_{25} or C_{29}), 147.47 (s, C_{24} or C_{25} or C_{29}), C_{15}-C_{19}: 22.49 (br. s), 23.62 (br. s), 25.65 (s), 35.49 (br. s), 39.63 (br. s). (Some assignments of the peaks were accomplished by the assistance of 2D spectra) **HRMS** (m/z): $[M+H]^+$ calcd. for $C_{35}H_{54}BBrN$, 578.35326; found 578.35728. **Elemental analysis**: calcd. for $C_{35}H_{53}BBrN$, C, 72.66; H, 9.23; N, 2.42; found: C, 72.79; H, 9.08; N, 2.17.

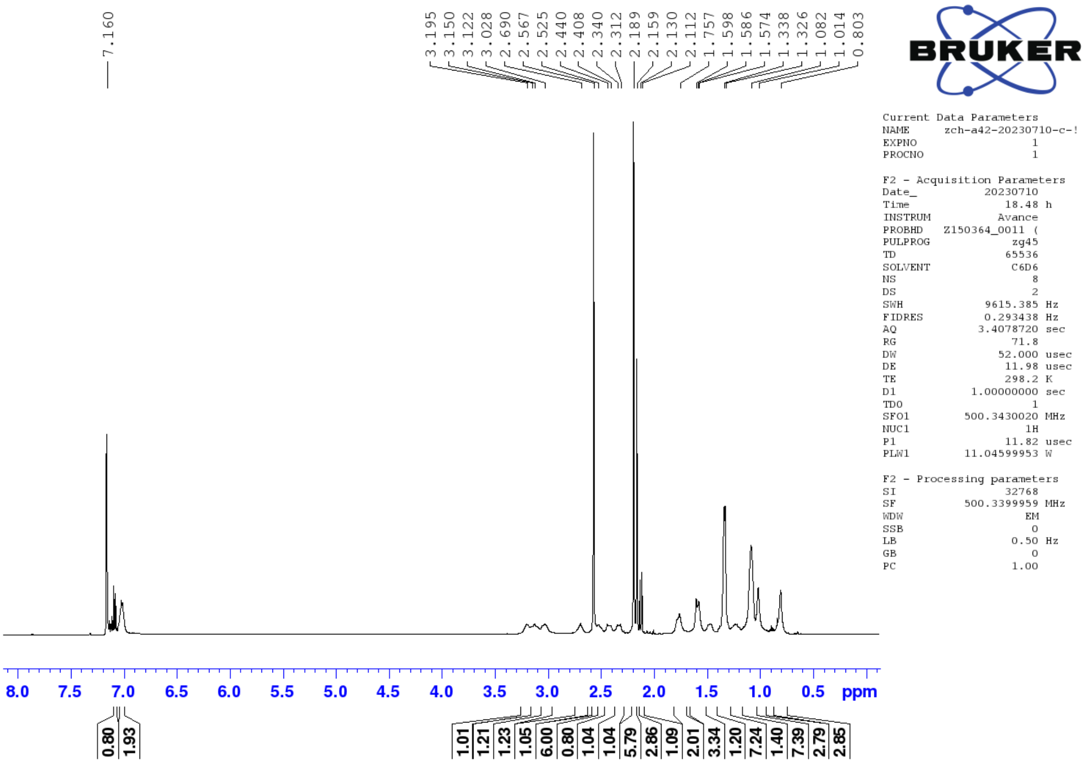

Figure S40. ^{1}H NMR spectrum of **9** in C_6D_6.

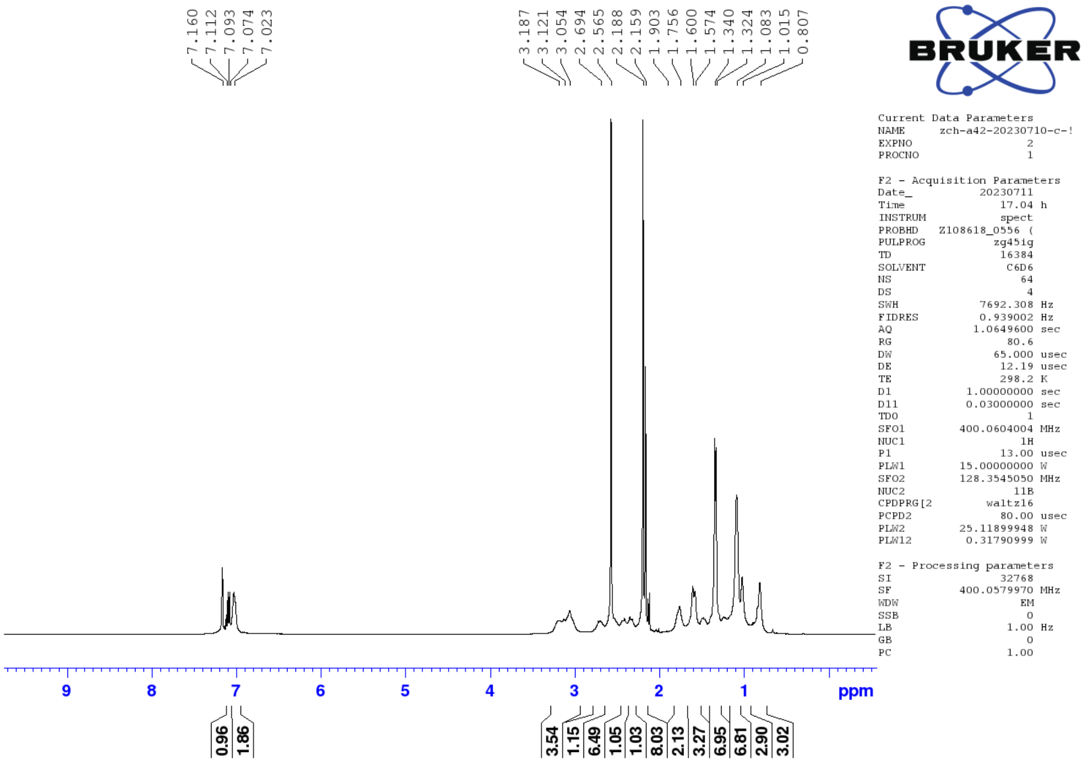

Figure S41. $^{1}H^{9}$ NMR spectrum of **9** in C_6D_6.

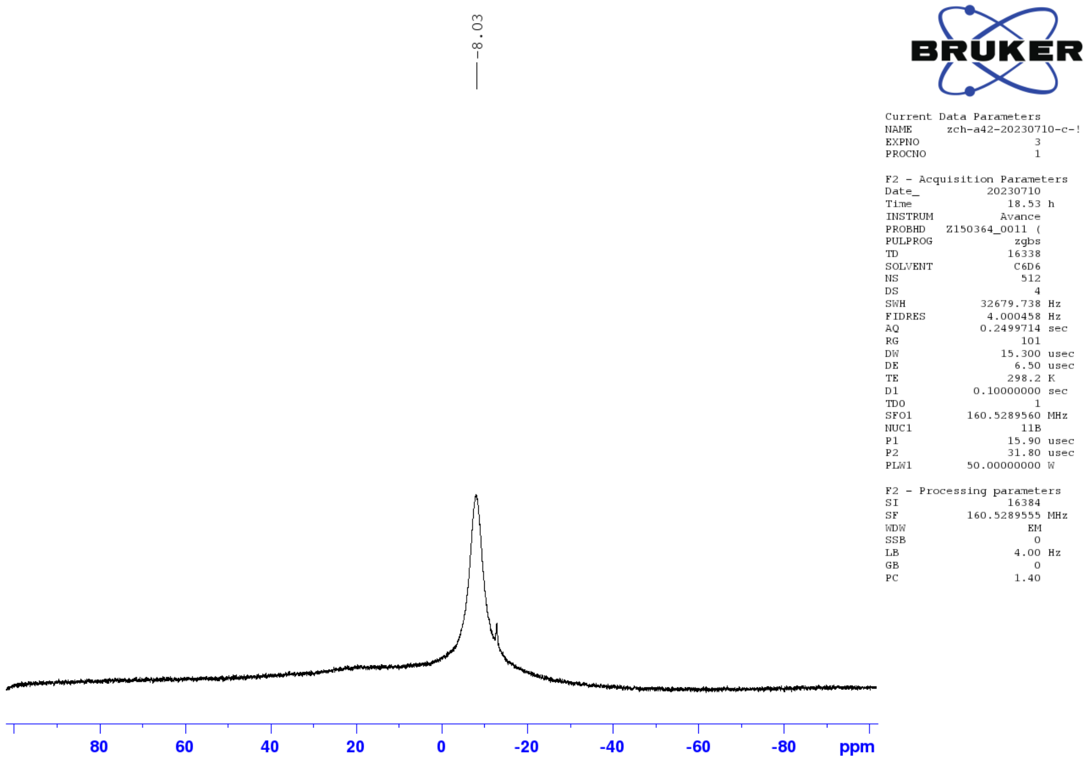

Figure S42. ^{11}B NMR spectrum of **9** in C_6D_6.

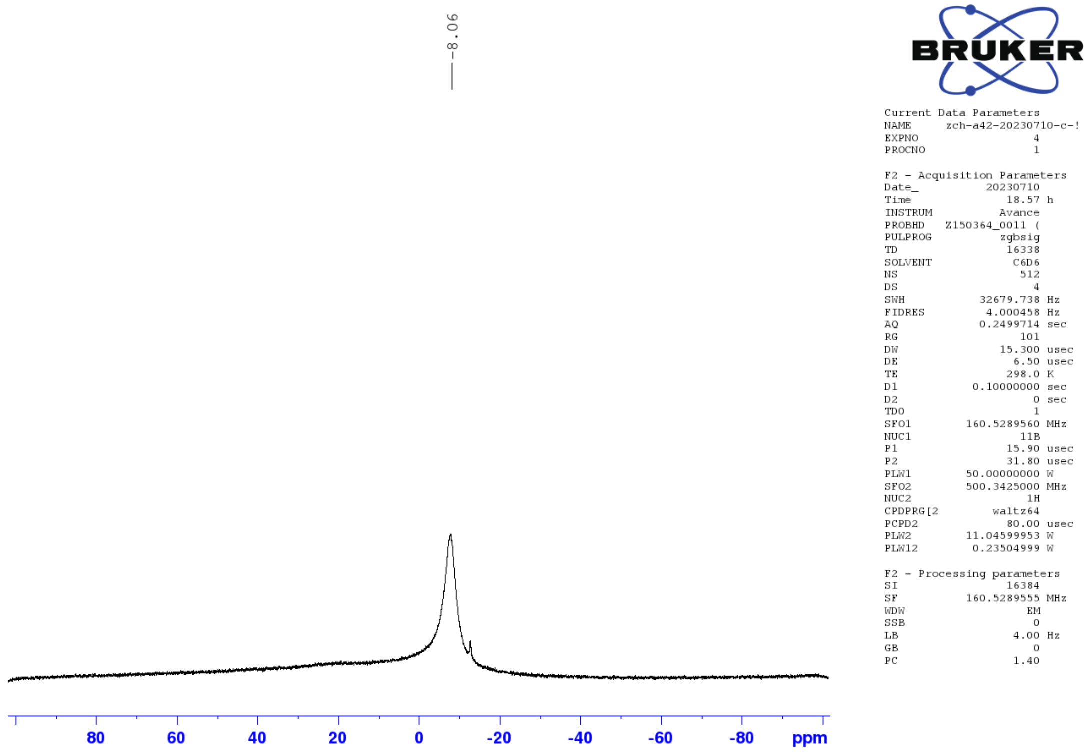

Figure S43. ^{11}B{^{1}H} NMR spectrum of **9** in C_6D_6.

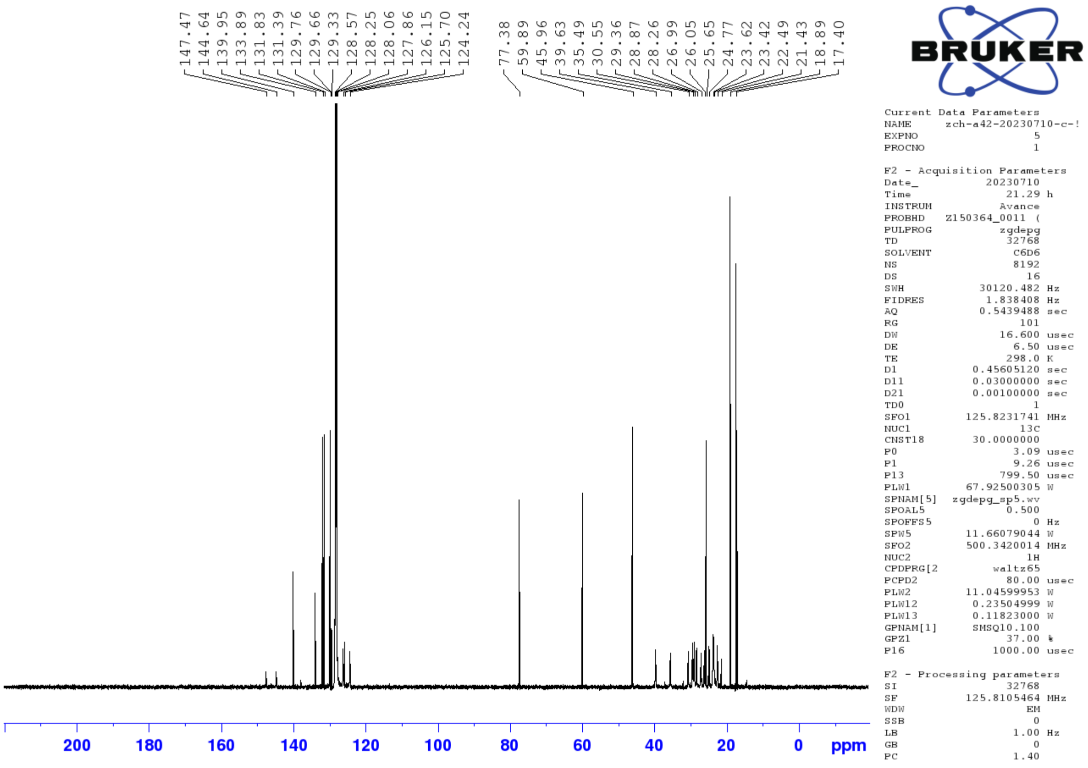

Figure S44. $^{13}C\{^1H\}$ NMR spectrum of **9** in C_6D_6.

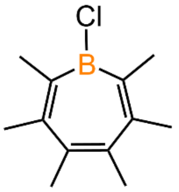

Synthesis of 10^{Cl} (mixed with 2^{Cl}): in a vial, compound $\mathbf{1}^{Cl}$ (15.4 mg, 0.05 mmol, 1.0 equiv) and 2-butyne (27 mg, 0.50 mmol, 10.0 equiv) was dissolved in toluene (2 mL). The solution was stirred at 25 ºC for 2 days. All volatiles in the solution were removed under vacuum to obtain a mixture of $\mathbf{10}^{Cl}$ and $\mathbf{2}^{Cl}$ as an orange oil (1: 0.8, respectively).

^{1}H NMR (500 MHz, C_6D_6): 1.77 (s, 6H, C<u>H</u>$_3$), 1.82 (s, 6H, C<u>H</u>$_3$), 2.26 (s, 6H, C<u>H</u>$_3$); **^{11}B NMR** (161 MHz, C_6D_6): 54.73 (br. s); **^{13}C{^{1}H} NMR** (126 MHz, C_6D_6): 19.56 (s, <u>C</u>H$_3$), 20.38 (s, <u>C</u>H$_3$), 21.51 (s, <u>C</u>H$_3$), 135.90 (s, <u>C</u>–CH$_3$), 152.39 (s, <u>C</u>–CH$_3$).

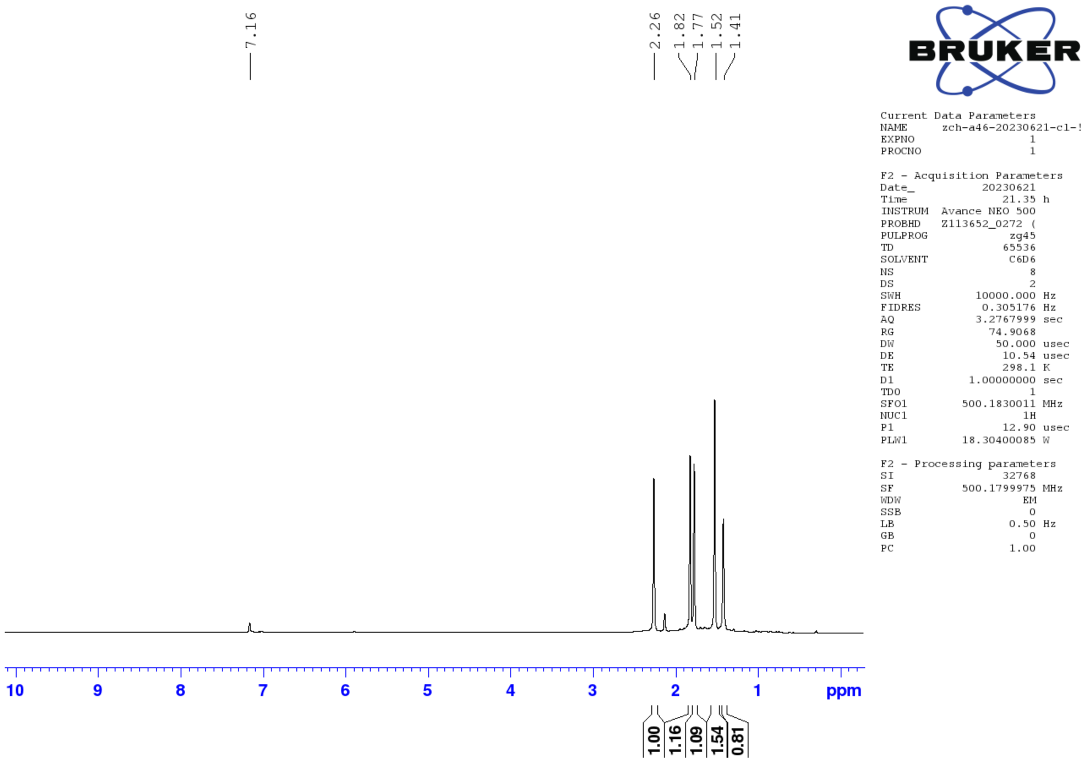

Figure S45. ^{1}H NMR spectrum of **10**^{Cl} mixed with **2**^{Cl} in C_6D_6.

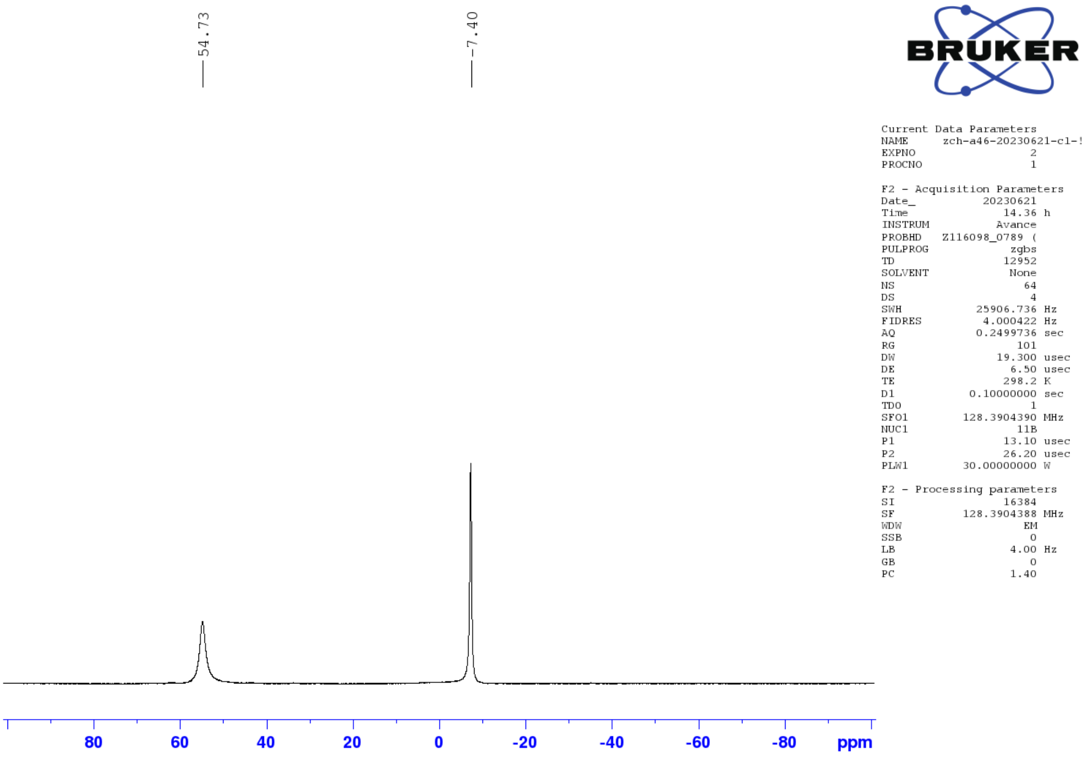

Figure S46. ^{11}B NMR spectrum of **10**^{Cl} mixed with **2**^{Cl} in C_6D_6.

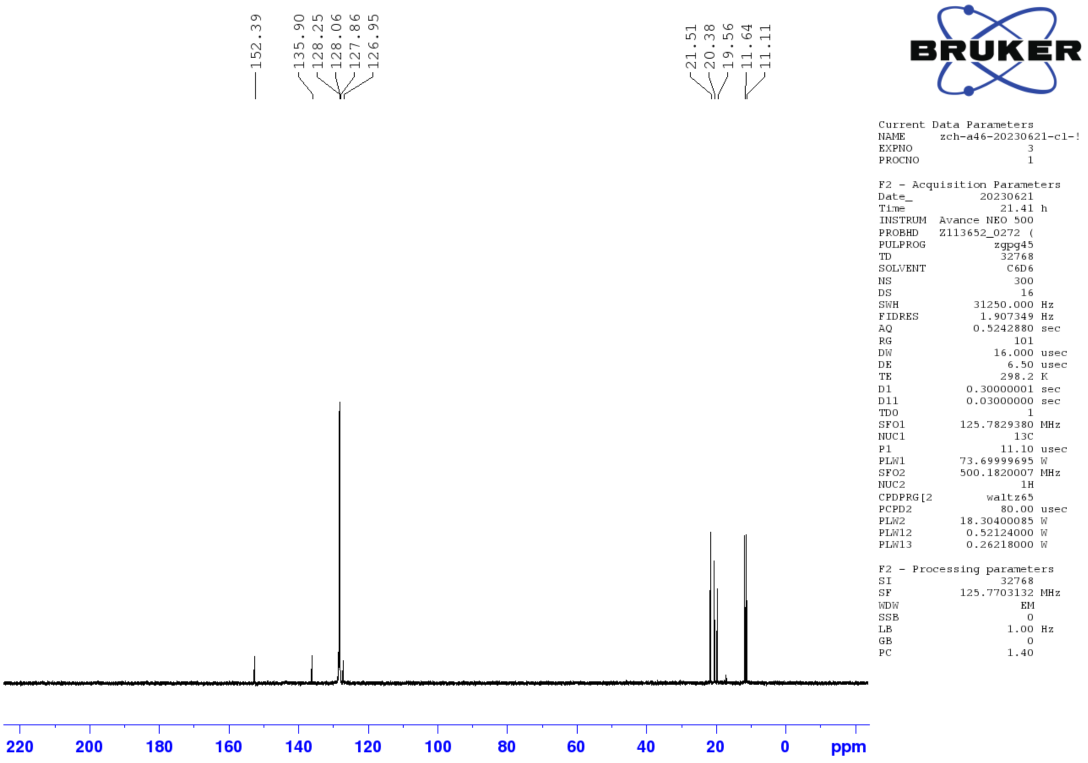

Figure S47. $^{13}C\{^{1}H\}$ NMR spectrum of **10**^{Cl} mixed with **2**^{Cl} in C_6D_6.

Synthesis of 10^{Br} (mixed with 2^{Br}): in a vial, compound **1**^{Br} (19.9 mg, 0.05 mmol, 1.0 equiv) and 2-butyne (27 mg, 0.50 mmol, 10.0 equiv) was dissolved in toluene (2 mL). The solution was stirred at 25 ºC for 2 days. All volatiles in the solution were removed by vacuum to obtain a mixture of **10**^{Br} and **2**^{Br} as an orange oil (1: 1, respectively).

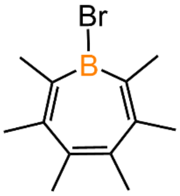

^{1}**H NMR** (500 MHz, C_6D_6): 1.74 (s, 6H, C$\underline{H}_3$), 1.79 (s, 6H, C$\underline{H}_3$), 2.33 (s, 6H, C$\underline{H}_3$); ^{11}**B NMR** (161 MHz, C_6D_6): 57.94 (br. s); ^{13}**C{**^{1}**H} NMR** (126 MHz, C_6D_6): 20.34 (s, $\underline{C}H_3$), 20.96 (s, $\underline{C}H_3$), 21.56 (s, $\underline{C}H_3$), 136.14 (s, $\underline{C}$–CH_3), 151.48 (s, $\underline{C}$–CH_3).

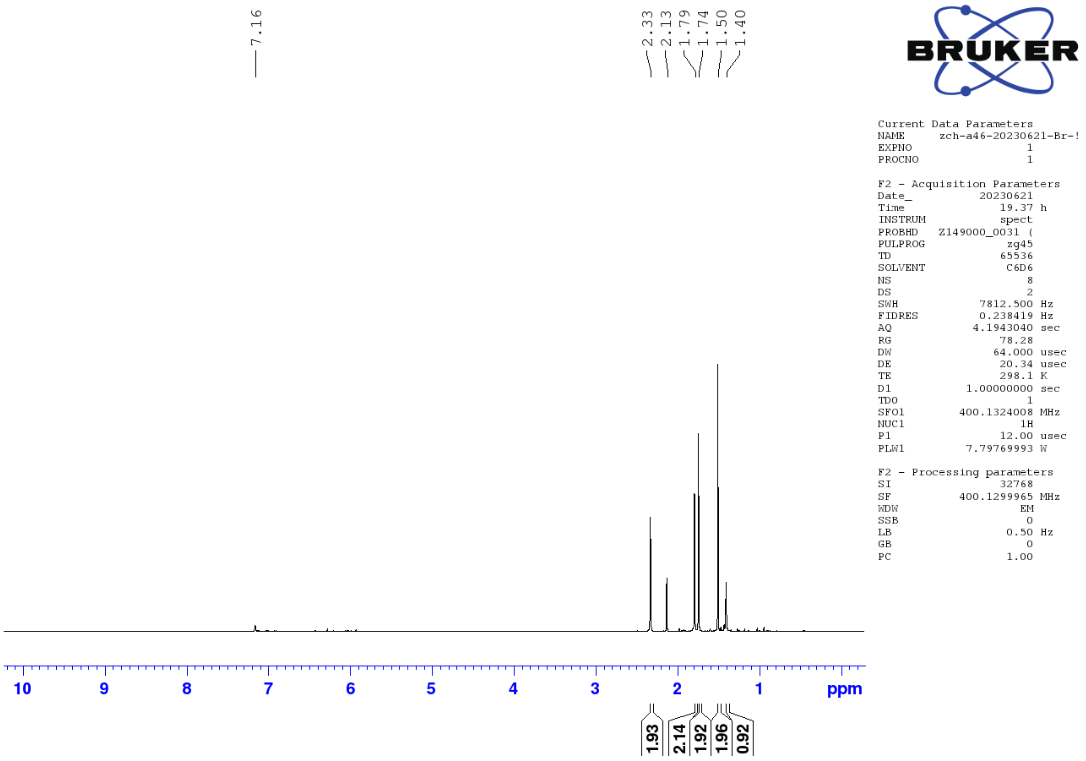

Figure S48. ^{1}H NMR spectrum of **10**^{Br} mixed with **2**^{Br} in C_6D_6.

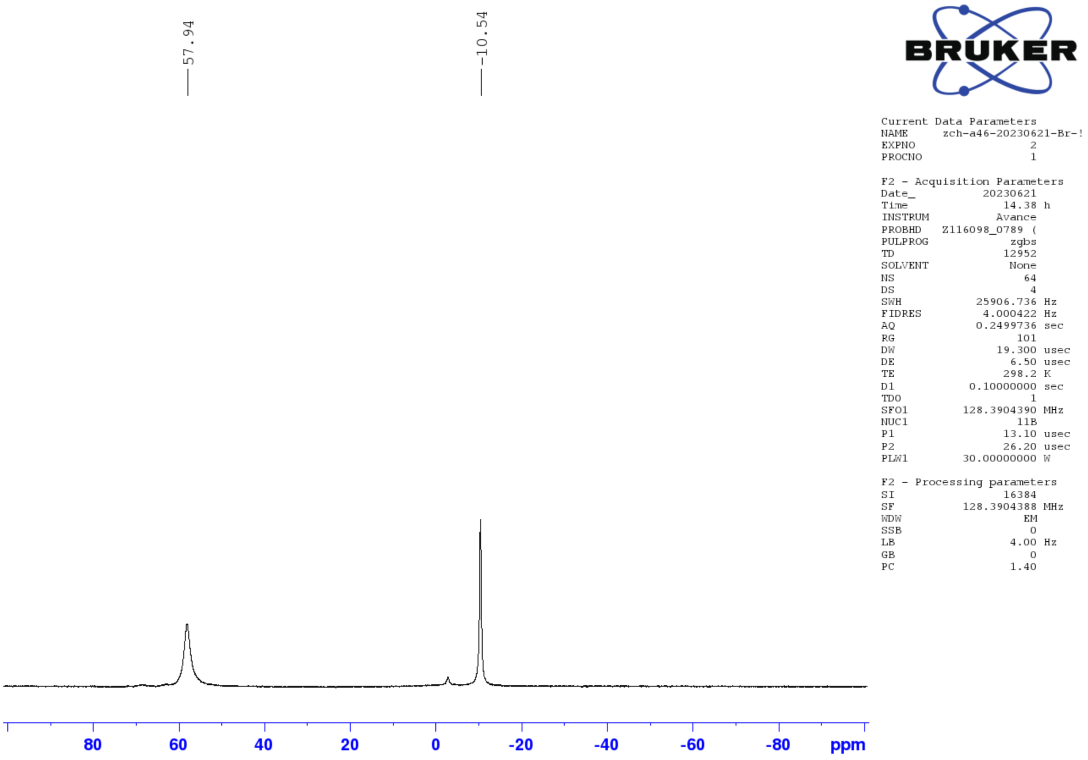

Figure S49. ^{11}B NMR spectrum of **10**^{Br} mixed with **2**^{Br} in C_6D_6.

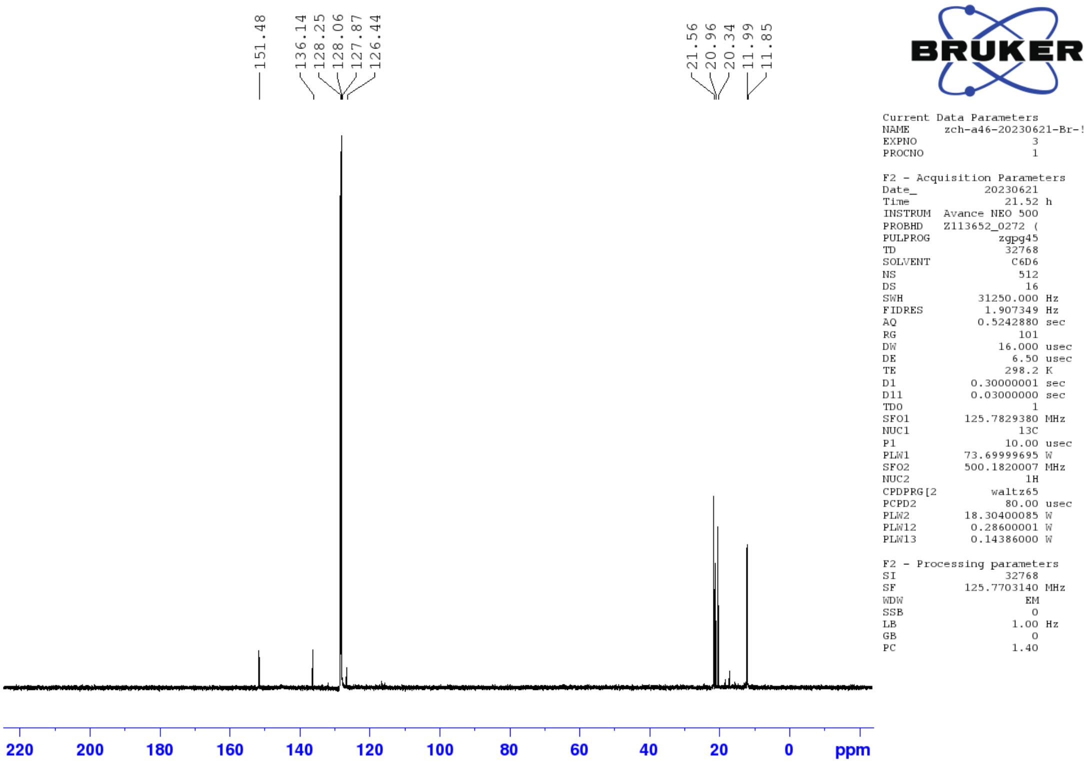

Figure S50. $^{13}C\{^1H\}$ NMR spectrum of **10**^{Br} mixed with **2**^{Br} in C_6D_6.

Crystal structure

Low-temperature (100 K) diffraction data were collected on a Bruker-AXS X8 Kappa Duo diffractometer with IμS micro-sources, coupled to a Photon 3 CPAD detector for all structures. Mo K_{α} radiation (λ = 0.71073 Å) was used for all the structures. Data reduction, scaling and absorption corrections were performed using SAINT (Bruker, V8.38A, 2013). The structure was solved with the XT structure solution program using the Intrinsic Phasing solution method[10] and by using Olex2[11] as the graphical interface. The model was refined with the ShelXL program[12] using Least Squares minimization. All non-hydrogen atoms were refined anisotropically. Hydrogen atoms were included in structure factor calculations. All hydrogen atoms were assigned to idealized geometric positions.

Crystallographic data have been deposited with the Cambridge Crystallographic Data as supplementary publication nos. CCDC-2297451 (**1**$^{\mathbf{Cl}}$), 2297452 (**2**$^{\mathbf{Ph}}$), 2297453 (**3**), 2297454 (**4**), 2297455 (**5**$^{\mathbf{Cl}}$), 2297456 (**5**$^{\mathbf{Br}}$), 2297457 (**6**), 2297458 (**7**), 2297459 (**8**), 2297460 (**9**). These data can be obtained free of charge from The Cambridge Crystallographic Data Centre via Data https://www.ccdc.cam.ac.uk.

Table S1 Crystal data and structure refinement for **1**^{Cl}.

Identification code	2297451
Empirical formula	C16H24B2Cl2
Formula weight	308.87
Temperature/K	100.00
Crystal system	monoclinic
Space group	P21
a/Å	8.5963(5)
b/Å	14.7551(8)
c/Å	13.3230(7)
α/°	90
β/°	94.308(2)
γ/°	90
Volume/Å3	1685.11(16)
Z	4
ρcalcg/cm3	1.217
μ/mm-1	0.372
F(000)	656.0
Crystal size/mm3	0.087 × 0.067 × 0.012
Radiation	MoKα (λ = 0.71073)
2Θ range for data collection/°	3.066 to 55.136
Index ranges	-12 ≤ h ≤ 12, -21 ≤ k ≤ 21, -19 ≤ l ≤ 19
Reflections collected	4036
Independent reflections	4036 [Rint = 0.0408, Rsigma = 0.0304]

Data/restraints/parameters	4036/1/377
Goodness-of-fit on F2	1.082
Final R indexes [I>=2σ (I)]	R1 = 0.0545, wR2 = 0.1415
Final R indexes [all data]	R1 = 0.0675, wR2 = 0.1558
Largest diff. peak/hole / e Å-3	0.83/-0.59

Table S2 Crystal data and structure refinement for **2**^{Ph}.

Identification code	2297452
Empirical formula	C18H23B
Formula weight	250.17
Temperature/K	100.00
Crystal system	monoclinic
Space group	P21/c
a/Å	8.7528(3)
b/Å	10.9747(3)
c/Å	15.4186(6)
α/°	90
β/°	91.674(2)
γ/°	90
Volume/Å3	1480.47(9)
Z	4
ρcalcg/cm3	1.122
μ/mm-1	0.062
F(000)	544.0
Crystal size/mm3	0.067 × 0.045 × 0.023
Radiation	MoKα (λ = 0.71073)
2Θ range for data collection/°	4.556 to 55.2
Index ranges	-11 ≤ h ≤ 11, -14 ≤ k ≤ 14, -20 ≤ l ≤ 20
Reflections collected	36806
Independent reflections	3433 [Rint = 0.1677, Rsigma = 0.0539]
Data/restraints/parameters	3433/0/178
Goodness-of-fit on F2	1.066
Final R indexes [I>=2σ (I)]	R1 = 0.0611, wR2 = 0.1507
Final R indexes [all data]	R1 = 0.0663, wR2 = 0.1565
Largest diff. peak/hole / e Å-3	0.48/-0.31

Table S3 Crystal data and structure refinement for **3**.

Identification code	2297453
Empirical formula	C22H36BClN
Formula weight	360.78

Temperature/K	100.00
Crystal system	monoclinic
Space group	P21
a/Å	9.2417(5)
b/Å	10.0889(6)
c/Å	11.8541(8)
α/°	90
β/°	110.192(3)
γ/°	90
Volume/Å3	1037.33(11)
Z	2
ρcalcg/cm3	1.155
μ/mm-1	0.189
F(000)	394.0
Crystal size/mm3	0.065 × 0.056 × 0.012
Radiation	MoKα (λ = 0.71073)
2Θ range for data collection/°	3.66 to 62.282
Index ranges	-12 ⩽ h ⩽ 13, -14 ⩽ k ⩽ 14, -17 ⩽ l ⩽ 17
Reflections collected	59900
Independent reflections	6660 [Rint = 0.0395, Rsigma = 0.0278]
Data/restraints/parameters	6660/1/233
Goodness-of-fit on F2	1.053
Final R indexes [I>=2σ (I)]	R1 = 0.0482, wR2 = 0.1274
Final R indexes [all data]	R1 = 0.0533, wR2 = 0.1303
Largest diff. peak/hole / e Å-3	1.50/-0.52

Table S4 Crystal data and structure refinement for **4**.

Identification code	2297454
Empirical formula	C44H70BClN2
Formula weight	673.28
Temperature/K	100.00
Crystal system	monoclinic
Space group	P21/c
a/Å	11.6102(4)
b/Å	20.6129(8)
c/Å	16.4977(5)
α/°	90
β/°	95.3990(10)
γ/°	90
Volume/Å3	3930.7(2)

Z	4
ρcalcg/cm3	1.138
μ/mm-1	0.130
F(000)	1480.0
Crystal size/mm3	0.054 × 0.036 × 0.028
Radiation	MoKα (λ = 0.71073)
2Θ range for data collection/°	3.17 to 62.22
Index ranges	-16 ⩽ h ⩽ 16, -29 ⩽ k ⩽ 29, -23 ⩽ l ⩽ 20
Reflections collected	333778
Independent reflections	12596 [Rint = 0.1014, Rsigma = 0.0292]
Data/restraints/parameters	12596/0/448
Goodness-of-fit on F2	1.070
Final R indexes [I>=2σ (I)]	R1 = 0.0371, wR2 = 0.1012
Final R indexes [all data]	R1 = 0.0478, wR2 = 0.1047
Largest diff. peak/hole / e Å-3	0.46/-0.27

Table S5 Crystal data and structure refinement for **5**^{Cl}.

Identification code	2297455
Empirical formula	C23H35BClN
Formula weight	371.78
Temperature/K	100.00
Crystal system	orthorhombic
Space group	Pbca
a/Å	13.334
b/Å	12.104
c/Å	25.993
α/°	90
β/°	90.02
γ/°	90
Volume/Å3	4195.0
Z	8
ρcalcg/cm3	1.177
μ/mm-1	0.189
F(000)	1616.0
Crystal size/mm3	0.119 × 0.111 × 0.039
Radiation	MoKα (λ = 0.71073)
2Θ range for data collection/°	3.134 to 68.742
Index ranges	-17 ⩽ h ⩽ 17, -19 ⩽ k ⩽ 19, -37 ⩽ l ⩽ 37
Reflections collected	276364
Independent reflections	6699 [Rint = 0.0396, Rsigma = 0.0100]

Data/restraints/parameters	6699/0/240
Goodness-of-fit on F2	1.092
Final R indexes [I>=2σ (I)]	R1 = 0.0438, wR2 = 0.1292
Final R indexes [all data]	R1 = 0.0470, wR2 = 0.1316
Largest diff. peak/hole / e Å-3	0.73/-0.43

Table S6 Crystal data and structure refinement for **5**^{Br}.

Identification code	2297456
Empirical formula	C23H35BBrN
Formula weight	416.24
Temperature/K	100.00
Crystal system	orthorhombic
Space group	Pbca
a/Å	12.1571(5)
b/Å	13.5618(7)
c/Å	25.8390(13)
α/°	90
β/°	90
γ/°	90
Volume/Å3	4260.1(4)
Z	8
ρcalcg/cm3	1.298
μ/mm-1	1.937
F(000)	1760.0
Crystal size/mm3	0.042 × 0.033 × 0.023
Radiation	MoKα (λ = 0.71073)
2Θ range for data collection/°	3.152 to 49.996
Index ranges	-14 ≤ h ≤ 14, -16 ≤ k ≤ 16, -30 ≤ l ≤ 30
Reflections collected	217441
Independent reflections	3751 [Rint = 0.0404, Rsigma = 0.0084]
Data/restraints/parameters	3751/1/244
Goodness-of-fit on F2	1.093
Final R indexes [I>=2σ (I)]	R1 = 0.0421, wR2 = 0.1000
Final R indexes [all data]	R1 = 0.0435, wR2 = 0.1006
Largest diff. peak/hole / e Å-3	1.87/-0.75

Table S7 Crystal data and structure refinement for **6**.

Identification code	2297457
Empirical formula	C36H48BCl7N2
Formula weight	767.72

Temperature/K	100.00
Crystal system	triclinic
Space group	P-1
a/Å	11.7206(8)
b/Å	12.0045(8)
c/Å	15.0853(10)
α/°	108.946(3)
β/°	93.723(2)
γ/°	95.092(3)
Volume/Å3	1989.7(2)
Z	2
ρcalcg/cm3	1.281
μ/mm-1	0.526
F(000)	804.0
Crystal size/mm3	0.122 × 0.083 × 0.023
Radiation	MoKα (λ = 0.71073)
2Θ range for data collection/°	2.868 to 62.14
Index ranges	-17 ≤ h ≤ 16, -17 ≤ k ≤ 17, -21 ≤ l ≤ 21
Reflections collected	28466
Independent reflections	12481 [Rint = 0.0225, Rsigma = 0.0293]
Data/restraints/parameters	12481/0/427
Goodness-of-fit on F2	1.032
Final R indexes [I>=2σ (I)]	R1 = 0.0400, wR2 = 0.0982
Final R indexes [all data]	R1 = 0.0462, wR2 = 0.1031
Largest diff. peak/hole / e Å-3	1.70/-1.45

Table S8 Crystal data and structure refinement for **7**.

Identification code	2297458
Empirical formula	C35H53BBrN
Formula weight	578.50
Temperature/K	100.00
Crystal system	monoclinic
Space group	P21/c
a/Å	10.810(11)
b/Å	12.282(8)
c/Å	23.47(3)
α/°	90
β/°	93.75(5)
γ/°	90
Volume/Å3	3110(5)

Z	4
ρcalcg/cm3	1.236
μ/mm-1	1.346
F(000)	1240.0
Crystal size/mm3	0.091 × 0.054 × 0.023
Radiation	MoKα (λ = 0.71073)
2Θ range for data collection/°	3.478 to 62.138
Index ranges	-15 ≤ h ≤ 15, -17 ≤ k ≤ 17, -34 ≤ l ≤ 33
Reflections collected	207305
Independent reflections	9960 [Rint = 0.0401, Rsigma = 0.0154]
Data/restraints/parameters	9960/0/355
Goodness-of-fit on F2	1.100
Final R indexes [I>=2σ (I)]	R1 = 0.0289, wR2 = 0.0800
Final R indexes [all data]	R1 = 0.0345, wR2 = 0.0822
Largest diff. peak/hole / e Å-3	0.95/-0.29

Table S9 Crystal data and structure refinement for **8**.

Identification code	2297459
Empirical formula	C23H35BBrN
Formula weight	416.24
Temperature/K	100.00
Crystal system	triclinic
Space group	P-1
a/Å	8.7428(7)
b/Å	9.4037(8)
c/Å	13.3258(11)
α/°	80.686(3)
β/°	87.975(3)
γ/°	80.672(3)
Volume/Å3	1066.79(15)
Z	2
ρcalcg/cm3	1.296
μ/mm-1	1.933
F(000)	440.0
Crystal size/mm3	0.102 × 0.098 × 0.021
Radiation	MoKα (λ = 0.71073)
2Θ range for data collection/°	3.098 to 62.768
Index ranges	-12 ≤ h ≤ 12, -13 ≤ k ≤ 13, -19 ≤ l ≤ 19
Reflections collected	70071
Independent reflections	6941 [Rint = 0.0428, Rsigma = 0.0207]

Data/restraints/parameters	6941/0/241
Goodness-of-fit on F2	1.073
Final R indexes [I>=2σ (I)]	R1 = 0.0242, wR2 = 0.0646
Final R indexes [all data]	R1 = 0.0283, wR2 = 0.0660
Largest diff. peak/hole / e Å-3	0.68/-0.46

Table S10 Crystal data and structure refinement for **9**.

Identification code	2297460
Empirical formula	C36H55BBrCl2N
Formula weight	663.43
Temperature/K	100.00
Crystal system	monoclinic
Space group	P21/c
a/Å	14.0779(6)
b/Å	12.7663(6)
c/Å	20.0966(10)
α/°	90
β/°	91.539(2)
γ/°	90
Volume/Å3	3610.5(3)
Z	4
ρcalcg/cm3	1.220
μ/mm-1	1.311
F(000)	1408.0
Crystal size/mm3	0.12 × 0.09 × 0.03
Radiation	MoKα (λ = 0.71073)
2Θ range for data collection/°	2.894 to 64.25
Index ranges	-19 ≤ h ≤ 21, -19 ≤ k ≤ 19, -30 ≤ l ≤ 30
Reflections collected	299895
Independent reflections	12617 [Rint = 0.0347, Rsigma = 0.0118]
Data/restraints/parameters	12617/4/391
Goodness-of-fit on F2	1.064
Final R indexes [I>=2σ (I)]	R1 = 0.0616, wR2 = 0.2118
Final R indexes [all data]	R1 = 0.0679, wR2 = 0.2195
Largest diff. peak/hole / e Å-3	1.49/-2.43

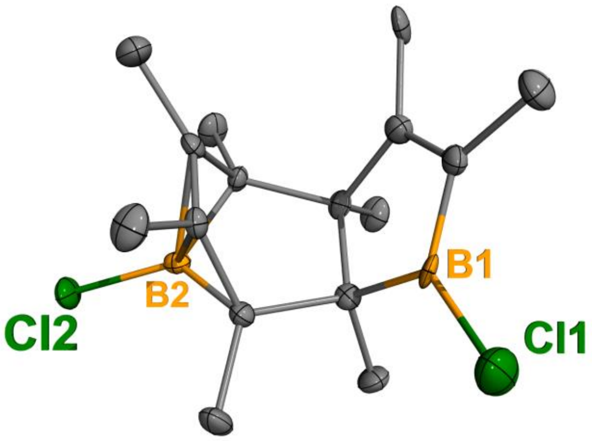

Figure S51. Single crystal structure of $\mathbf{1}^{Cl}$. Hydrogen atoms have been omitted for clarity. Thermal ellipsoids are drawn at the 50 % probability level.

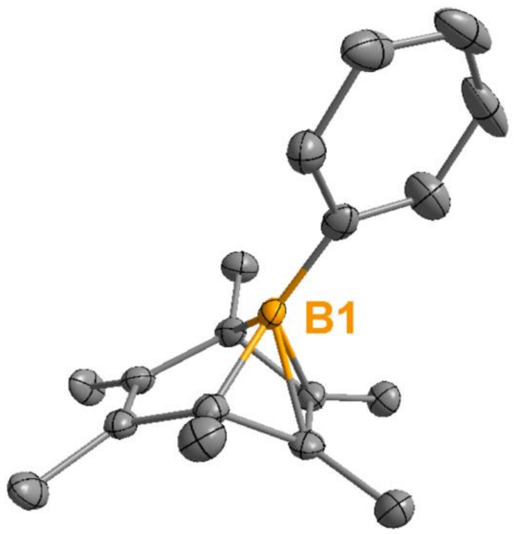

Figure S52. Single crystal structure of $\mathbf{2}^{Ph}$. Hydrogen atoms have been omitted for clarity. Thermal ellipsoids are drawn at the 50 % probability level.

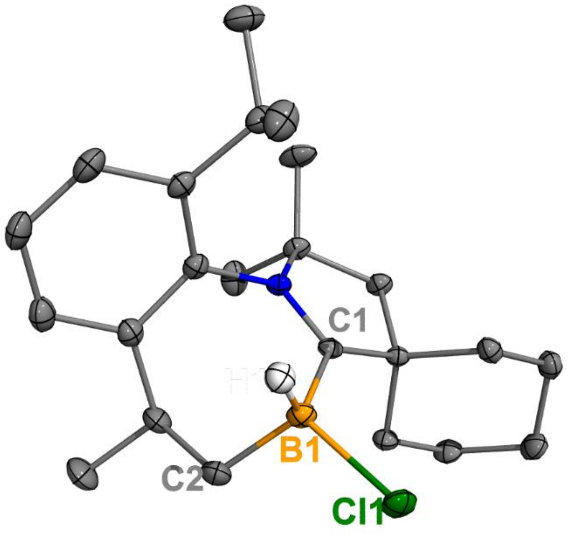

Figure S53. Single crystal structure of 5^{Cl}. Hydrogen atoms, except for the one connected to B1, have been omitted for clarity. Thermal ellipsoids are drawn at the 50 % probability level.

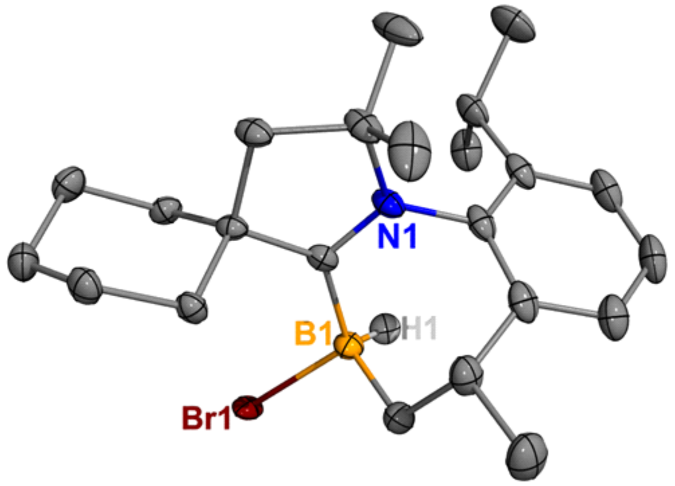

Figure S54. Single crystal structure of 5^{Br}. Hydrogen atoms, except for the one connected to B1, have been omitted for clarity. Thermal ellipsoids are drawn at the 50 % probability level.

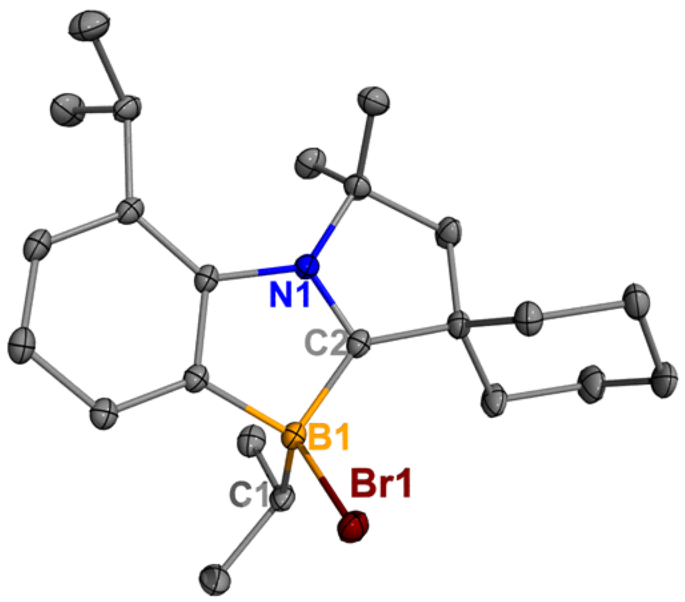

Figure S55. Single crystal structure of **8**. Hydrogen atoms have been omitted for clarity. Thermal ellipsoids are drawn at the 50 % probability level.

Computational Studies

Unless otherwise indicated, all calculations were performed with Gaussian 09 program[13] and ORCA 5.0.3 program[14]. All geometry optimizations and frequency calculation were performed at ωB97xD/6-311G** level of theory[15-19] with Gaussian 09. Frequency calculations were performed to confirm a transition state has only one imaginary frequency, while a local minimum has no imaginary frequency. Intrinsic reaction coordinate (IRC) calculations[20, 21] were also carried out to further confirm that transition states can link the relevant local minima. Single point energies were calculated at ωB97M-V/def2-QZVPP level of theory[22-24] with ORCA 5.0.3. In all cases, computed electronic energies were corrected for thermal energy to obtain the corresponding free energy (all free energies reported at 298.15 K within this SI). All geometry optimizations, frequency calculations, and electronic energy calculations applied the SMD solvation module.[25] Static electron-correlation effects of I to IV and TS_{SM-I} to TS_{III-IV} were measured and their FOD plots are displayed in Figure S56.[26] All of the intermediates or transition states suggested either no visible FOD or relatively localized FOD. Cartesian coordinates from all structures are compiled in xyz_all.xyz file which could be found at https://pubs.acs.org.

Table S11. Computed energies for singlet and triplet borylenes. Computed single-point energies and total Gibbs free energies (G) (Hartree) at the level of ωB97xD/6-311G**, relative electronic energies (ΔE) (kcal/mol), relative Gibbs free energies (ΔG_T) (kcal/mol).

	E_h	ΔE	G_T	ΔG_T
IV (singlet)	-1437.316571		-1436.828204	
IV (triplet)	-1437.310514	3.81591	-1436.825355	1.79487
IV-Br (singlet)	-3551.28128		-3550.794479	
IV-Br (triplet)	-3551.275085	3.90285	-3550.788872	3.53241

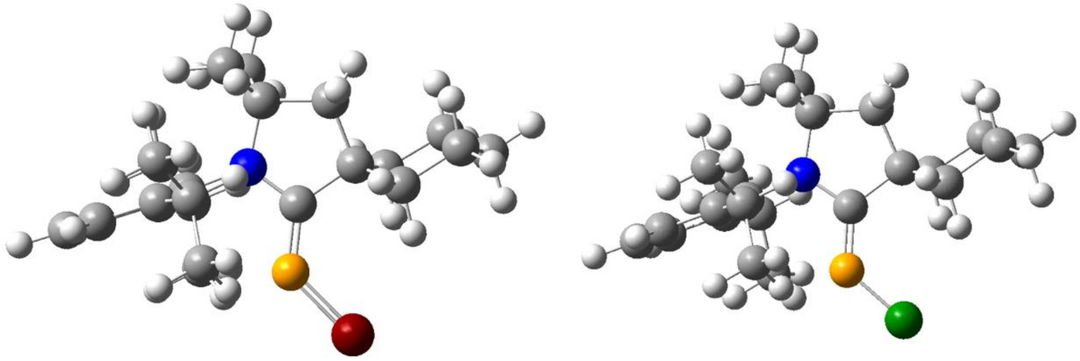

Figure S56. Optimized structure of triplet $\mathbf{IV}^{Br}$ (left) and **IV** (right).

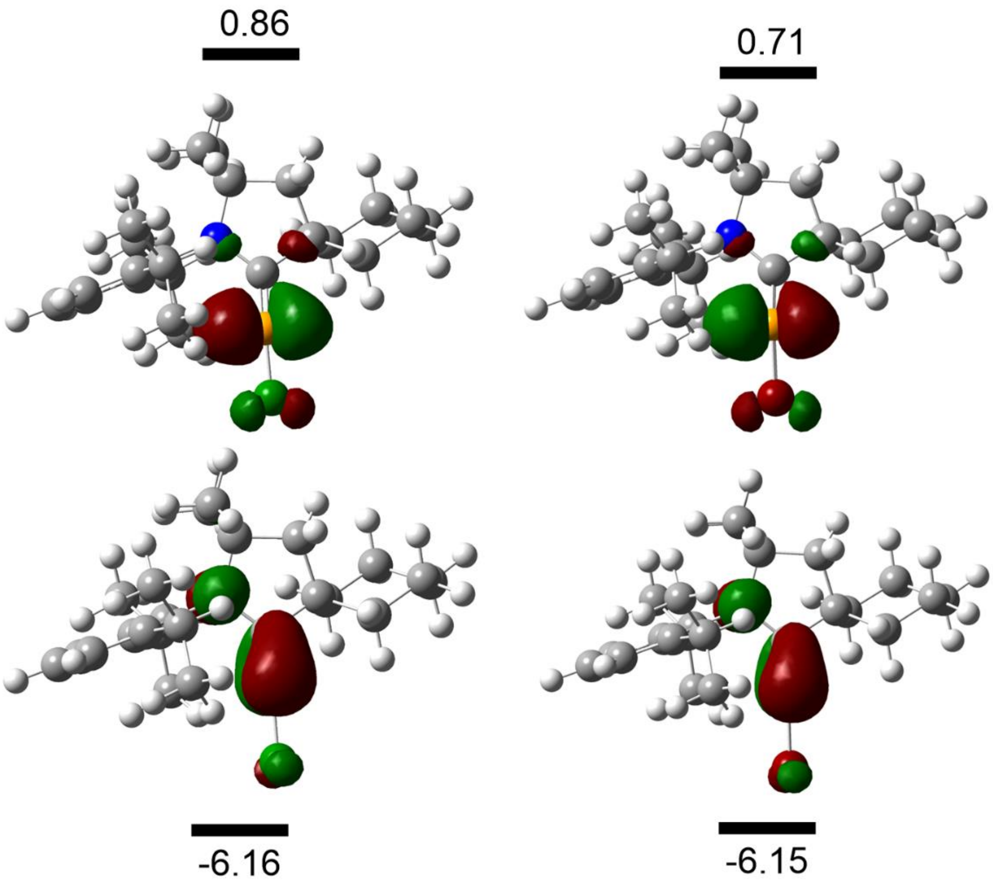

Figure S57. HOMO-LUMO gap of singlet **IV** (left) and **IV**^{Br} (right). The unit of energies is eV and an isovalue of 0.06 (e/Å)$^{3/2}$ was used in the isosurface contour plots.

Table S12. Computed energies for the release of CAAC–chloroborylene in toluene solution. Computed Single-point energies (E_h1, ωB97xD/6-311G**; E_h2 ωB97M-V/def2-QZVPP), Gibbs free-energy corrections (G_c, ωB97xD/6-311G**) (Hartree), total Gibbs free energies (G_T = Eh2 + Gc) (Hartree), relative electronic energies (ΔE) (kcal/mol), relative Gibbs free energies (ΔG_T) (kcal/mol).

	E_h1	G_c	E_h2	G_T	ΔG_T	ΔE
2^{Cl}	-953.191335	0.233547	-953.183658	-952.950111		
CAAC	-952.227229	0.485641	-952.222040	-951.736399	0.000000	0.000000
$TS_{SM\text{-}I}$	-1905.420707	0.745392	-1905.402031	-1904.656639	18.818251	2.309731
I	-1905.449456	0.749715	-1905.430241	-1904.680526	3.769510	-15.462499
$TS_{I\text{-}II}$	-1905.423063	0.748385	-1905.401741	-1904.653356	20.886900	2.492790
II	-1905.463108	0.747583	-1905.434677	-1904.687094	-0.367996	-18.256846
$TS_{II\text{-}III}$	-1905.417885	0.750183	-1905.394023	-1904.643840	26.882125	7.355275
III	-1905.453023	0.753546	-1905.434459	-1904.680913	3.526167	-18.119373
$TS_{III\text{-}IV}$	-1905.41271	0.74725	-1905.396934	-1904.649684	23.200374	5.521314
IV	-1437.316571	0.48837	-1437.307478			
C_6Me_6	-468.112472	0.230473	-468.105126	-1904.693763	-4.569907	-4.350667
$TS_{I\text{-}IV}$	-1905.382373	0.747226	-1905.364224	-1904.616998	43.792420	26.128480

Table S13. Computed energies for the release of CAAC–bromoborylene, C–H activation, and the conversion from **7** to **9** in toluene solution. Computed Single-point energies (E_h1, ωB97xD/6-311G**; E_h2 ωB97M-V/def2-QZVPP), Gibbs free-energy corrections (G_c, ωB97xD/6-311G**) (Hartree), total Gibbs free energies (G_T = Eh2 + Gc) (Hartree), relative electronic energies (ΔE) (kcal/mol), relative Gibbs free energies ($ΔG_T$) (kcal/mol).

	E_h1	G_c	E_h2	G_T	$ΔG_T$	ΔE
9	-4019.484272	0.745854	-4019.358549	-4018.612695	-40.268901	-38.165961
$TS_{IV\text{-}9}^{Br}$	-4019.394271	0.744142	-4019.267937	-4018.523795	15.737879	18.919379
IV^{Br}+C_6Me_6	-4019.393752	0.717274	-4019.268151	-4018.550877	-1.323756	18.784584
$TS_{III\text{-}IV}^{Br}$	-4019.371489	0.744712	-4019.248644	-4018.503932	28.252035	31.074435
III^{Br}	-4019.415646	0.749503	-4019.290004	-4018.540501	5.213521	5.017591
$TS_{7\text{-}III}^{Br}$	-4019.384842	0.748748	-4019.255770	-4018.507022	26.305070	26.584790
7	-4019.430646	0.749192	-4019.297968	-4018.548776	0.000000	0.000000
$TS_{7\text{-}9}^{Br}$	-4019.370316	0.747196	-4019.239857	-4018.492661	35.352771	36.610251
9	-4019.484272	0.745854	-4019.358549	-4018.612695	-40.268901	-38.165961
			C-H activation			
IV^{Br}	-3551.281280	0.486801	-3551.163025	-3550.676224	0.000000	0.000000
$TS_{IV\text{-}5}^{Br}$	-3551.267754	0.487579	-3551.153324	-3550.665745	6.602299	6.112159
5^{Br}	-3551.361558	0.491492	-3551.247131	-3550.755639	-50.031078	-52.986408

Table S14. Computed energies for the release of CAAC–bromoborylene and the conversion from **II**^{Br} to **V**^{Br} in DCM solution. Computed Single-point energies (E_h1, ωB97xD/6-311G**; E_h2 ωB97M-V/def2-QZVPP), Gibbs free-energy corrections (G_c, ωB97xD/6-311G**) (Hartree), total Gibbs free energies (G_T = Eh2 + Gc) (Hartree), relative electronic energies (ΔE) (kcal/mol), relative Gibbs free energies ($ΔG_T$) (kcal/mol).

	E_h1	G_c	E_h2	G_T	$ΔG_T$	ΔE
C_6Me_6	-468.114814	0.230080	-468.106702			
IV^{Br}	-3551.284817	0.486610	-3551.165629	-4018.555641	5.405211	25.456851
$TS_{III\text{-}IV}^{Br}$	-4019.377965	0.744807	-4019.253601	-4018.508794	34.918563	37.256493
III^{Br}	-4019.423730	0.750017	-4019.296721	-4018.546704	11.035483	10.091113
$TS_{7\text{-}III}^{Br}$	-4019.394593	0.746647	-4019.264035	-4018.517388	29.504853	30.683583
7	-4019.446859	0.748518	-4019.312739	-4018.564221	0.000000	0.000000
$TS_{7\text{-}9}^{Br}$	-4019.384695	0.741824	-4019.254550	-4018.512726	32.441932	36.659152
9	-4019.493778	0.744713	-4019.366246	-4018.621533	-36.106610	-33.709460

Table S15. Computed energies for the conversion from borepin **VII**^{Br} to boranorbornardiene **II**^{Br} in toluene solution. Computed Single-point energies (E_h1, ωB97xD/6-311G**; E_h2 ωB97M-V/def2-QZVPP), Gibbs free-energy corrections (G_c, ωB97xD/6-311G**) (Hartree), total Gibbs free energies (G_T = Eh2 + Gc) (Hartree), relative electronic energies (ΔE) (kcal/mol), relative Gibbs free energies ($ΔG_T$) (kcal/mol).

	E_h1	G_c	E_h2	G_T	$ΔG_T$	ΔE
7	-4019.415646	0.749503	-4019.290004	-4018.540501	0.000000	0.000000

$TS_{7\text{-}VII}^{Br}$	-4019.398008	0.749931	-4019.272734	-4018.522803	11.149431	10.879791
VII^{Br}	-4019.404125	0.746333	-4019.279926	-4018.533593	4.351706	6.348806

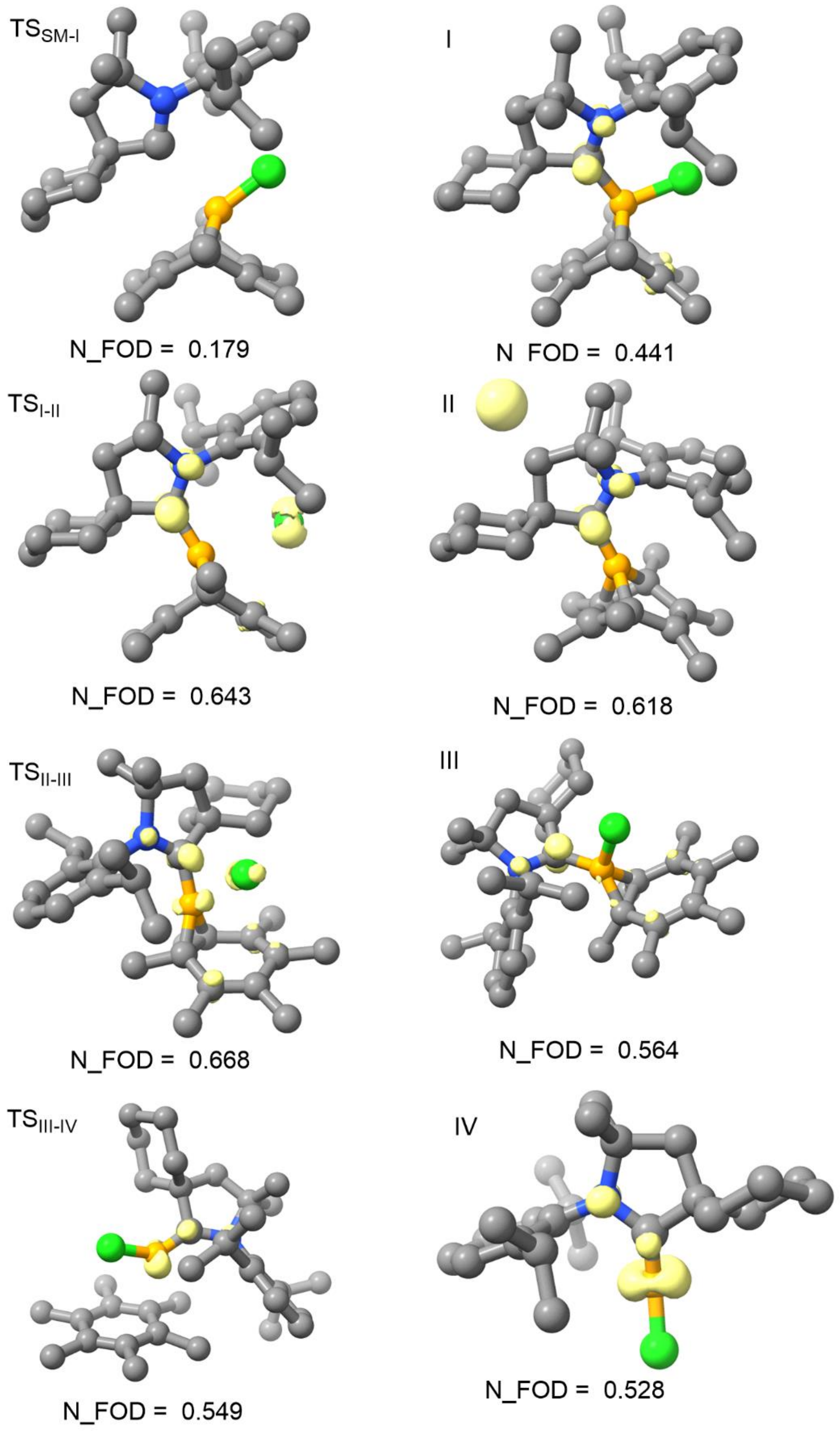

Figure S58. FOD plots at σ=0.005 e Bohr^{-3} for intermediates I, II, III, and IV and transition states $TS_{SM\text{-}I}$, $TS_{I\text{-}II}$, $TS_{II\text{-}III}$, and $TS_{III\text{-}IV}$ (FOD in yellow).

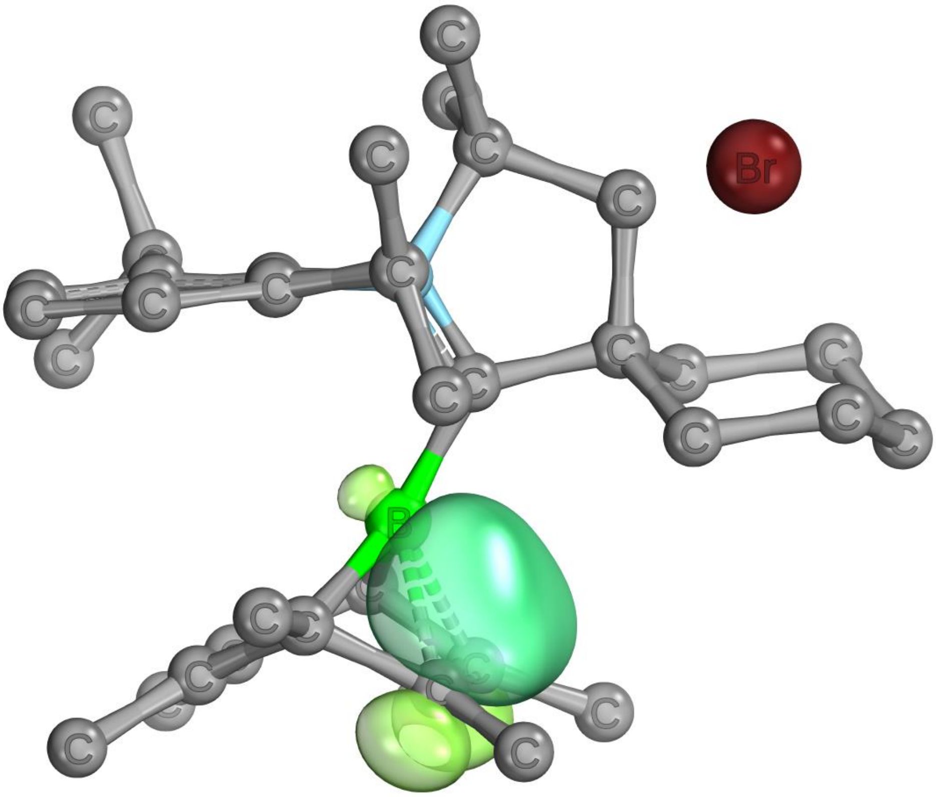

Figure S59. The 3c-2e bond of compound **7** depicted by IBO view.

Discussion on other possible mechanism

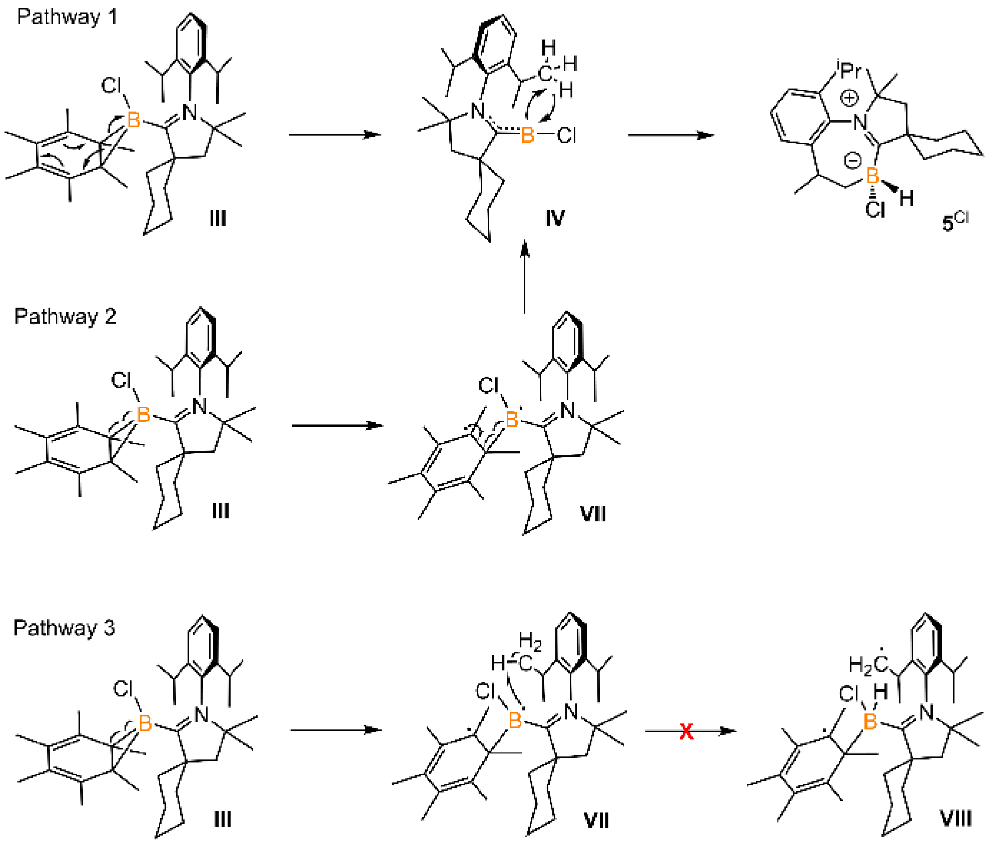

Figure 60. Possible reaction pathways leading to the C–H activation product

In all, there are three possible pathways to the formation of C_6Me_6 and the C–H activation product from boranorcaradiene.

The first pathway is what we proposed and preferred in the manuscript. The borylene-releasing step is a two-electron step. The second pathway still involves the borylene intermediate, while the borylene-releasing step is a stepwise process. The C–B bond undergoes homolysis cleavage, giving a boron radical **VII**. The second C–B bond homolysis occurs and yields the intermediate **IV**. The third pathway does not involve the borylene intermediate. Starting from the boron radical **VII**, it abstracts a hydrogen atom of the isopropyl group and yields a primary carbon radical.

We currently cannot exclude the possibility of the second pathway while we argue that the third pathway is unlikely to be the case. Braunschweig *et al.* isolated a similar chloroborane radical, and the compound showed no reaction to the C–H bond of the CAAC ligand at room temperature.[27] Theoretically, it is hard to yield an unstable primary carbon radical from a stable CAAC-stabilized boron radical.

Reference

1. Pangborn, A. B.; Giardello, M. A.; Grubbs, R. H.; Rosen, R. K.; Timmers, F. J., Safe and Convenient Procedure for Solvent Purification. *Organometallics* **1996,** *15* (5), 1518–1520.
2. Williams, D. B.; Lawton, M., Drying of organic solvents: quantitative evaluation of the efficiency of several desiccants. *J. Org. Chem.* **2010,** *75* (24), 8351–8354.
3. 1-Phenyl-2,3,4,5-Tetramethylphosphole. *Org. Synth.* **1992,** *70*.
4. Fagan, P. J.; Burns, E. G.; Calabrese, J. C., Synthesis of boroles and their use in low-temperature Diels-Alder reactions with unactivated alkenes. *J. Am. Chem. Soc.* **1988,** *110* (9), 2979–2981.
5. Tolentino, D. R.; Neale, S. E.; Isaac, C. J.; Macgregor, S. A.; Whittlesey, M. K.; Jazzar, R.; Bertrand, G., Reductive Elimination at Carbon under Steric Control. *J. Am. Chem. Soc.* **2019,** *141* (25), 9823–9826.
6. Lenz, P.; Oshimizu, R.; Klabunde, S.; Daniliuc, C. G.; Muck-Lichtenfeld, C.; Tendyck, J. C.; Mori, T.; Uhl, W.; Hansen, M. R.; Eckert, H.; Yamaguchi, S.; Studer, A., Oxy-Borylenes as Photoreductants: Synthesis and Application in Dehalogenation and Detosylation Reactions. *Angew. Chem. Int. Ed.* **2022,** *61* (42), e202209391.
7. Arduengo, A. J.; Dias, H. V. R.; Harlow, R. L.; Kline, M., Electronic stabilization of nucleophilic carbenes. *J. Am. Chem. Soc.* **1992,** *114* (14), 5530–5534.
8. Melen, R. L.; Lough, A. J.; Stephan, D. W., Boron azides in Staudinger oxidations and cycloadditions. *Dalton Trans.* **2013,** *42* (24), 8674–8683.
9. Hübner, A.; Diefenbach, M.; Bolte, M.; Lerner, H.-W.; Holthausen, M. C.; Wagner, M., Confirmation of an Early Postulate: B–C–B Two-Electron–Three-Center Bonding in Organo(hydro)boranes. *Angew. Chem. Int. Ed.* **2012,** *51* (50), 12514–12518.
10. Sheldrick, G. M., SHELXT– Integrated space-group and crystal-structure determination. *Acta Cryst. A* **2015,** *71* (1), 3–8.
11. Dolomanov, O. V.; Bourhis, L. J.; Gildea, R. J.; Howard, J. A. K.; Puschmann, H., *OLEX2*: a complete structure solution, refinement and analysis program. *J. Appl. Crystallogr.* **2009,** *42* (2), 339–341.
12. Sheldrick, G. M., A short history of SHELX. *Acta Cryst. A* **2008,** *64* (Pt 1), 112–122.
13. Frisch, M. J.; Trucks, G. W.; Schlegel, H. B.; Scuseria, G. E.; Robb, M. A.; Cheeseman, J. R.; Scalmani, G.; Barone, V.; Petersson, G. A.; Nakatsuji, H.; Li, X.; Caricato, M.; Marenich, A.; Bloino, J.; Janesko, B. G.; Gomperts, R.; Mennucci, B.; Hratchian, H. P.; Ortiz, J. V.; Izmaylov, A. F.; Sonnenberg, J. L.; Williams-Young, D.; Ding, F.; Lipparini, F.; Egidi, F.; Goings, J.; Peng, B.; Petrone, A.; Henderson, T.; Ranasinghe, D.; Zakrzewski, V. G.; Gao, J.; Rega, N.; Zheng, G.; Liang, W.; Hada, M.; Ehara, M.; Toyota, K.; Fukuda, R.; Hasegawa, J.; Ishida, M.; Nakajima, T.; Honda, Y.; Kitao, O.; Nakai, H.; Vreven, T.; Throssell, K.; Montgomery, J. A.; Jr., J. E. P.; Ogliaro, F.; Bearpark, M.; Heyd, J. J.; Brothers, E.; Kudin, K. N.; Staroverov, V. N.; Keith, T.; Kobayashi, R.; Normand, J.; Raghavachari, K.; Rendell, A.; Burant, J. C.; Iyengar, S. S.; Tomasi, J.; Cossi, M.; Millam, J. M.; Klene, M.; Adamo, C.; Cammi, R.; Ochterski, J. W.; Martin, R. L.; Morokuma, K.; Farkas, O.; Foresman, J. B.; Fox, D. J., *Gaussian, Inc., Wallingford CT* **2016**.
14. Neese, F., Software update: The ORCA program system—Version 5.0. *WIREs Comput Mol Sci.* **2022,** *12* (5).

15. Chai, J.-D.; Head-Gordon, M., Long-range corrected hybrid density functionals with damped atom–atom dispersion corrections. *Phys. Chem. Chem. Phys.* **2008,** *10* (44), 6615.
16. McLean, A. D.; Chandler, G. S., Contracted Gaussian basis sets for molecular calculations. I. Second row atoms, Z=11-18. *J. Chem. Phys.* **1980,** *72* (10), 5639–5648.
17. Krishnan, R.; Binkley, J. S.; Seeger, R.; Pople, J. A., Self-consistent molecular orbital methods. XX. A basis set for correlated wave functions. *J. Chem. Phys.* **1980,** *72* (1), 650–654.
18. Francl, M. M.; Pietro, W. J.; Hehre, W. J.; Binkley, J. S.; Gordon, M. S.; Defrees, D. J.; Pople, J. A., Self-consistent molecular orbital methods. XXIII. A polarization-type basis set for second-row elements. *J. Chem. Phys.* **1982,** *77* (7), 3654–3665.
19. Curtiss, L. A.; McGrath, M. P.; Blaudeau, J. P.; Davis, N. E.; Binning, R. C.; Radom, L., Extension of Gaussian-2 theory to molecules containing third-row atoms Ga–Kr. *J. Chem. Phys.* **1995,** *103* (14), 6104–6113.
20. Fukui, K., Formulation of the Reaction Coordinate. *J. Phys. Chem.* **1970,** *74* (23), 4161–4163.
21. Fukui, K., The Path of Chemical Reactions - the IRC Approach. *Acc. Chem. Res.* **1981,** *14* (12), 363–368.
22. Mardirossian, N.; Head-Gordon, M., omegaB97M-V: A combinatorially optimized, range-separated hybrid, meta-GGA density functional with VV10 nonlocal correlation. *J. Chem. Phys.* **2016,** *144* (21), 214110.
23. Weigend, F.; Ahlrichs, R., Balanced basis sets of split valence, triple zeta valence and quadruple zeta valence quality for H to Rn: Design and assessment of accuracy. *Phys. Chem. Chem. Phys.* **2005,** *7* (18), 3297–3305.
24. Weigend, F., Accurate Coulomb-fitting basis sets for H to Rn. *Phys. Chem. Chem. Phys.* **2006,** *8* (9), 1057–1065.
25. Marenich, A. V.; Cramer, C. J.; Truhlar, D. G., Universal Solvation Model Based on Solute Electron Density and on a Continuum Model of the Solvent Defined by the Bulk Dielectric Constant and Atomic Surface Tensions. *J. Phys. Chem. B* **2009,** *113* (18), 6378–6396.
26. Grimme, S.; Hansen, A., A practicable real-space measure and visualization of static electron-correlation effects. *Angew. Chem. Int. Ed.* **2015,** *54* (42), 12308–12313.
27. Bissinger, P., Braunschweig, H., Damme, A., Krummenacher, I., Phukan, A.K., Radacki, K. and Sugawara, S., Isolation of a Neutral Boron-Containing Radical Stabilized by a Cyclic (Alkyl)(Amino)Carbene. *Angew. Chem. Int. Ed.* **2014,** *53*, 7360-7363.
